# A noncoding regulatory RNA Gm31932 induces cell cycle arrest and differentiation in melanoma via the miR-344d-3-5p/*Prc1* (and *Nuf2*) axis

**DOI:** 10.1038/s41419-022-04736-6

**Published:** 2022-04-07

**Authors:** Dan Wang, Jianfei Chen, Bohan Li, Qingling Jiang, Ling Liu, Ziyi Xia, Qiusheng Zheng, Minjing Li, Defang Li

**Affiliations:** 1 Yantai Key Laboratory of Pharmacology of Traditional Chinese Medicine in Tumor Metabolism, School of Integrated Traditional Chinese and Western Medicine, Binzhou Medical University, Yantai, 264003 Shandong PR China; 2 Collaborative innovation platform for modernization and industrialization of regional characteristic traditional Chinese medicine, School of Integrated Traditional Chinese and Western Medicine, Binzhou Medical University, Yantai, 264003 Shandong PR China; 3 Key Laboratory of Xinjiang Endemic Phytomedicine Resources of Ministry of Education, School of Pharmacy, Shihezi University, Shihezi, 832002 Xinjiang PR China

**Keywords:** Cancer, Cell biology

## Abstract

Emerging evidence has shown that long non-coding RNAs (lncRNAs) play an important role in inhibiting tumor cell proliferation and inducing differentiation. In this study, integrative analysis of whole transcriptome sequencing data demonstrated that lncRNA-Gm31932 is significantly decreased in all-trans retinoic acid (ATRA)-induced and sodium 4-phenylbutanoate (PB-4)-induced mouse melanoma B16 cells. Silencing lncRNA-Gm31932 could inhibit B16 cell proliferation, with cell cycle arrest at the G0/G1 phase and obvious differentiation characteristics, e.g., increased cell volume, melanin content and tyrosinase (Tyr) activity. Furthermore, a series of experiments (luciferase reporter assay, RNA pull-down assay, and western blotting) showed that lncRNA-Gm3932 down-regulated *Prc1* and *Nuf2* by competitively sponging miR-344d-3-5p, which subsequently reduced the expression of cell cycle-related proteins CDK2, CDC2, and Cyclin B1, and increased the expression of P21 and P27. Moreover, silencing lncRNA-Gm31932 could significantly inhibit tumor growth in B16 melanoma-bearing mice. Taken together, these results indicate that as a possible signaling pathway for ATRA and PB-4, lncRNA-Gm31932 can induce cell cycle arrest and differentiation via miR-344d-3-5p/*Prc1* (and *Nuf2*) axis.

## Introduction

Melanoma is a highly malignant tumor caused by the excessive proliferation of abnormal melanoma cells. As an aggressive tumor, it has been reported that melanoma accounts for approximately 75% of skin cancer-related deaths [1]. The current conventional treatment methods for melanoma include surgery, chemotherapy, and radiotherapy, but the treatment effect is poor. In particular, patients with advanced melanoma not only have a high mortality rate but also have inherent resistance to radiotherapy and chemotherapy [2]. Therefore, finding new therapeutic methods or molecular targets that can effectively prevent and treat melanoma is an urgent task to be undertaken.

In multicellular organisms, cell proliferation is partly controlled by regulating the process of differentiation [3]. As cancer occurs as a result of defects in cell differentiation, the induction of differentiation therapy is a highly feasible treatment strategy. The induction of tumor differentiation is a biological-based tumor therapy, which reverses the differentiation of malignant tumor cells to normal or near normal cells under the effect of a differentiation inducer. Until now, dozens of drugs have been reported to induce the differentiation of melanoma cells, including theophylline, ATRA, PB-4, cyanidin-3-O-beta-glucopyranoside, and alteronol [4–7]. However, the specific mechanism underlying the complex intracellular molecular regulatory network that induces melanoma differentiation is still unclear.

Recently, with the rapid development of genetics and bioinformatics technology, lncRNAs have received an increasing amount of attention from researchers. Recent studies have shown that LNCRNA SPRY4-IT1 is abnormally highly expressed in melanoma cells. After reducing its expression using RNAi technology, it can lead to growth inhibition, differentiation, and apoptosis of melanoma cells, indicating that SPRY4-IT1 plays an important role in the molecular etiology of human melanoma [8]. Additionally, the competitive endogenous RNA (ceRNA) hypothesis was proposed [9]; and many studies have examined the use of lncRNAs as a ceRNA in the occurrence and development of tumors. For example, lncRNA-CCAT1 can be used as a ceRNA to bind miR-155, thereby inhibiting the differentiation of acute myeloid leukemia HL-60 cells into monocytes [10]. Moreover, the effects of lncRNA on pancreatic cancer, cervical cancer, colon cancer, lung cancer, and other tumors have been reported [11–14]. The above research results suggest that the expression of lncRNAs is closely related to the occurrence, development, and differentiation of tumors, which also provides a new direction for tumor research and treatment.

In this study, we selected two currently recognized differentiation inducers, ATRA and PB-4, to induce the differentiation of mouse melanoma B16 cells. As shown in the schematic below, we used RNA-seq technology to sequence the whole transcriptome of ATRA-treated B16 cells and PB-4-treated B16 cells, and screened differentially expressed lncRNAs, miRNAs, and mRNAs in differentiated B16 cells. Following screening, we used DIANA-LncBase, Starbase 2.0, TargetScan, Miranda, RNAhybrid, and other databases to analyze the binding of the differentially expressed lncRNAs, miRNAs, and mRNAs in ATRA-treated and PB-4-treated B16 cells to construct a ceRNA regulatory network. We next employed a series of experiments to further verify the effect of the selected lncRNA on melanoma differentiation. As a result, we found that as a possible signaling pathway for ATRA and PB-4, lncRNA-Gm31932 can induce cell cycle arrest and differentiation via miR-344d-3-5p/Prc1 (and Nuf2) axis.

**Schematic diagram:**
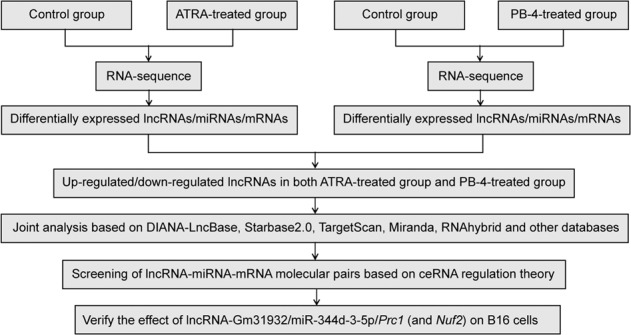
Research ideas for screening lncRNA-miRNA-mRNA molecular pairs by whole transcriptome sequencing.

## Materials and methods

### Reagents

ATRA (molecular weight, 300.44; chemical formula, C_20_H_28_O_2_; purity ≥ 98%) and PB-4 (molecular weight, 186.18; chemical formula, C_10_H_11_NaO_2_; purity ≥ 98%) were purchased from Sigma-Aldrich (St. Louis, MO, USA). Culture medium (DMEM) and fetal bovine serum (FBS) were purchased from Hyclone (Hyclone, Utah, USA). Gm31932 small interfering RNA (siRNA-Gm31932-174, siRNA-Gm31932-118, siRNA-Gm31932-327), Gm31932 short hairpin RNA vector (shRNA-Gm31932), PRC1 small interfering RNA (siRNA-Prc1), NUF2 small interfering RNA (siRNA-Nuf2), corresponding negative control RNA for siRNA-Gm31932 (siRNA-NC), negative control vector for shRNA-Gm31932 (shRNA-NC), miR-344d-3-5p mimics, miR-344d-3-5p inhibitor, and the corresponding negative control RNA (miR-344d-3-5p mimics NC and miR-344d-3-5p inhibitor NC) were all designed and synthesized by Shanghai Integrated Biotech Solutions Co., Ltd. (Shanghai, China), and transfected using the Lipofectamine 3000 transfection reagent (Invitrogen, cat. no. L3000015). Quantitative Real-time PCR Kit (cat. no. SR1110), DNA Content Quantitation Assay (Cell Cycle, cat. no. CA1510) and BCA protein concentration determination kit (cat. no. PC0020) were obtained from Solarbio science & technology Co., Ltd. (Beijing, China). Cell Counting Kit-8 (CCK-8 kit, cat. no. CK04) was purchased from Dojindo Molecular Technologies (Japan), PrimeScript^TM^ reagent Kit with gDNA Eraser (cat. no. RR047A) was purchased from TAKARA BIO INC (Japan). Dual-Luciferase^®^ Reporter Assay System was obtained from Promega Corporation (cat. no. E1910, Madison, USA). RNA labeling mixture (cat. no. 11685597910) was obtained from Roche Molecular Systems, Inc. T7 RNA polymerase (cat. no. E2050S) was obtained from New England Biolabs, Inc. RNA pull-down kit was obtained from BersinBio (cat. no. Bes5102, Guangzhou, China). DAB Detection Kit (Polymer) was obtained from Gene Technology (Shanghai) Co., Ltd. (cat. no. GK600510, Shanghai, China).

### Cell culture and transfection

Mouse melanoma B16 cells were obtained from the Type Culture Collection of the Chinese Academy of Sciences (Shanghai, China) and were authenticated by STR profiling. The cells were cultured in DMEM basal medium, with 10% FBS, 100 U/mL penicillin, and 100 μg/mL streptomycin at 37 °C in a humidified atmosphere of 5% CO_2_. For the transfection experiment, logarithmic phase cells in good growth conditions were transfected using the Lipofectamine 3000 transfection reagent according to the manufacturer’s protocol.

### Cell Counting Kit-8 (CCK-8) assay

Cell proliferation was detected using the CCK-8 kit. Briefly, the transfected cells were seeded in a 96-well plate and cultured in a cell incubator. After culturing for 24, 48, 72, and 96 h, 10 μL of CCK-8 reagent was added to each well, and the culture was continued for 2 h. Then, the optical density (OD) value was determined using a microplate reader (Bio-Rad Laboratories Inc., Hercules, CA, USA) at 450 nm. All experiments were performed in triplicate.

### Colony formation assay

The transfected cells were inoculated onto 6-well chamber slides at approximately 400 cells per well and cultured in a cell culture incubator. After 14 days, the cells were washed with PBS and fixed with 4% paraformaldehyde for 15 min, and subsequently stained with crystal violet staining solution for 30 min at room temperature. Images were taken with a digital camera and clones containing more than 50 cells were counted.

### Morphological assay

To explore whether siRNAs induce differentiation in B16 cells, the transfected cells were seeded on a 6-well plate and cultured in a cell incubator for 72 h. Following incubation, the morphological changes of the cells were observed by a microscope (DMI3000B; Leica Microsystems, Wetzlar, Germany).

### RNA FISH (fluorescence in situ hybridization)

After washing the cells twice with PBS, the cells were fixed with 4% paraformaldehyde for 15 min at room temperature. Then, the cells were permeabilized with 0.1% X-triton 100 for 15 min, and washed twice with PBS. After prehybridization, cells were incubated with probes in the hybridization buffer (before incubation, the probe was mixed with the prehybridization solution at a final concentration of 0.5 μM, denatured at 88 °C for 5 min, and equilibrated at 37 °C for 20 min) at 37 °C overnight. The cells were washed three times with 2 × Saline-Sodium Citrate buffer (SSC), for 5 min per wash. Following washing, 4′,6-diamidino-2-phenylindole (DAPI) was added in the dark at room temperature for 5 min. After washing three times with PBS, the results were examined by a confocal microscope (LSM 880, ZEISS, Germany).

### Melanin content determination

The experimental method for detecting the changes in the melanin content of the cells after transfection was based on the method reported in the previous literature with slight modifications [15, 16]. The transfected cells were inoculated onto 6-well chamber slides and cultured in an incubator for 72 h. Then, the cells were rinsed with PBS buffer three times. After digestion with 0.05% Trypsin-EDTA, the cells were collected and counted, and 200 μL of 1 M NaOH (containing 10% DMSO) was added for 1 h at 80 °C. The absorbances of the melanin contents were measured at 470 nm using a microplate reader after transferring the solution to 96-well plates.

### Tyrosinase activity assay

Tyrosinase activity was estimated by measuring the rate of L-DOPA oxidation as reported earlier with slight modifications [17]. Briefly, the transfected cells were seeded into a 6-well plate and cultured in an incubator for 72 h. After washing the cells with PBS three times, the cells were collected and counted, and 100 μL 0.5% sodium deoxycholate was added to the cells and incubated at 0 °C for 15 min. Following incubation, the cells were mixed with 300 μL of 0.1% L-DOPA in PBS (pH = 6.8) at 37 °C for 2 h. Then, 100 μL of supernatant was added to each well of the 96-well plate and the absorbance at 500 nm was measured. All of the data of the transfected cells were normalized by the mean value of the control cells.

### Cell cycle analysis

The DNA Content Quantitation Assay was employed to examine the cell cycle distribution. In brief, after collecting and fixing in 70% ethanol for 6 h at 4 °C, the cells were washed with PBS three times, and then collected and treated with 100 μL RNase A solution. After 2 h in a 37 °C water bath, 400 μL PI-staining solution was added for 3 h at 4 °C, protected from light. The cell cycle distribution was analyzed by flow cytometry (BD, New Jersey, USA).

### RT-qPCR assay

Total RNA was extracted from culture cells using Trizol reagent based on the manufacturer’s instructions. The OD value of total RNA was detected, and an OD260/OD280 > 1.8 was used for subsequent RT-PCR quantification. Then, a PrimeScript^TM^ reagent Kit with gDNA Eraser was used to reverse-transcribe 1 µg of total RNA in a 20 µL volume into cDNA. Quantitative real-time PCR was performed using the Quantitative Real-time PCR Kit. All primers were designed and synthesized by Shanghai Integrated Biotech Solutions Co., Ltd. (Shanghai, China). The results were normalized using GAPDH or U6 as an internal control.

### Western blotting

RIPA lysis buffer was used to extract protein, and the protein concentration was quantified using the BCA protein concentration determination kit. The proteins (50 μg per sample) were resolved by electrophoresis under 100 V constant pressure and subsequently electro-transferred onto a PVDF transfer membrane. Then, the membrane was incubated in 5% bovine serum albumin (BSA) in TBST buffer at room temperature for 2 h, followed by incubation at 4 °C overnight with the primary antibodies as follows: anti-β-actin (1:5000; ab179467, Abcam), anti-CDC2 (1:1000; ab201008, Abcam), anti-CDK2 (1:1000; ab32147, Abcam), anti-Cyclin B1 (1:2000; ab181593, Abcam), anti-P21 (1:1000; ab188224, Abcam), anti-P27 (1:2000; ab193379, Abcam), anti-Wnt1 (1:500; 27935-1-AP, Proteintech), anti-β-catenin (1:500; ab68183, Abcam), anti-PRC1 (1:5000; ab51248, Abcam), and anti-NUF2 (1:1000; ab230313, Abcam). All antibodies were diluted with a TBST buffer containing 5% BSA. Next, the membranes were washed three times with TBST and incubated with secondary antibodies for 1 h. The membranes were then incubated with ECL chemiluminescence, and the bands were observed using a UVP chemiluminescence imaging system. We used β-actin protein as the internal reference protein to calculate the expression of the protein to be tested.

### Luciferase reporter assay

A dual luciferase reporter assay was used to verify the target relationship between lncRNA-Gm31932 and miR-344d-3-5p, and miR-344d-3-5p and PRC1 (and NUF2). The luciferase gene reporter vectors (Pmirglo-Gm31932-3′ UTR WT, Pmirglo-Gm31932-3′ UTR MUT, Pmirglo-*Prc1*-3′ UTR WT, Pmirglo-*Prc1*-3′ UTR MUT, Pmirglo-*Nuf2*-3′ UTR WT, Pmirglo-*Nuf2*-3′ UTR MUT) and miR-344d-3-5p mimics NC, miR-344d-3-5p mimic and miR-344d-3-5p-mut used in the experiment were all designed and synthesized by Shanghai Integrated Biotech Solutions Co., Ltd. (Shanghai, China). The cells were seeded in 48-well plates and co-transfected with 40 ng of reporter plasmids and 10 pmol of miRNA mimics by Lipofectamine 3000. After 24 h, the luciferase activities were measured using the dual luciferase reporter system (FLUOstar Omega, BMG Labtech, Offenburg, Germany).

### RNA pull-down assay

The RNA pull-down experiment was performed as described previously with slight modifications [18, 19]. Briefly, the biotin-labeled RNA was transcribed with biotin RNA labeling mixture and T7 RNA polymerase, and then treated with RNase-free DNase I and purified. Next, 3 µg of biotinylated RNA was heated to 90 °C for 2 min and then placed on ice for 2 min. The RNA structure buffer and RNase-free water were then added and left at room temperature for 20 min to form the RNA secondary structure. Subsequently, the probe-magnetic beads were prepared according to the instructions of the RNA pull-down kit. The probe-magnetic bead complex was mixed with the cell extract and RNase inhibitor and Poly (dI•dC) were added, before gently rotating at room temperature for 2 h to combine. Finally, the magnetic beads were collected, and 1 mL of ice-cold NT2 buffer was added, before mixing and washing at 4 °C for 5 min. The supernatant was removed, the magnetic beads were washed four times, and the bound RNA in the pull-down material was analyzed by RT-qPCR.

### Animal preparation

Four-week-old C57BL/6 male mice (License No. SYXK(Lu)20180029) were purchased from Jinan Pengyue Experimental Animal Bcreeding Co., Ltd. (Jinan, China). The animals were acclimatized under standard animal care conditions for 7 days before the start of the experiment. All experimental operations followed the requirements of the Animal Ethics and Experimental Safety Committee of Binzhou Medical University and were conducted in accordance with relevant animal experiment guidelines.

### In vivo tumorigenesis assay

Under sterile conditions, B16 cells (3 × 10^6^ cells/animal) were subcutaneously inoculated into the C57BL/6 mice. The inoculated mice were randomly divided into three groups as follows: the control group, the siRNA-NC group, and the siRNA-Gm31932 group (*n* = 8 mice per group). When the tumor size reached 100 mm^3^, the transfection reagent was injected intratumorally. Each mouse in the siRNA-NC group and the siRNA-Gm31932-174 group was injected with 15 μg siRNA, 1.2 μL transfection reagent, and 10 μL glucose solution, to a total volume of 20 μL. After 15 min of incubation at room temperature, intratumoral injection was performed. The mice in the control group were injected intratumor with the same volume of saline. Except for this, the other experimental conditions of the three groups of mice were completely the same. The mice were injected three times a week, weighed every 2 days, and the tumor size was measured by digital caliper. After 2 consecutive weeks, the mice were sacrificed and the tumors were excised for photographing and weighing.

### Immunohistochemistry assay (IHC)

The paraffin-embedded tumor tissue sections were deparaffinized and rehydrated, and EDTA antigen repair solution (pH 9.0) was used for heat-induced antigen recovery. Sections were incubated with anti-Ki-67 (1:1000; ab15580, Abcam), anti-PRC1 (1:100; 15617-1-AP, Proteintech), anti-NUF2 (1:100; ab230313, Abcam) and secondary antibody, and positive signals were visualized by the DAB Detection kit (Polymer). Images were taken by a microscope with 200 × magnification.

### Statistical analysis

The experiment was repeated three times. The data are presented as the means ± SD and a significant difference was determined via a two-tailed unpaired Student’s t-tests or one-way ANOVA followed by the Dunnett correction, and analyses were performed using SPSS 25.0 statistical software. *P* < 0.05 were considered statistically significant.

## Results

### LncRNA-Gm31932 is involved in ATRA- and PB-4-induced B16 cell cycle arrest and differentiation

**Fig. 1:**
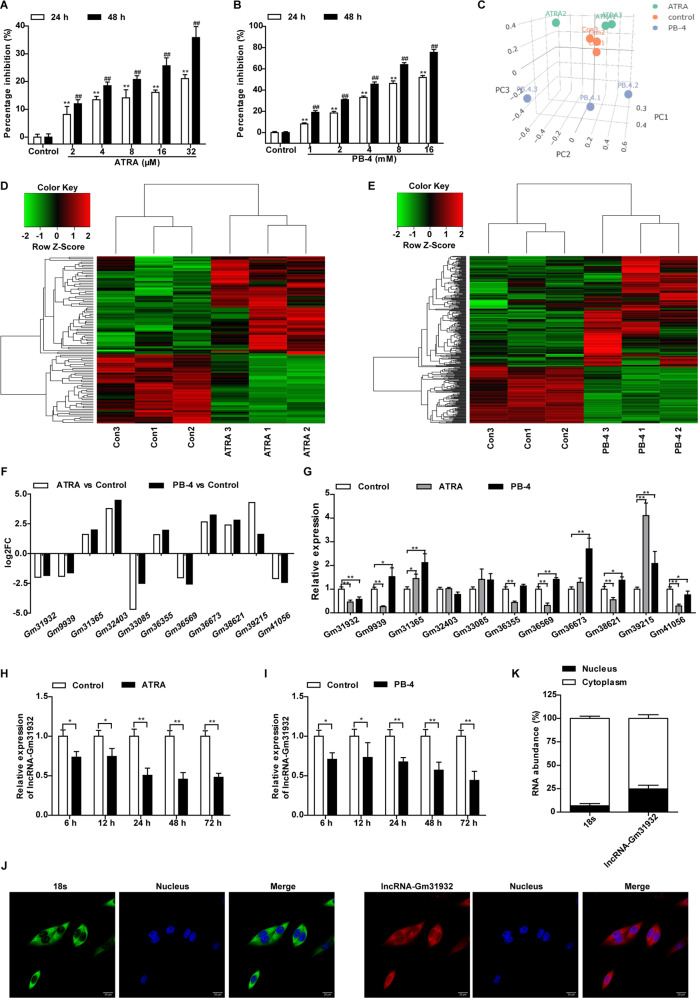
Unique lncRNA expression profile of B16 cells after induced differentiation. **A**, **B** The cell proliferation inhibition rate was detected by CCK-8 assay after treatment with ATRA or PB-4. *n* = 3. Data are presented as the mean ± SD. ^**^P < 0.01 compared with the corresponding control group at 24 h. ^##^*P* < 0.01 compared with the corresponding control group at 48 h by Student’s *t*-test. **C** Principal component analysis of the RNA expression profiles of B16 cells treated with ATRA or PB-4. **D**, **E** Cluster analysis and heat map of differentially expressed lncRNAs in B16 cells induced by ATRA or PB-4. **F** The sequencing results of lncRNAs are indicated. **G** The relative levels of the indicated lncRNAs were determined by RT-qPCR after treatment with ATRA and PB-4. *n* = 4. **H**, **I** RT-qPCR analysis of lncRNA-Gm31932 in B16 cells treated with ATRA or PB-4 for different durations. **J** Detection of the nucleocytoplasmic distribution of lncRNA-Gm31932 by in situ hybridization. **K** Statistics of the nuclear and cytoplasmic distribution of lncRNA-Gm31932. *n* = 3. Data are presented as the mean ± SD. *P < 0.05, **P < 0.01 compared with the corresponding control group by one-way ANOVA followed by the Dunnett correction.

To explore how lncRNAs impact the differentiation of B16 cells, we first selected ATRA and PB-4 to induce cell differentiation. Following treatment with ATRA or PB-4, the B16 cell proliferation rate was significantly lower than that of the control group (Fig. 1A, B). Moreover, B16 cell colony formation was significantly inhibited (Supplementary Fig. 1A–D), and the cell volume increased, showing a typical state of cell differentiation (Supplementary Fig. 1E, F). Additionally, the proportion of cells in the G0/G1 phase increased significantly after ATRA or PB-4 treatment (Supplementary Fig. 1G–J). The melanin content of the cells was significantly increased, and the activity of tyrosinase, a key enzyme for the synthesis of melanin, was significantly increased (Supplementary Fig. 1K–N). Based on the above results, ATRA and PB-4 not only induce B16 cell differentiation but also cause cell cycle arrest at the G0/G1 phase.

The total RNAs of ATRA- or PB-4-treated B16 cells were used to detect differentially expressed RNAs by RNA-seq technology (Fig. 1C). The results showed that 102 lncRNAs, 24 miRNAs, and 761 mRNAs showed differential expression in the ATRA-treated group, and 306 lncRNAs, 105 miRNAs, and 2567 mRNAs showed differential expression in the PB-4-treated group (Supplementary Table 1, Fig. 1D, E). Further analysis showed that 11 lncRNAs had the same expression trend in the ATRA-treated group and PB-4-treated group, with|log2 (fold change)| ≥ 1.5 (Fig. 1F). The levels of these 11 lncRNAs were further validated by RT-qPCR (Fig. 1G and Supplementary Table 2). Considering the length of lncRNAs and the existence of pseudogenes, we finally selected lncRNA-Gm31932 for further research. We found that lncRNA-Gm31932 in B16 cells treated with ATRA or PB-4 were decreased significantly after treatment for 24–72 h (Fig. 1H, I). These results suggest that lncRNA-Gm31932 may be involved in the regulation of cell cycle arrest and differentiation of B16 cells induced by ATRA or PB-4. To further verify the role of lncRNA-Gm31932, we detected its localization by RNA FISH experiments. The results showed that lncRNA-Gm31932 is highly expressed in the cytoplasm of melanoma cells (Fig. 1J, K), indicating that it is mainly located in the cytoplasm of B16 cells.

### Silencing of lncRNA-Gm31932 induces melanoma B16 cell cycle arrest and differentiation

**Fig. 2:**
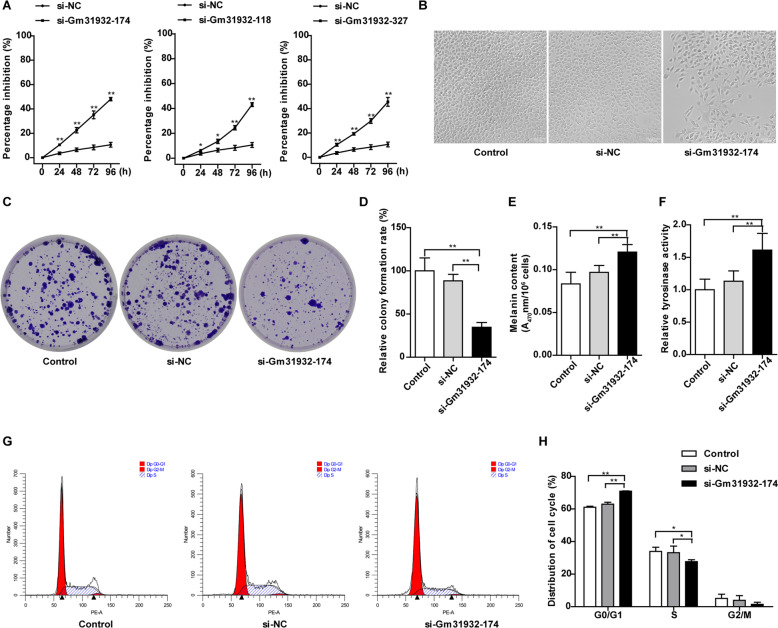
Effect of silencing lncRNA-Gm31932 on B16 cell cycle and differentiation. **A** The cell proliferation inhibition rate was detected by CCK-8 assay. **B** The effect of lncRNA-Gm31932 on morphological changes in B16 cells. **C** Representative images of cell colonies. **D** The colony formation inhibition rate. **E** Changes in the melanin content of B16 cells. **F** Changes in the tyrosinase activity in B16 cells. **G** The effect of lncRNA-Gm31932 on the B16 cell cycle was detected by flow cytometry. **H** Quantitative analysis of cell cycle distribution. *n* = 3. Data are presented as the mean ± SD. *P < 0.05, **P < 0.01 compared with the corresponding control group by Student’s *t*-test.

To explore the biological function of lncRNA-Gm31932, we used small interfering RNAs (siRNAs) to specifically inhibit the expression of lncRNA-Gm31932. We found that the three Gm31932 siRNAs could significantly inhibit the proliferation of B16 cells (Supplementary Fig. 2, Fig. 2A). Among them, siRNA-Gm31932-174 had the most obvious effect, and the inhibition rate reached 35.14% at 72 h. Therefore, siRNA-Gm31932-174 was selected and used for the subsequent experiments. The microscopic data revealed that siRNA-Gm31932-174 transfected B16 cells exhibited obviously morphological changes, including reduced cell density and increased cell volume (Fig. 2B). Meanwhile, the colony formation rate was significantly decreased (Fig. 2C, D), the melanin content and tyrosinase activity was significantly increased (Fig. 2E, F), which suggested that lncRNA-Gm31932 silencing could induce differentiation of B16 cells. Furthermore, flow cytometry data showed that the percentage of B16 cells transfected with siRNA-Gm31932-174 in S phase was slightly lower, but in G0/G1 phase was significantly higher than that of control cells (Fig. 2G, H). This result suggests that lncRNA-Gm31932 silencing can induce B16 cell cycle arrest at the G0/G1 phase. In addition, the short hairpin RNA (shRNA) vector which down-regulated lncRNA-Gm31932 was constructed, and the regulatory effect of shRNA-Gm31932 were examined. Consist with the result from siRNA-Gm31932-174, we also found that silencing of lncRNA-Gm31932 (shRNA-Gm31932) induced melanoma B16 cell cycle arrest and differentiation (Supplementary Fig. 3).

### LncRNA-Gm31932-mediated ceRNA regulation depends on miR-344d-3-5p

**Fig. 3:**
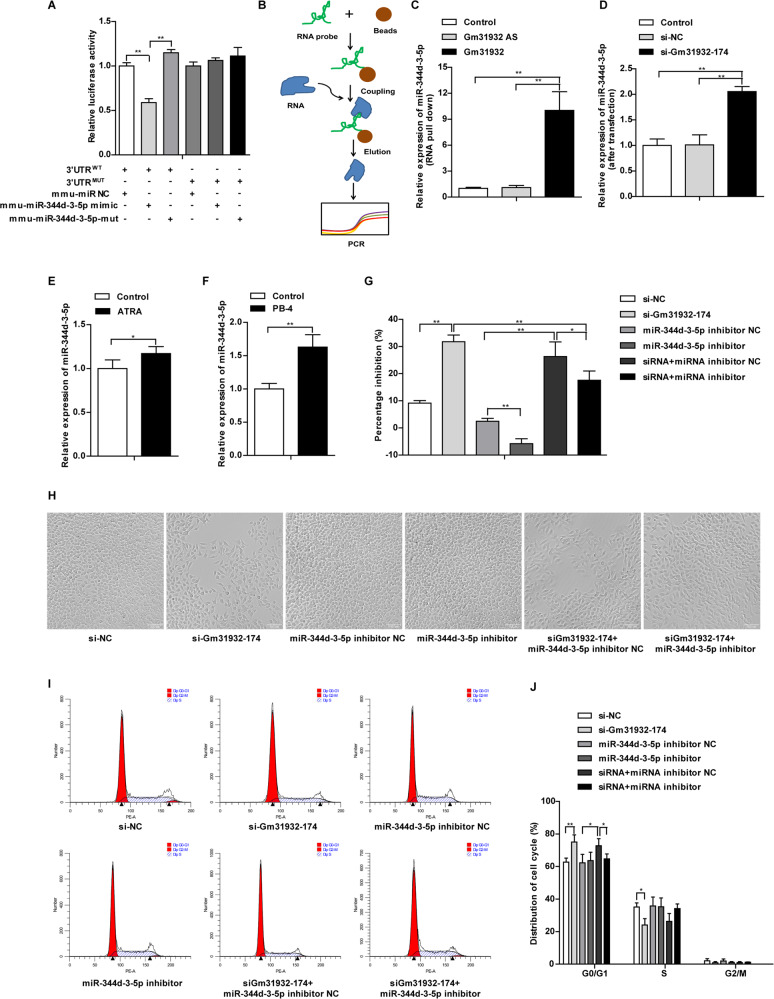
Relationship between lncRNA-Gm31932 and miR-344d-3-5p. **A** Relative luciferase activities of wild-type (WT) and mutated (MUT) reporter gene vectors of lncRNA-Gm31932 in B16 cells co-transfected with miR-344d-3-5p mimic/miR-344d-3-5p mut. **B** Principal flowchart of the RNA pull-down experiment. **C** The miR-344d-3-5p content, which was enriched from the cell lysate by RNA pull down, was detected by RT-qPCR. **D** The RNA expression level of miR-344d-3-5p was determined in lncRNA-Gm31932 silenced cells. **E**, **F** The mRNA expression of miR-344d-3-5p was detected in ATRA- or PB-4-treated B16 cells. **G** The rescue effect of the miR-344d-3-5p inhibitor on the growth inhibition of B16 cells mediated by silencing lncRNA-Gm31932 was determined by CCK-8 assay. **H** The rescue effect of miR-344d-3-5p inhibitor on the morphological changes of B16 cells mediated by silencing lncRNA-Gm31932 was observed using a microscope. **I** The rescue effect of miR-344d-3-5p inhibitor on the cell cycle arrest of B16 cells mediated by silencing lncRNA-Gm31932 was determined by flow cytometry. **J** Quantitative analysis of cell cycle distribution (*n* = 6). Data are presented as the mean ± SD, *n* = 3. *P < 0.05, **P < 0.01 compared with the corresponding control group by Student’s *t*-test.

Considering that lncRNA can be used as ceRNA to competitively bind to miRNA, we used DIANA-LncBase and Starbase 2.0 to analyze the binding of the differentially expressed miRNAs in both ATRA- and PB-4-treated group to lncRNA-Gm31932. In this analysis data, we found that miR-344d-3-5p could bind to lncRNA-Gm31932 (Supplementary Data 7). Then, we constructed the wild-type and mutant luciferase reporter gene vectors of Gm31932 and co-transfected with miR-344d-3-5p mimic/miR-344d-3-5p mut. The luciferase assay showed that miR-344d-3-5p mimic significantly reduced the relative luciferase activity of Gm31932-WT-treated B16 cells, but did not affect that of Gm31932-MUT-treated B16 cells, suggesting that lncRNA-Gm31932 could bind to miR-344d-3-5p (Supplementary Fig. 4A, B, Fig. 3A). Additionally, RNA pull-down assay showed that the level of miR-344d-3-5p in the Gm31932 group was significantly higher than that in the control group and the Gm31932-AS group (Fig. 3B, C). The above experimental data show that lncRNA-Gm31932 acts as a sponge for miR-344d-3-5p. Moreover, silencing Gm31932 could promote the expression of miR-344d-3-5p, which is also consistent with the increased expression of miR-344d-3-5p in B16 cells treated with ATRA and PB-4 (Fig. 3D–F). We next explore whether lncRNA-Gm31932 regulates cell biological functions by sponging miR-344d-3-5p. The results showed that the miR-344d-3-5p inhibitor could reverse the inhibition of cell proliferation, morphological changes, and cell cycle arrest induced by lncRNA-Gm31932 (Fig. 3G–J). In summary, the above results suggested that lncRNA-Gm31932 could directly bind to miR-344d-3-5p and participate in B16 cell cycle arrest and differentiation.

### *Prc1* and *Nuf2* are target genes of miR-344d-3-5p

**Fig. 4:**
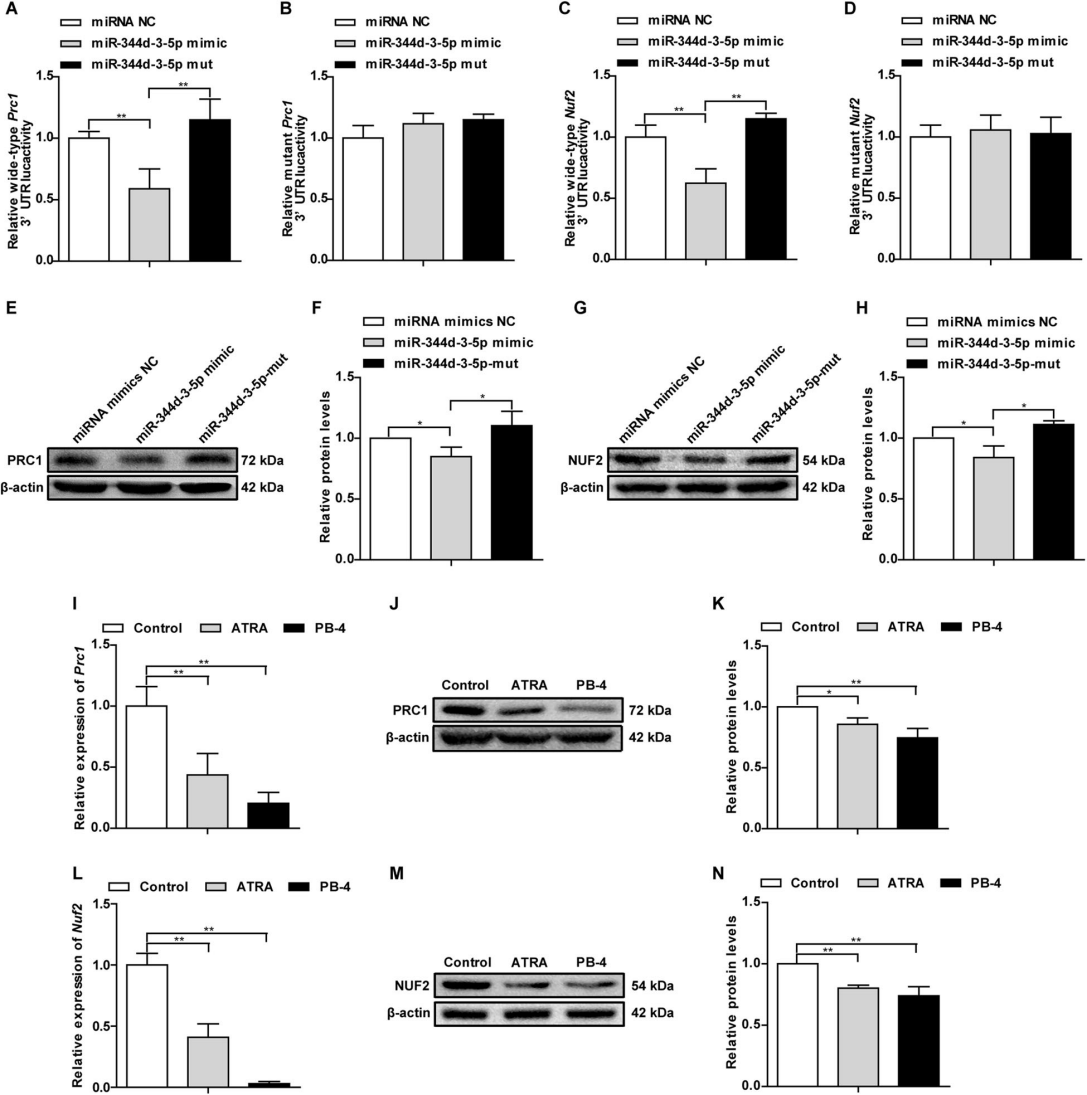
Relationship between miR-344d-3-5p and *Prc1* (and *Nuf2*). **A**, **B** Relative luciferase activities of wild type (WT) and mutated (MUT) *Prc1* reporter gene vectors in B16 cells co-transfected with miR-344d-3-5p mimic/miR-344d-3-5p mut. **C**, **D** Relative luciferase activities of wild-type (WT) and mutated (MUT) *Nuf2* reporter gene vectors in B16 cells co-transfected with miR-344d-3-5p mimics/miR−344d-3-5p mut. **E** PRC1 protein expression following transfection with miR-344d-3-5p mimics/miR-344d-3-5p mut. **F** Quantitative analysis of the protein level of PRC1 in miR-344d-3-5p mimics/miR-344d-3-5p mut-transfected B16 cells. **G** NUF2 protein expression when transfected with miR-344d-3-5p mimics/miR-344d-3-5p mut. **H** Quantitative analysis of the protein level of NUF2 in miR-344d-3-5p mimics/miR-344d-3-5p mut-transfected B16 cells. **I** Quantitative analysis of the mRNA level of *Prc1* in B16 cells after treated with ATRA and PB-4 by RT-qPCR. **J** PRC1 protein expression in B16 cells after treatment with ATRA and PB-4. **K** Quantitative analysis of the protein level of PRC1 in B16 cells following treatment with ATRA and PB-4. **L** Quantitative analysis of the mRNA level of *Nuf2* in B16 cells after treatment with ATRA and PB-4 by RT-qPCR. **M** NUF2 protein expression in B16 cells after treatment with ATRA and PB-4. **N** Quantitative analysis of the protein level of NUF2 in B16 cells after treatment with ATRA and PB-4. *n* = 3. Data are presented as the mean ± SD. *P < 0.05, **P < 0.01 compared with the corresponding control group by Student’s *t*-test.

Through integrative analysis of the sequencing data, we found that *Prc1* and *Nuf2* are potential target genes of miR-344d-3-5p (Supplementary Data 7). PRC1 is a chromatin-based gene transcription repressor [20]. As a key regulator of cell proliferation and apoptosis, PRC1 can act on a variety of tumors [21]. NUF2 is located on chromosome 1 q23.3 and is also plays a vital role in the occurrence of a variety of tumors [22, 23]. Therefore, we explored the validity of these results using the dual-luciferase reporter assay. The results showed that when the miR-344d-3-5p mimic was co-transfected with *Prc1*-WT or *Nuf2*-WT, luciferase activity was significantly reduced (Supplementary Fig. 4C–F, Fig. 4A, D). Western blotting showed that miR-344d-3-5p mimic obviously reduced the levels of PRC1 and NUF2 (Fig. 4E–H). Additionally, RT-qPCR data and western blotting showed that the mRNA and protein levels of PRC1 and NUF2 in B16 cells treated with ATRA or PB-4 were significantly lower than those of the control group (Fig. 4I–N). These results suggest that *Prc1* and *Nuf2* are direct target genes for miR-344d-3-5p.

### PRC1- and NUF2-associated pathways are regulated by lncRNA-Gm31392

**Fig. 5:**
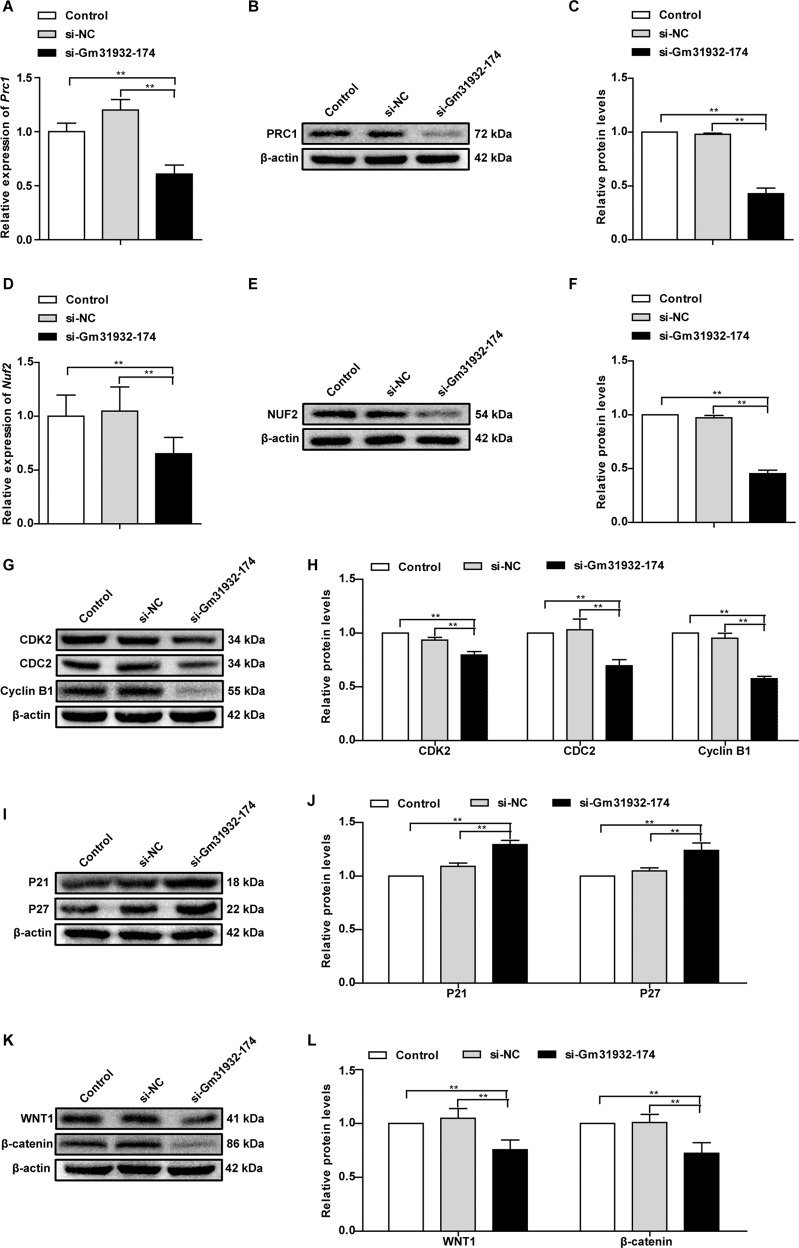
Effect of silencing lncRNA-Gm31932 on PRC1- and NUF2-associated pathways. **A** Quantitative analysis of the mRNA level of *Prc1* in B16 cells after silencing lncRNA-Gm31932 by RT-qPCR. **B** PRC1 protein expression in B16 cells after silencing lncRNA-Gm31932. **C** Quantitative analysis of the protein level of PRC1 after silencing lncRNA-Gm31932. **D** Quantitative analysis of the mRNA level of *Nuf2* in B16 cells after silencing lncRNA-Gm31932 by RT-qPCR. **E** NUF2 protein expression in B16 cells after silencing lncRNA-Gm31932. **F** Quantitative analysis of the protein level of NUF2 after silencing lncRNA-Gm31932. **G** Western blotting for CDK2, CDC2, and Cyclin B1 in B16 cells after transfection with siRNA-Gm31932-174. **H** Quantitative analysis of the protein levels of CDK2, CDC2, and Cyclin B1 in B16 cells after transfection with siRNA-Gm31932-174. **I** Western blotting of P21 and P27 in B16 cells after transfection with siRNA-Gm31932-174. **J** Quantitative analysis of the protein level of P21 and P27 in B16 cells after transfection with siRNA-Gm31932-174. **K** Western blotting of WNT1 and β-catenin in B16 cells after transfection with siRNA-Gm31932. **L** Quantitative analysis of the protein level of WNT1 and β-catenin in B16 cells after transfection with siRNA-Gm31932. *n* = 3. Data are presented as the mean ± SD. **P < 0.01 compared with the corresponding control group by one-way ANOVA followed by the Dunnett correction.

We next further investigated the relationship between lncRNA-Gm31932 and PRC1/NUF2 in B16 cells. The data demonstrated that silencing lncRNA-Gm31932 significantly inhibited the expression of PRC1 and NUF2 (Fig. 5A–F). Combined with previous reports showing that PRC1 and NUF2 can cause cell cycle arrest [21, 24], we tested the expression levels of cycle-related proteins. Our results showed that the levels of cycle-related proteins CDK2, CDC2, and Cyclin B1 decreased after silencing lncRNA-Gm31932, while the levels of P21 and P27 increased (Fig. 5G–J). Moreover, because PRC1 is a key component in the activation of the Wnt/β-catenin pathway [25], we also examined the levels of WNT1 and β-catenin. The results showed that the levels of WNT1 and β-catenin were also decreased when lncRNA-Gm31932 was silenced (Fig. 5K, L). Taken together, these results imply that lncRNA-Gm31932 regulates the levels of cycle-related proteins and Wnt/β-catenin pathway molecules via the miR-344d-3-5p/PRC1 (and NUF2) axis, thereby inducing B16 cell cycle arrest and differentiation.

### Silencing of lncRNA-Gm31932 inhibits melanoma growth

**Fig. 6:**
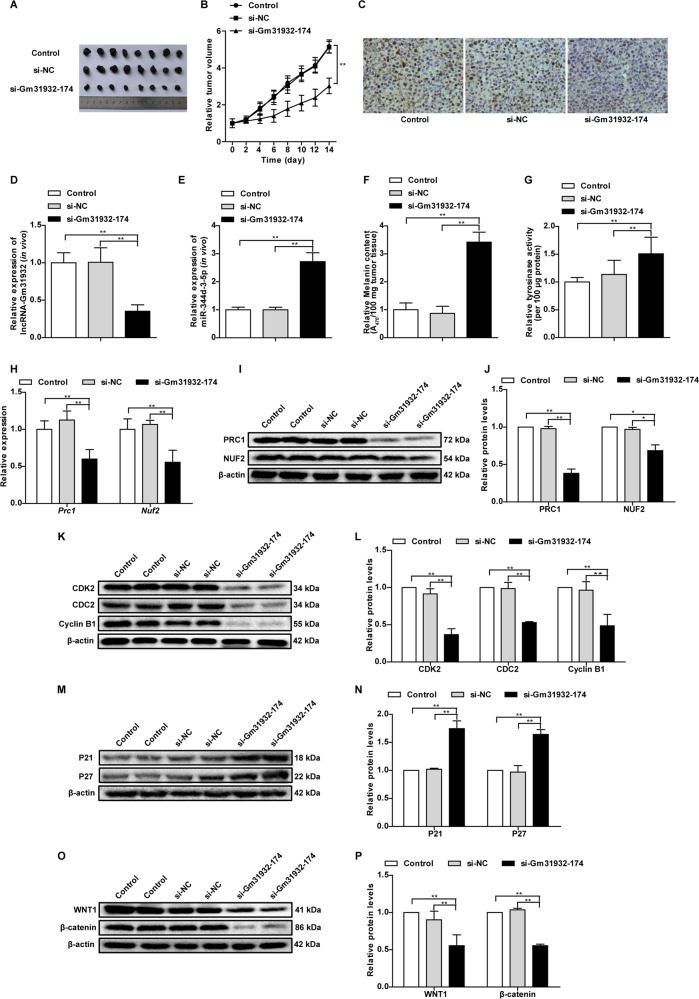
Effect of silencing lncRNA-Gm31932 on tumor growth and size. **A** Representative images of tumor morphology. **B** Data analysis of tumor volume. **C** Representative IHC staining of Ki-67 expression in tumors. **D** Quantitative expression analysis of lncRNA-Gm31932 in tumors by RT-qPCR. **E** Quantitative expression analysis of miR-344d-3-5p in tumors by RT-qPCR. **F** Changes in the melanin content of tumors. **G** Changes in the tyrosinase activity of tumors. **H** Quantitative expression analyses of *Prc1* and *Nuf2* in tumors by RT-qPCR. **I** The protein levels of PRC1 and NUF2 in tumors were determined by western blot. **J** Quantitative analysis of the protein levels of PRC1 and NUF2 in tumors. **K** Western blotting for CDK2, CDC2, and Cyclin B1 in tumors. **L** Quantitative analysis of protein levels of CDK2, CDC, and Cyclin B1 in tumors. **M** Western blotting of P21 and P27 in tumors. **N** Quantitative analysis of the protein level of P21 and P27 in tumors. **O** Western blotting for WNT1 and β-catenin in tumors. **P** Quantitative analysis of the protein level of WNT1 and β-catenin in tumors. *n* = 8. Data are presented as the mean ± SD. *P < 0.05, **P < 0.01 compared with the corresponding control group by Student’s *t*-test.

Next, a tumor xenograft experiment was performed to verify whether lncRNA-Gm31932 can inhibit melanoma growth in mice. To this end, siRNA-Gm31932-174 was injected into mouse tumors by intratumoral injection. Our results showed that the tumor volume and weight of mice in the siRNA-Gm31932-174 treated group was significantly smaller than that of the control and siRNA-NC groups (Fig. 6A, B, Supplementary Fig. 5). Immunohistochemical staining showed that the expression of Ki-67 in tumor tissues in the siRNA-Gm31932-174 treated group was significantly reduced (Fig. 6C). Moreover, RT-qPCR data showed that the level of lncRNA-Gm31932 in tumor tissues was decreased, while that of miR-344d-3-5p was increased in the siRNA-Gm31932-174 treated group (Fig. 6D, E). Additionally, we found that the melanin content and tyrosinase activity of the tumor tissues in the siRNA-Gm31932-174 treated group were significantly higher than those in the control and siRNA-NC groups (Fig. 6F, G).

Next, we detected the levels of PRC1 and NUF2 through RT-qPCR, western blotting and Immunohistochemical, and found that the expression of PRC1 and NUF2 in the tumor tissues of the siRNA-Gm31932-174 treated group was significantly reduced (Fig. 6H–J, Supplementary Fig. 7), which reconfirmed the results of in vitro experiments. Similarly, the levels of CDK2, CDC2, and Cyclin B1 protein in the siRNA-Gm31932-174 treated group were significantly decreased, while the levels of P21 and P27 were increased (Fig. 6K–N). Moreover, the levels of Wnt1 and β-catenin in the tumor tissues of the siRNA-Gm31932-174 treated group were significantly lower compared to those of the control group and the siRNA-NC group (Fig. 6O, P). These results further suggest that silencing lncRNA-Gm31932 can inhibit melanoma growth through the miR-344d-3-5p/*Prc1* (and *Nuf2*) axis.

## Discussion

Melanoma plays a key role in deaths associated with skin cancer [26], and the number of patients with malignant melanoma continues to increase worldwide [27]. Therefore, it is urgent to establish effective therapeutic targets and methods for the treatment of melanoma. Considering that one of the main characteristics of malignant tumor cells is the low degree of differentiation, elucidating the molecular mechanism that induces the differentiation of melanoma is of great significance for differentiation therapy. Extensive research has demonstrated that different lncRNA expression patterns can regulate the cell cycle, proliferation, and differentiation [28]. For example, lncRNA-PCAT1 negatively regulates miR-145-5p to promote osteogenic differentiation [29], while LncRNA-TUG1 can promote osteogenic differentiation of various cells, including TSPCs by promoting bFGF ubiquitination [30]. In this study, we performed whole-transcriptome sequencing analysis on B16 cells treated with ATRA and PB-4, and screened the lncRNAs that were up- or down-regulated in the two groups. Our results showed that lncRNA-Gm31932 may be involved in the differentiation and cycle arrest of B16 cells induced by ATRA and PB-4. Further research found that lncRNA-Gm31932 is mainly distributed in the cytoplasm of B16 cells, and silencing the expression of lncRNA-Gm31932 can inhibit cell proliferation, induce B16 cell differentiation and cycle arrest.

Many studies have shown that lncRNAs and miRNAs can interact, cross-regulate, and participate in the field of oncology. Moreover, studies have shown that LncRNA-MEG3 can promote the growth, metastasis, and formation of melanoma by regulating the miR-21/E-cadherin axis [31]. Considering that lncRNA can be used as a ceRNA to competitively bind to miRNA, we further searched and verified miRNAs that can bind to lncRNA-Gm31932. According to the combined analysis of the whole transcriptome sequencing results, dual luciferase reporter assay, and RNA pull-down assay, we found that lncRNA-Gm31932 can bind to miR-344d-3-5p. This result indicates that lncRNA-Gm31932-mediated ceRNA regulation is dependent on miR-344d-3-5p.

LncRNAs and miRNAs play an important regulatory role in cell proliferation and cell cycle through transcription or post-transcription targeting their mRNAs [32]. Therefore, we further explored the downstream molecules of lncRNA-Gm31932/miR-344d-3-5p involved in cell cycle arrest and differentiation of B16 cells. Through the combined analysis of the sequencing data, and a series of experiments, including dual luciferase reporter analysis, RT-qPCR, and western blot, we found that *Prc1* and *Nuf2* are potential target genes of miR-344d-3-5p. These results were consistent with the changes of *Prc1* and *Nuf2* expression in B16 cells treated with ATRA and PB-4. Additionally, after silencing lncRNA-Gm31932, the expression of PRC1 and NUF2 was significantly decreased. Considering the above research results comprehensively, PRC1 and NUF2 may act as downstream molecules of lncRNA-Gm31932/miR-344d-3-5p and participate in the induction of B16 cell cycle arrest and differentiation.

PRC1 is considered a substrate of a variety of cyclin dependent kinases (CDKs), and many cell-cycle related factors can interact with PRC1. For example, TOPK can form a kinase-substrate complex with CDK1 (also known as CDC2)/Cyclin B1 and PRC1 on the microtubules during mitosis, thereby enhancing the CDK1/Cyclin B1-dependent phosphorylation of PRC1, and strongly promoting cytokinesis [33]. Moreover, studies have confirmed that the Linc-ASEN-UPF1 complex inhibits p21 transcription by recruiting PRC1 and PRC2 to the P21 site, thereby preventing the binding of the transcription activator P53 to the P21 promoter [34]. Other studies have shown that P27 may participate in the regulation of mitotic processes in a CDK-independent manner by regulating PRC1 activity [35]. The results of these previous studies are consistent with our experimental results. We found that silencing the expression of lncRNA-Gm31932 in B16 cells can inhibit the expression of PRC1 and inhibit the proliferation of B16 cells. Moreover, the cell cycle was arrested in the G0/G1 phase, and the expression levels of cycle-related proteins CDK2, CDC2, and Cyclin B1 were decreased, while those of P21 and P27 were increased.

Additionally, it has been reported that silencing the expression of *Prc1* can inhibit the Wnt/β-catenin signaling pathway, inhibit the proliferation, invasion, and angiogenesis of retinoblastoma cells [36]. Considering that the Wnt/β-catenin signaling pathway is also closely related to cell differentiation, we detected the expression of WNT1 and β-catenin, and found that silencing lnRNA-Gm31932 in B16 cells significantly reduced the expression of WNT1 and β-catenin. Taken together, these experimental results indicate that lncRNA-Gm31932 can participate in the regulation of B16 cell cycle arrest and differentiation by inhibiting the expression of PRC1.

Furthermore, the expression of NUF2 is closely related to the occurrence and development of tumors. According to previous reports, silencing *Nuf2* by RNA interference can inhibit the proliferation of pancreatic cancer cells and cause cell cycle arrest in the G0/G1 phase by inhibiting Cyclin B1, CDC2, and Cdc25A [24]. Additionally, inhibiting the expression of *Nuf2* can slow down the growth of glioma, liver cancer, colorectal cancer, and gastric cancer [23, 37, 38]. Moreover, down-regulating the expression of *Nuf2* in Saos-2 cells will result in a significant increase in the levels of cyclin-dependent kinase inhibitors p21cip1 and p27kip1 [39]. These reports are consistent with the results of this experiment, indicating that lncRNA-Gm31932 can participate in the regulation of B16 cell cycle arrest and differentiation by inhibiting the expression of *Nuf2*. Considering that reducing the expression of PRC1 and NUF2 can inhibit the proliferation of melanoma B16 cells, induce cell cycle arrest, and increase melanin content and tyrosinase activity. We believe that PRC1 and NUF2 can be used as targets for the treatment of melanoma.

The results of our in vivo experiment were consistent with those of the in vitro experiment. Silencing lncRNA-Gm31932 not only inhibits the growth and size of tumors, but also increase the melanin content and tyrosinase activity in tumor tissues. Additionally, the expression of lncRNA-Gm31932, miR-344d-3-5p, PRC1, NUF2, cycle-related proteins, and Wnt/β-catenin pathway-related proteins in tumor tissues is consistent with the in vivo experiments. However, there is a limitation to this study. The species conservation of Gm31932 between human and mouse species were analyzed, and we found that the conservation of the lncRNA-Gm31932 sequence is poor. Subsequently, we examined the regulatory effect of Prc1 and Nuf2 in human melanoma A375 cells, respectively. The data showed that silencing of Prc1 and silencing of Nuf2 both inhibited cell proliferation, cell colony formation and induced cell differentiation in A375 cells (Supplementary Fig. 6A–G). Furthermore, silencing of Nuf2 also induced A375 cell cycle arrest at the G0/G1 phase (Supplementary Fig. 6H, I). Based on the above data, PRC1 and NUF2 could be used as targets for the clinical treatment of melanoma. Taken together, all the above results confirmed that as a possible signaling pathway for ATRA and PB-4, lncRNA-Gm31932 can induce cell cycle arrest and differentiation via miR-344d-3-5p/*Prc1* (and *Nuf2*) axis, and provide a new direction for future research.

### Reporting summary

Further information on research design is available in the Nature Research Reporting Summary linked to this article.

## Supplementary information

Supplementary Figure Legends

Reporting Summary aj-checklist

Figure 4 WB Supplementary materials

Figure 5 WB Supplementary materials

Figure 6 WB Supplementary materials

Supplementary Table 1

Supplementary Table 2

Supplementary Figure 1

Supplementary Figure 2

Supplementary Figure 3

Supplementary Figure 4

Supplementary Figure 5

Supplementary Figure 6

Supplementary Figure 7

Supplementary Data 1

Supplementary Data 2

Supplementary Data 3

Supplementary Data 4

Supplementary Data 5

Supplementary Data 6

Supplementary Data 7

## Data Availability

Raw and normalized data files for whole transcriptome sequencing analysis have been deposited in the NCBI Gene Expression Omnibus under accession number GSE180667.
