# Supplementary material for: A noncoding regulatory RNA Gm31932 induces cell cycle arrest and differentiation in melanoma via the miR-344d-3-5p/Prc1 (and Nuf2) axis
Source: Cell Death Dis. 2022 Apr 7;13(4):314. doi: 10.1038/s41419-022-04736-6 (PMC8990078; doi:10.1038/s41419-022-04736-6)
Supplement: Supplementary file 1 — Supplementary Figure Legends [file 41419_2022_4736_MOESM1_ESM.docx]

**Supplementary Figure 1. Effect of ATRA and PB-4 on B16 cells.** (A) Representative images of cell colonies after ATRA treatment. (B) Colony formation inhibition rate after ATRA treatment. (C) Representative images of cell colonies after PB-4 treatment. (D) The colony formation inhibition rate after PB-4 treatment. (E) Effect of ATRA on morphological changes in B16 cells. (F) Effect of PB-4 on morphological changes in B16 cells. (G) ATRA-treated cells were harvested to measure the cell cycle distribution by ﬂow cytometry. (H) Quantitative analysis of the cell cycle distribution after ATRA treatment. (I) PB-4-treated cells were harvested to measure the cell cycle distribution by ﬂow cytometry. (J) Quantitative analysis of cell cycle distribution after PB-4 treatment. (K) Changes in the melanin content of B16 cells after ATRA treatment. (L) Changes in the melanin content of B16 cells after PB-4 treatment. (M) Changes in the tyrosinase activity of B16 cells after ATRA treatment. (N) Changes in the tyrosinase activity of B16 cells after PB-4 treatment. n=3. Data are presented as the mean ± SD. ***P* < 0.01 compared with the corresponding control group by Student’s t test.

**Supplementary Figure 2. Representative pictures of B16 cells transfected with siRNAs.** 5 carboxy-fluorescein (FAM) was used to label siRNA, and 6 hours after transfection with siRNAs, B16 cells were observed under the microscope (si-NC, si-Gm31931-174, Gm31931-118, Gm31931-327).

**Supplementary Figure 3. Effect of shRNA-Gm31932 on B16 cell cycle and differentiation.** (A) The mRNA expression of lncRNA-Gm31932 was detected in shRNA-treated B16 cells. (B) The cell proliferation inhibition rate was detected by CCK-8 assay. (C) The effect of shRNA-Gm31932 on morphological changes in B16 cells. (D) Representative images of cell colonies. (E) The colony formation inhibition rate. (F-G) Changes in the melanin content and the tyrosinase activity of B16 cells. (H) The effect of shRNA-Gm31932 on the B16 cell cycle was detected by flow cytometry. (I) Quantitative analysis of cell cycle distribution. n=3. Data are presented as the mean ± SD. **P* < 0.05, ***P* < 0.01 compared with the corresponding control group by Student’s t test.

**Supplementary Figure 4. Design of binding sites and sequences of lncRNA-Gm31932, miR-344d-3-5p, PRC1, and NUF2.** (A) Prediction of the binding site of miR-344d-3-5p and Gm31932 3'UTR region. (B) Sequence design of Gm31932 3'UTR, Gm31932 3'UTR-Mut, miR-344d-3-5p mimics, and miR-344d-3-5p mut. (C) Prediction of the binding site of miR-344d-3-5p and *Prc1* 3'UTR region. (D) Sequence design of *Prc1* 3'UTR, *Prc1* 3'UTR-Mut, miR-344d-3-5p mimic, and miR-344d-3-5p mut. (E) Prediction of the binding site of miR-344d-3-5p and *Nuf2* 3'UTR region. (F) Sequence design of *Nuf2* 3'UTR, *Nuf2* 3'UTR-Mut, miR-344d-3-5p mimic, and miR-344d-3-5p mut.

**Supplementary Figure 5**. **Effect of silencing lncRNA-Gm31932 on tumor growth.** Data analysis of tumor mass.

**Supplementary Figure 6**. **Effect of silencing PRC1 and NUF2 on A375 cell cycle and differentiation.** (A) Quantitative analysis of the mRNA level of *Prc1* and *Nuf2* in A375 cells after silencing Prc1 or Nuf2 by RT-qPCR. (B) The cell proliferation inhibition rate was detected by CCK-8 assay. (C) The effect of siRNA-PRC1 and siRNA-NUF2 on morphological changes in A375 cells. (D) Representative images of cell colonies. (E) The colony formation inhibition rate. (F-G) Changes in the melanin content and the tyrosinase activity in A375 cells. (H) The effect of siRNA-PRC1 and siRNA-NUF2 on the A375 cell cycle was detected by flow cytometry. (I) Quantitative analysis of cell cycle distribution. n=3. Data are presented as the mean ± SD. **P* < 0.05, ***P* < 0.01 compared with the corresponding control group by Student’s t test.

**Supplementary Figure 7. Effect of silencing lncRNA-Gm31932 on PRC1 and NUF2.** Representative IHC staining of Prc1 and Nuf2 expression in tumors.
