# Supplementary figures and images for: A noncoding regulatory RNA Gm31932 induces cell cycle arrest and differentiation in melanoma via the miR-344d-3-5p/Prc1 (and Nuf2) axis
Source: Cell Death Dis. 2022 Apr 7;13(4):314. doi: 10.1038/s41419-022-04736-6 (PMC8990078; doi:10.1038/s41419-022-04736-6)

Fig 4E


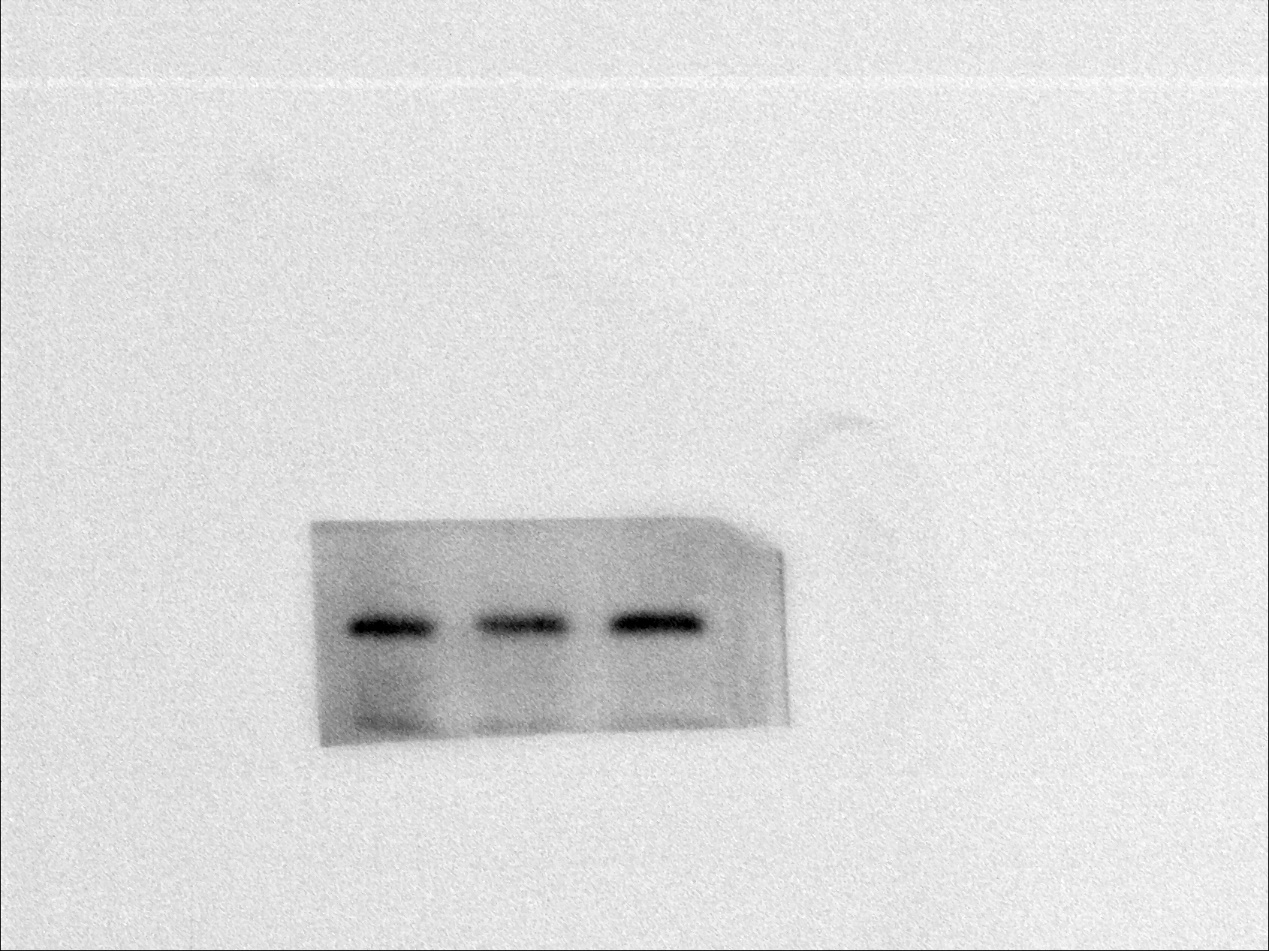


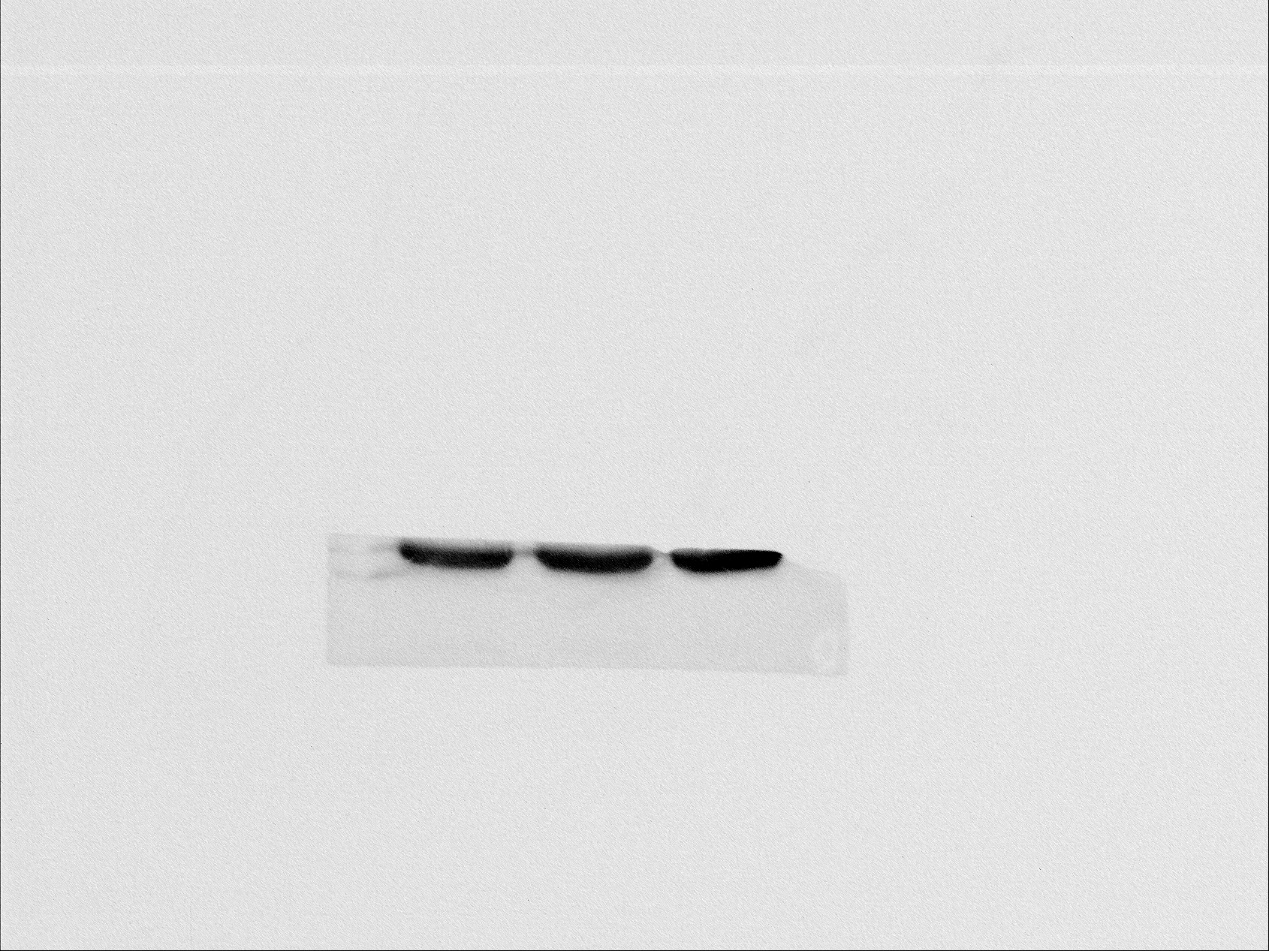


Fig 4G


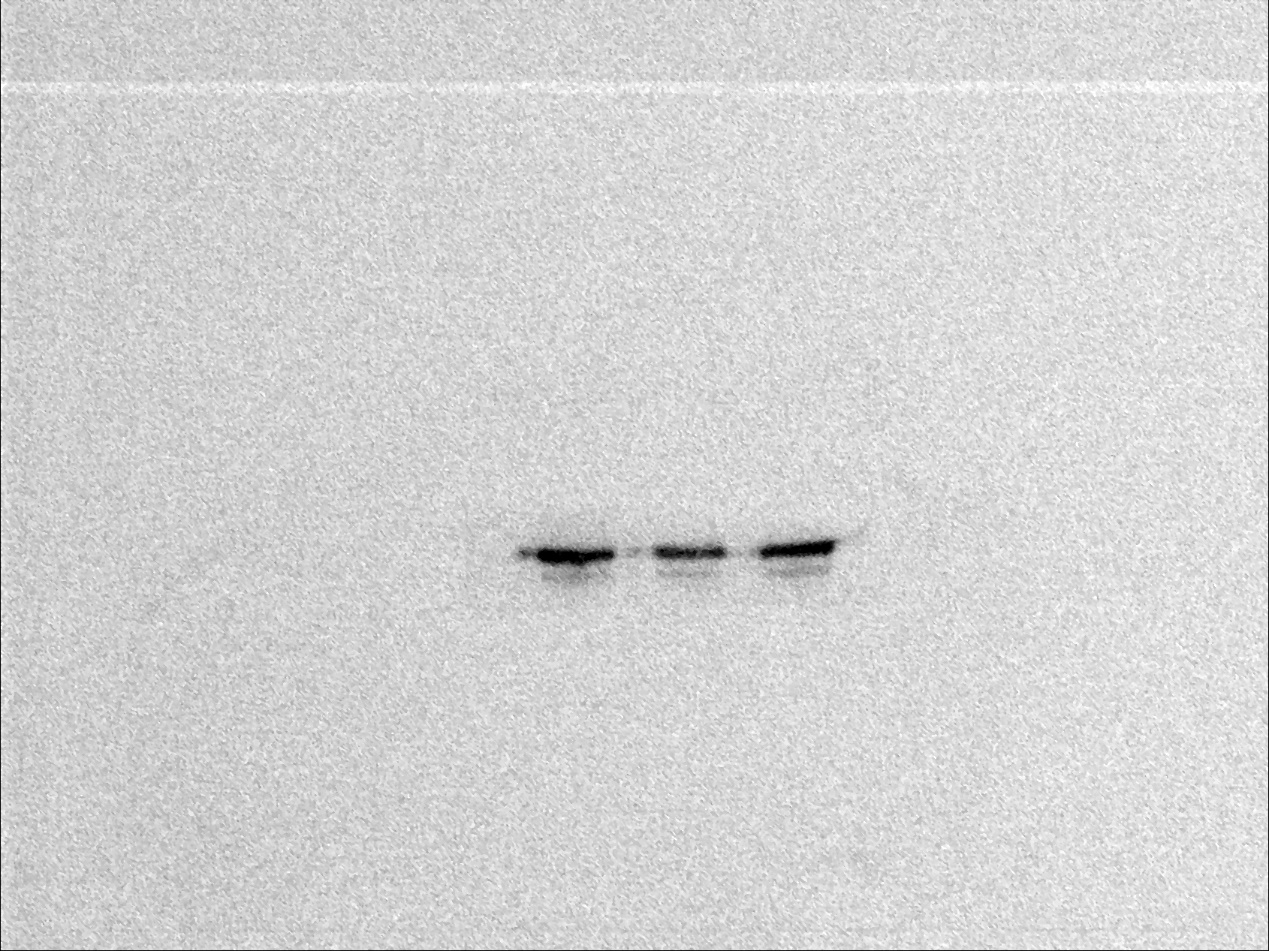


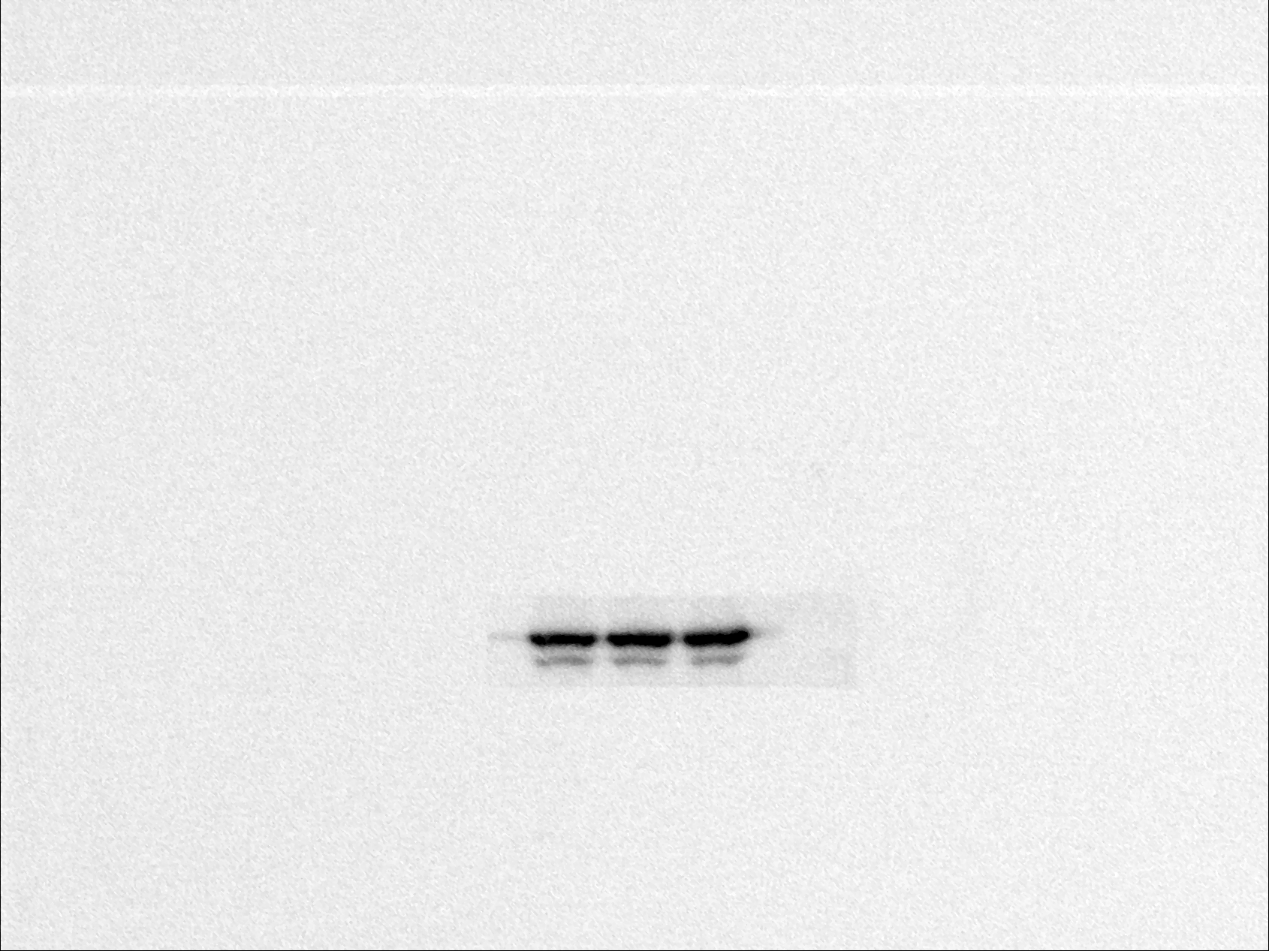


Fig 4J


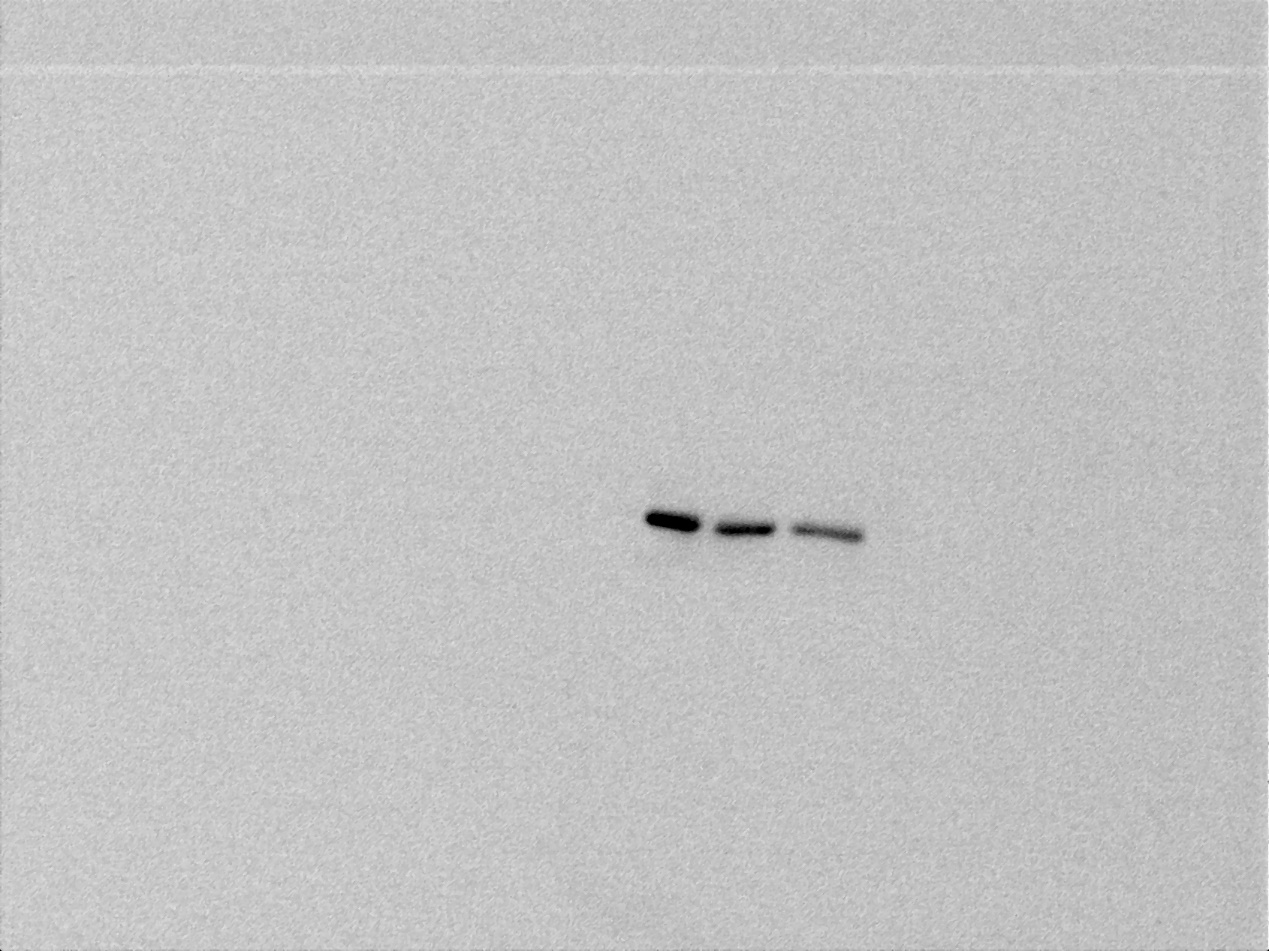


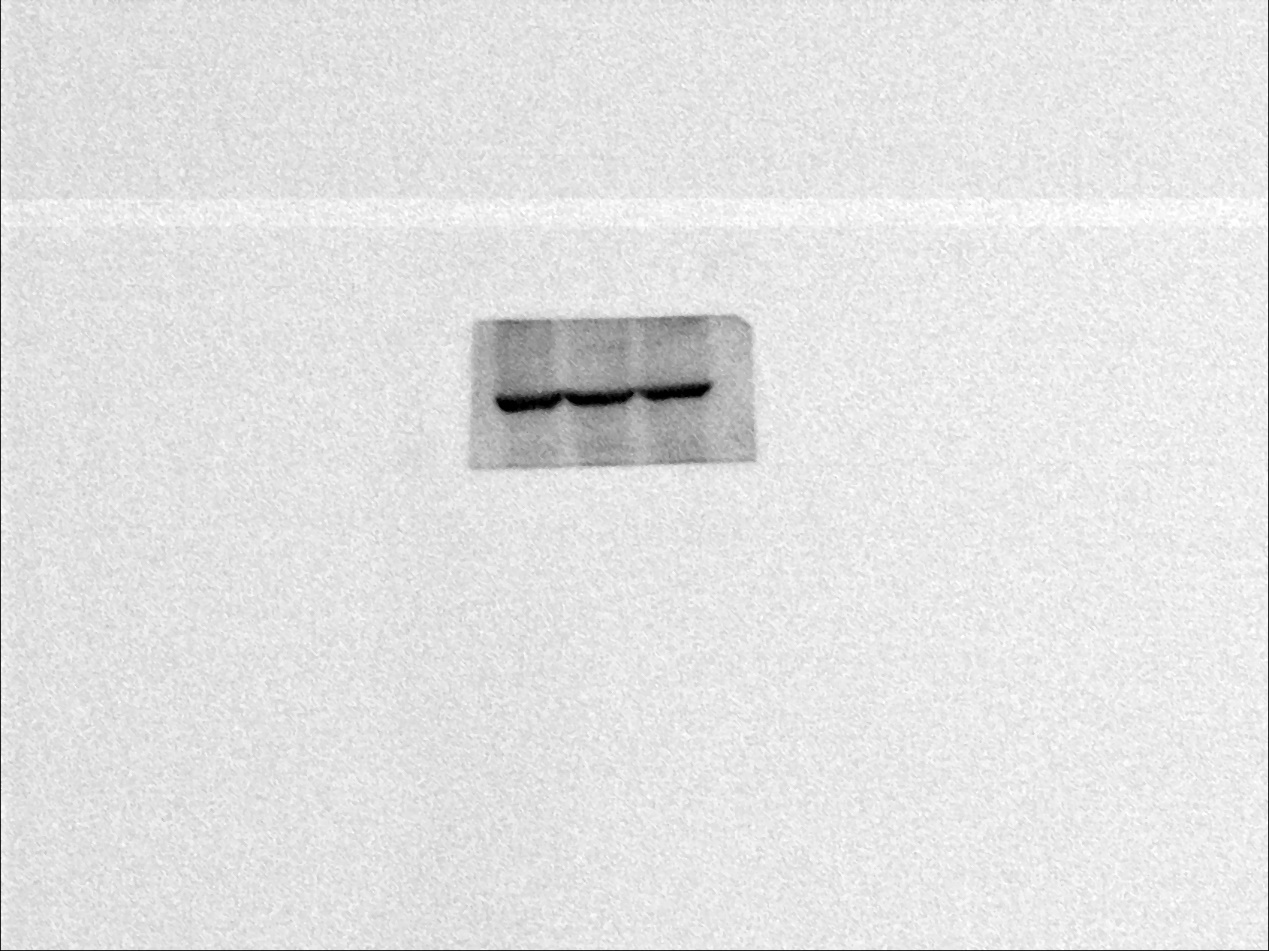


Fig 4M


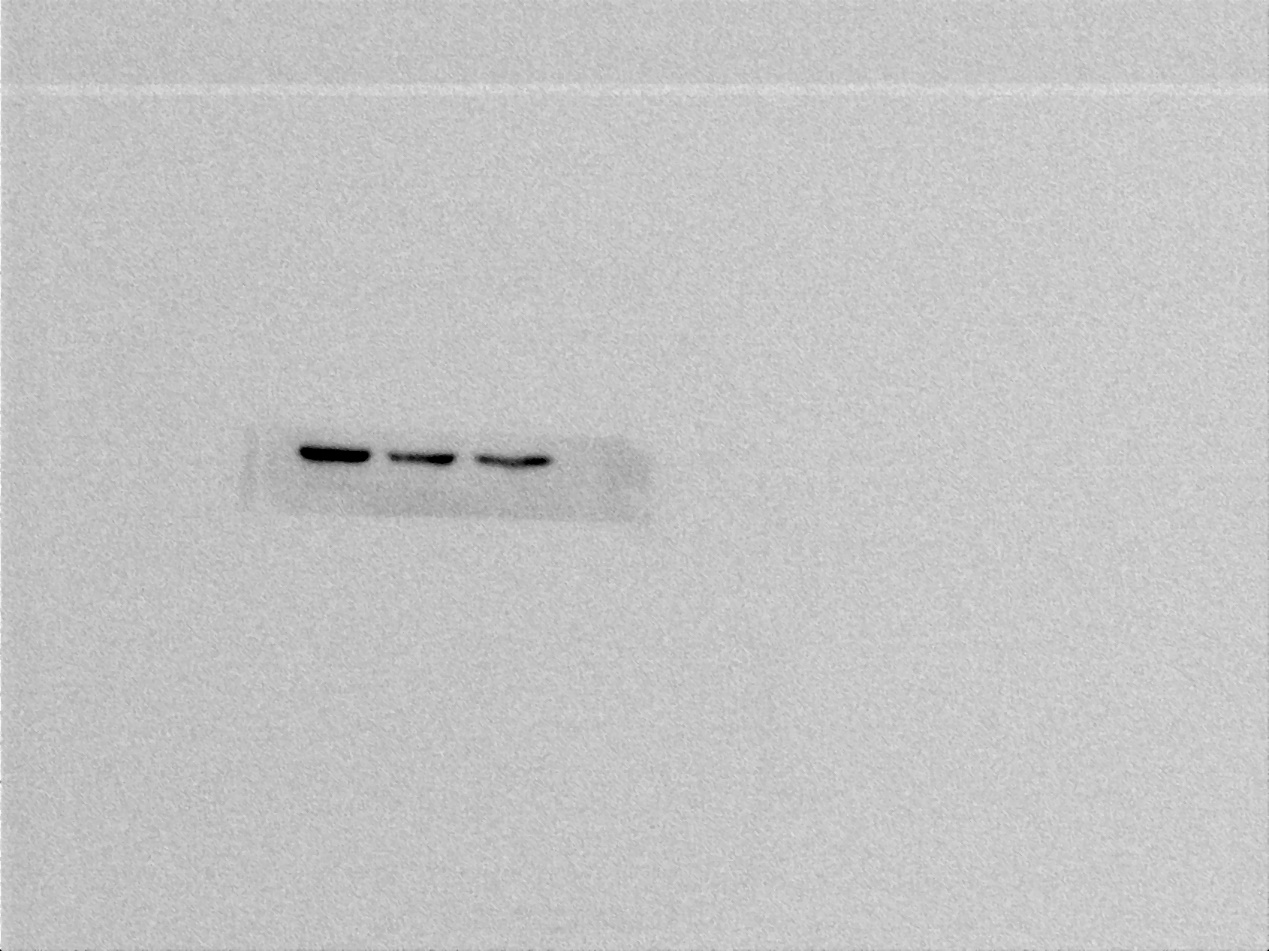


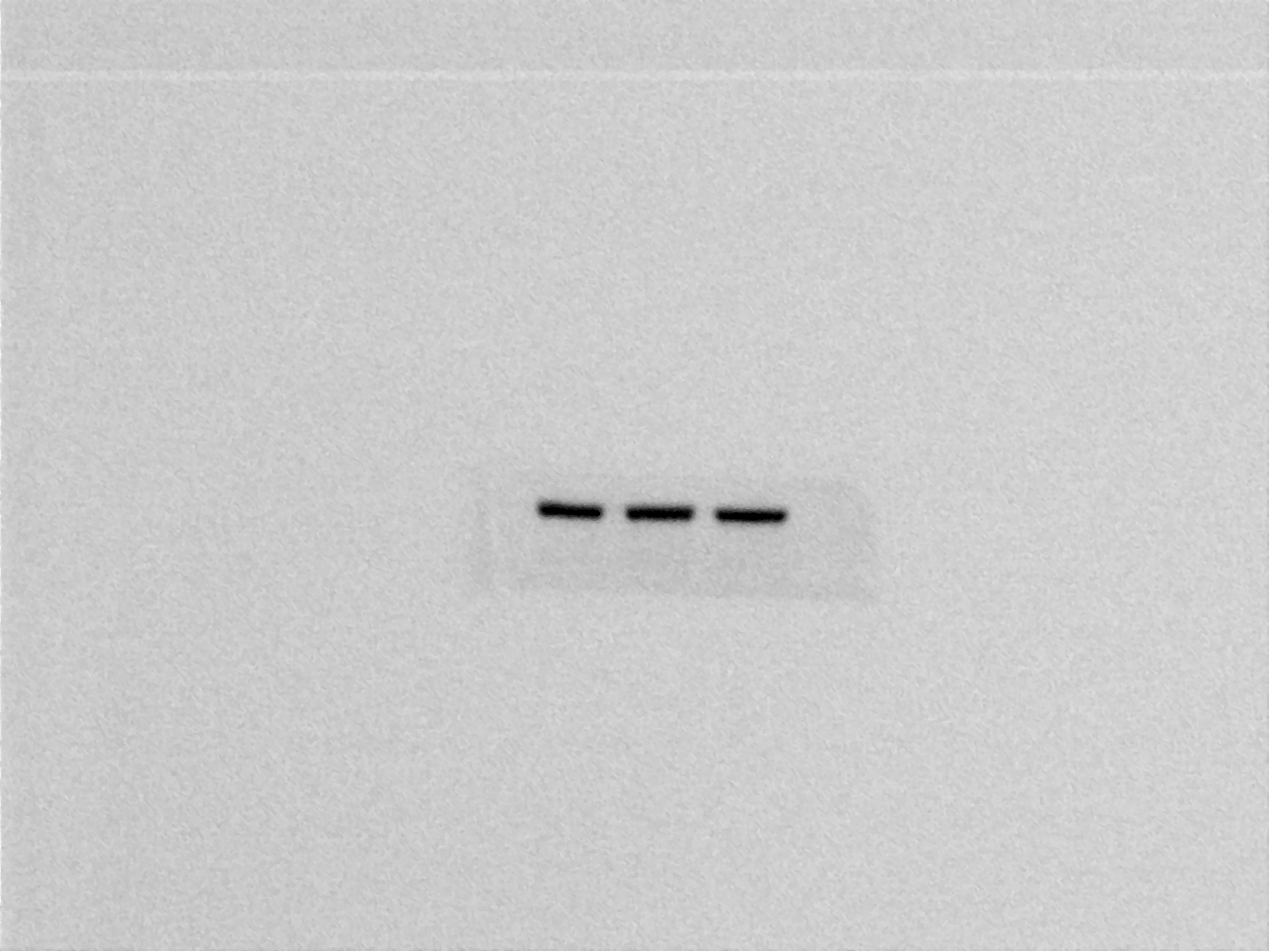

Supplement: Supplementary file 3 — Figure 4 WB Supplementary materials [file 41419_2022_4736_MOESM3_ESM.docx]

Fig 5B


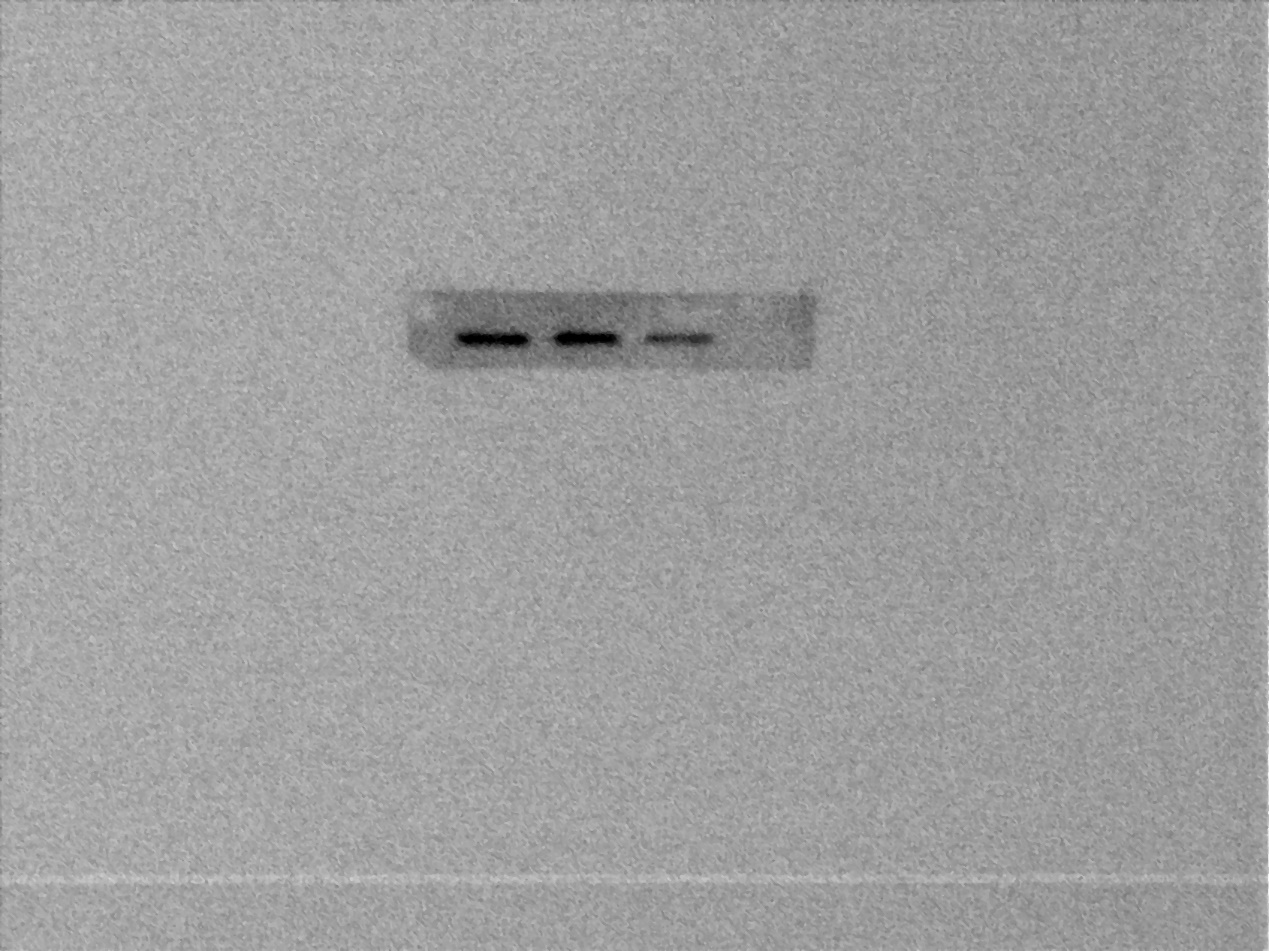


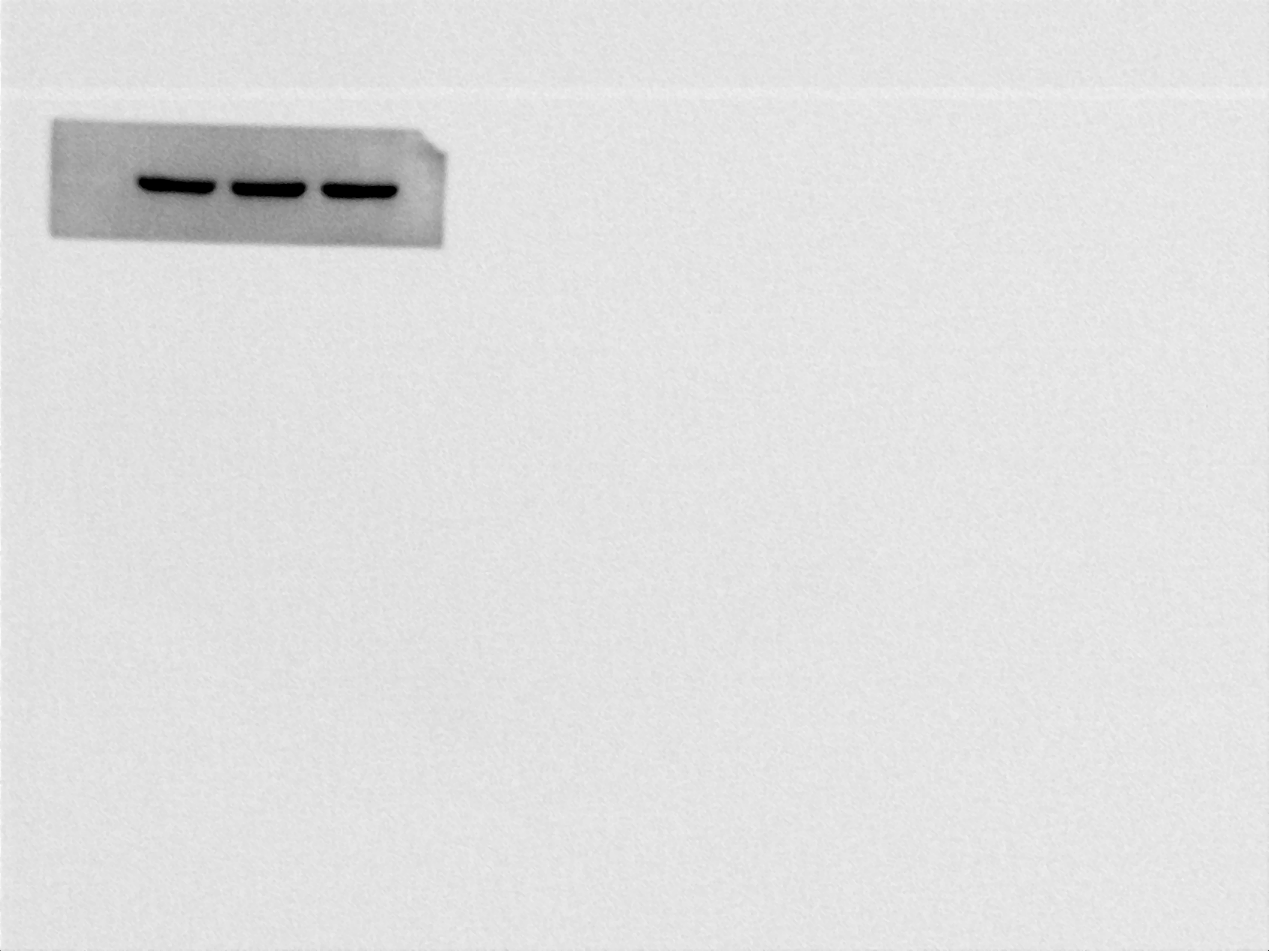


Fig 5E


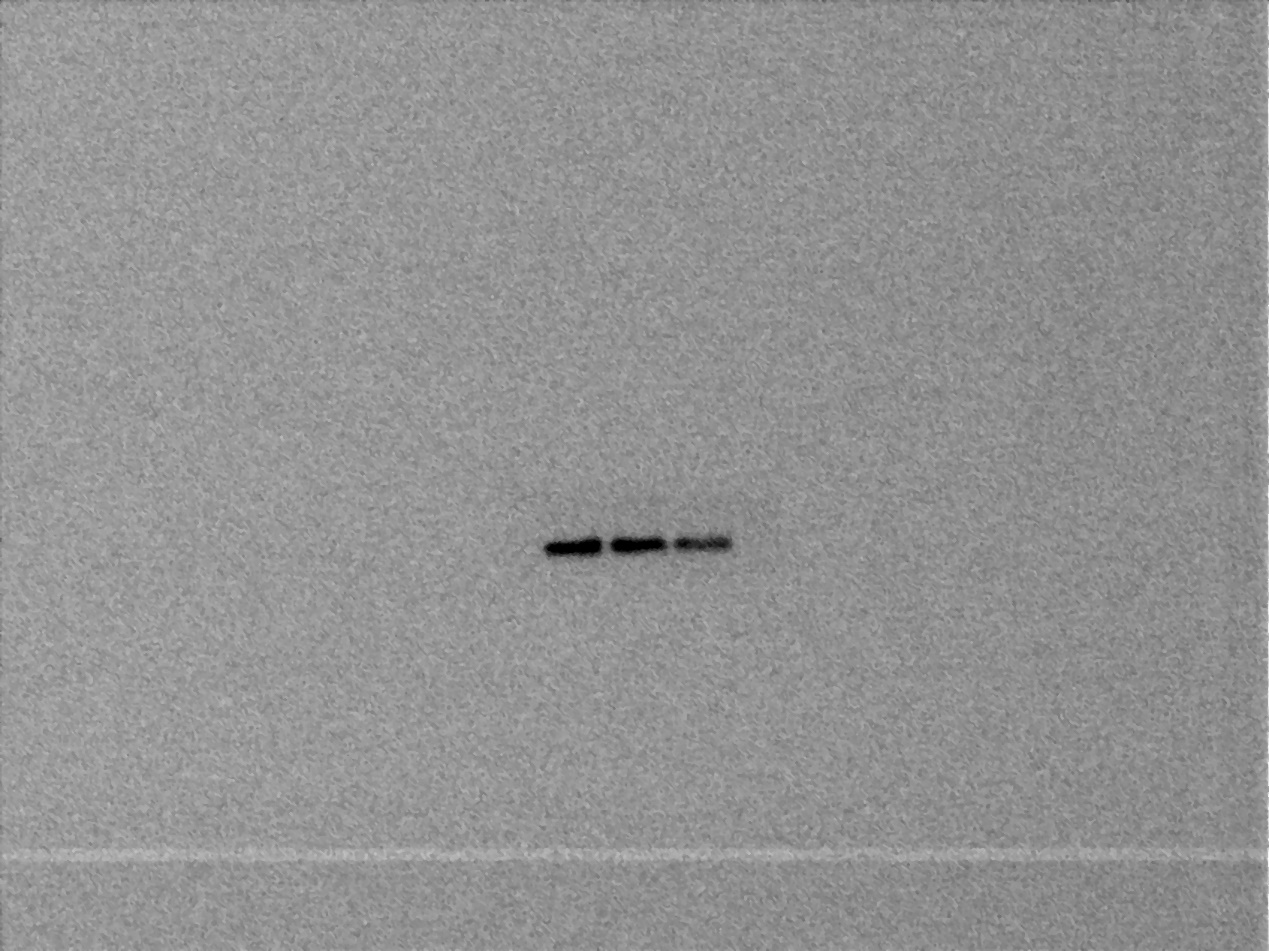


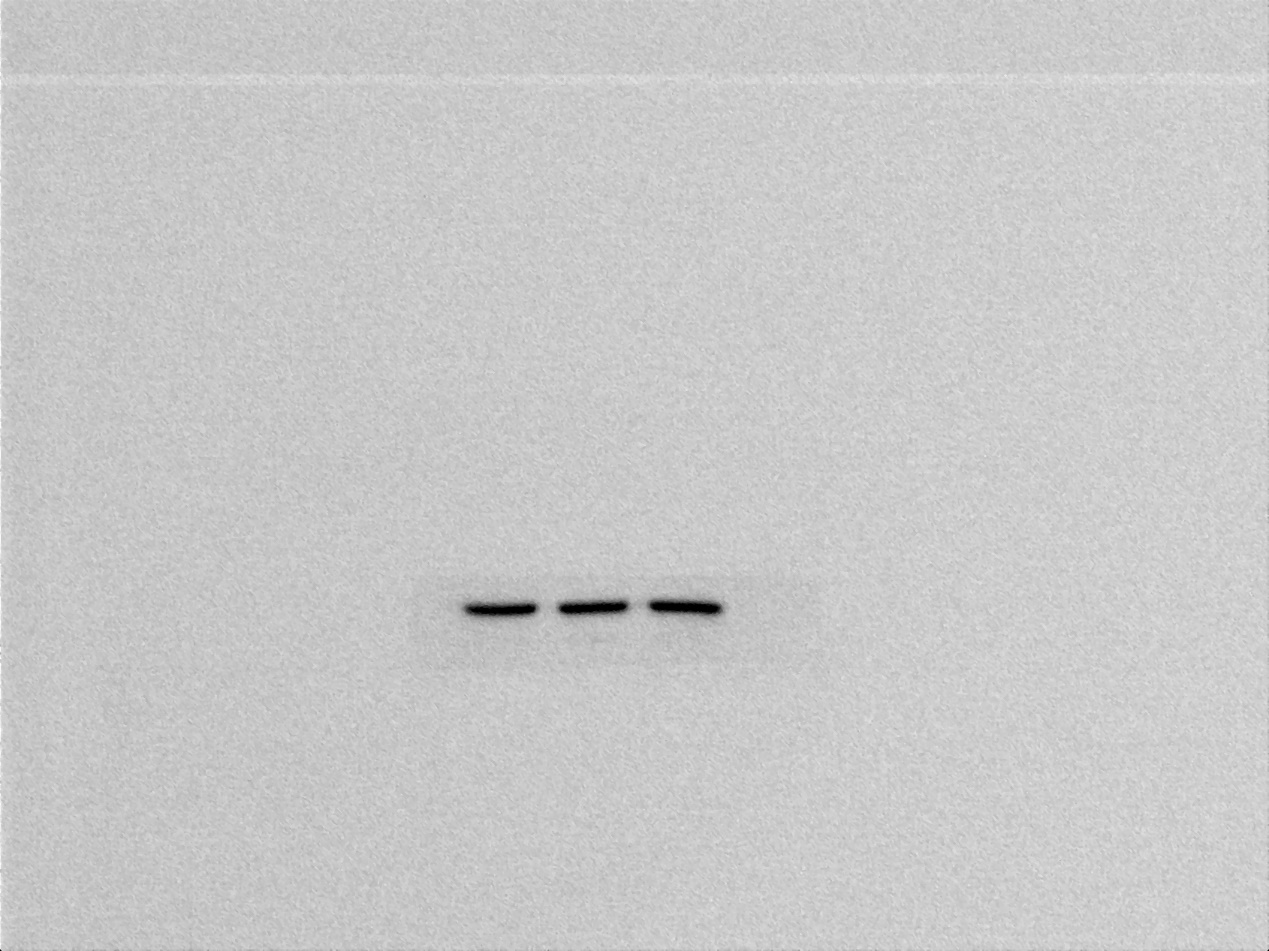


Fig 5G


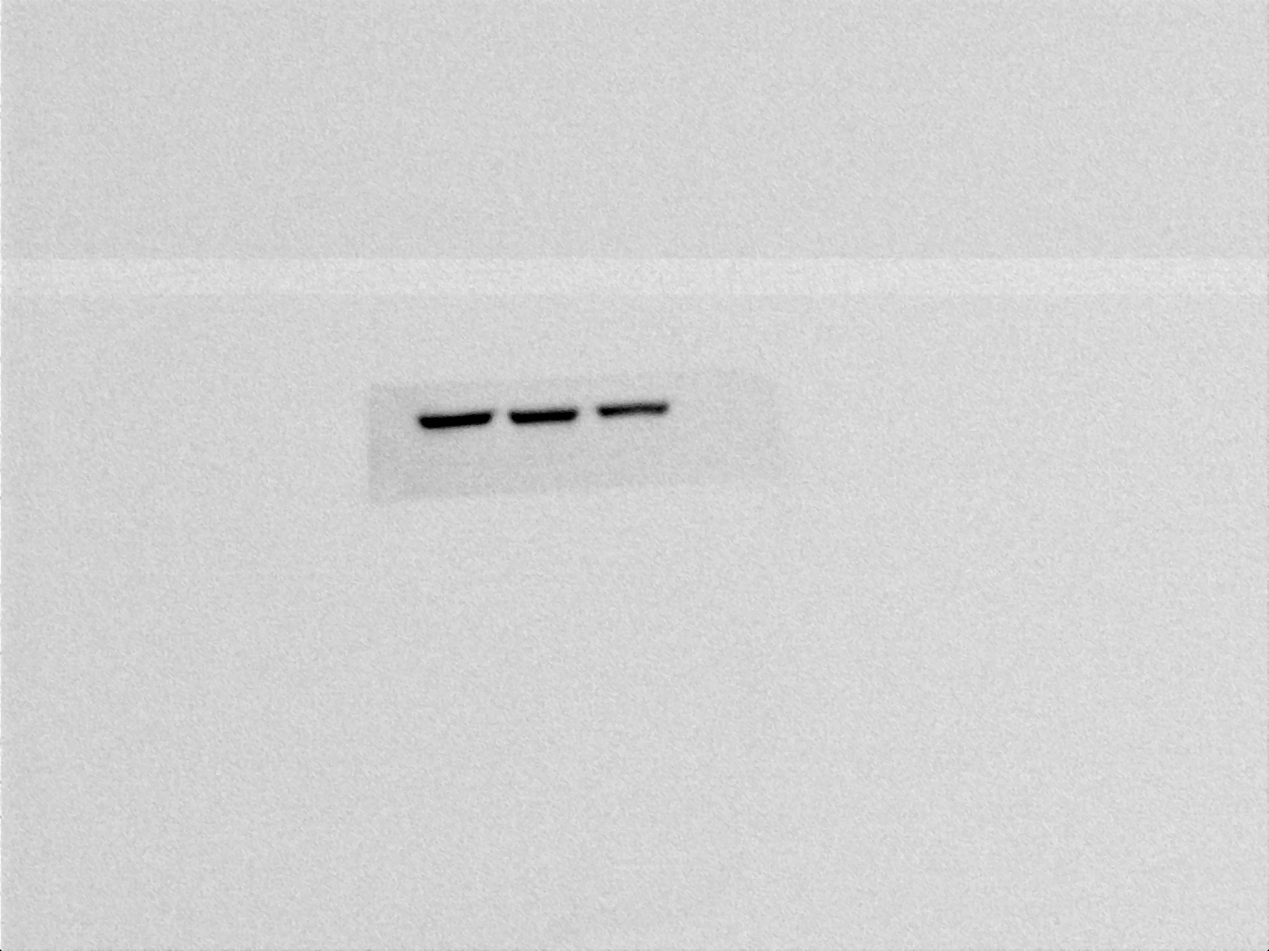


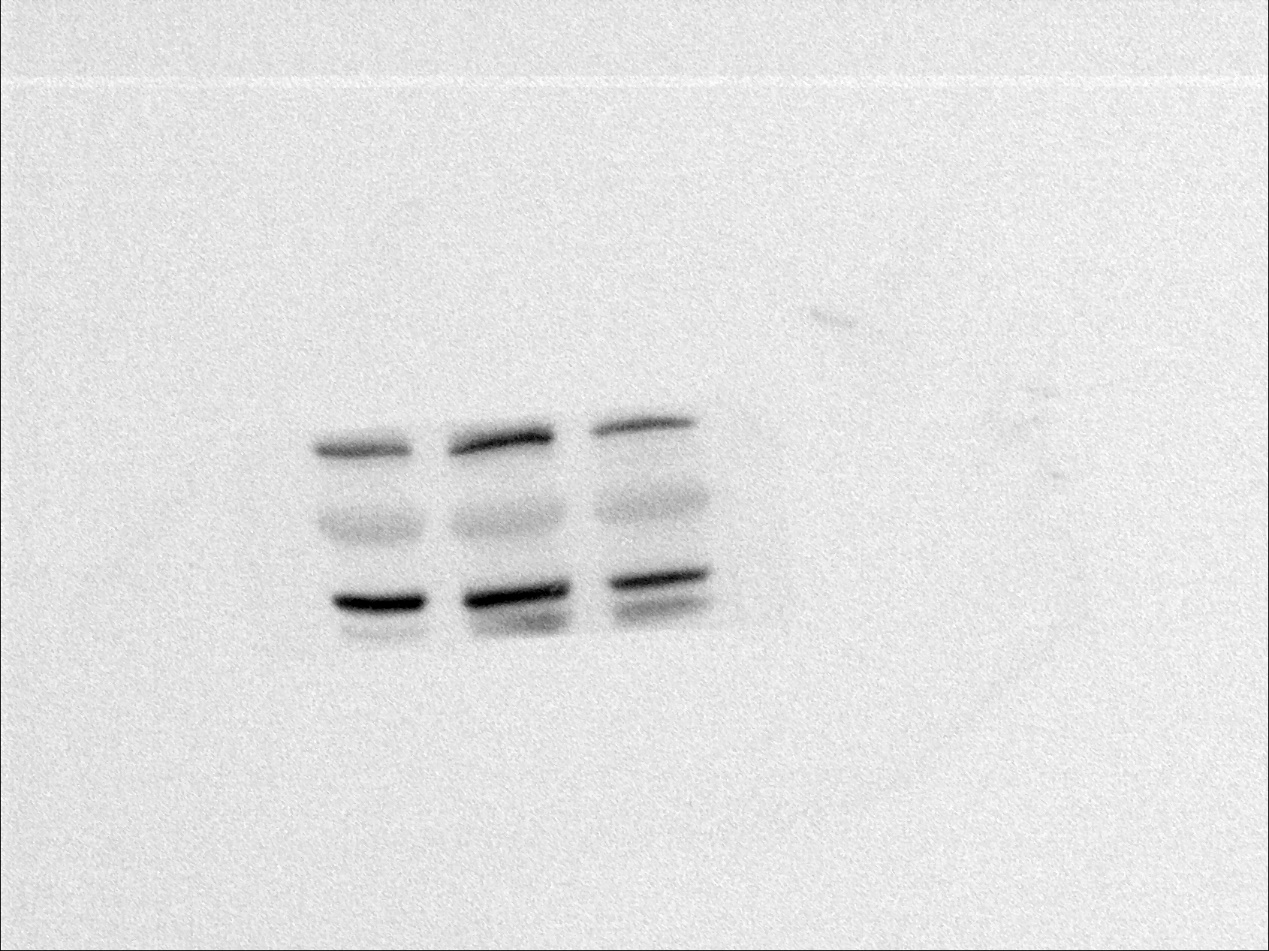


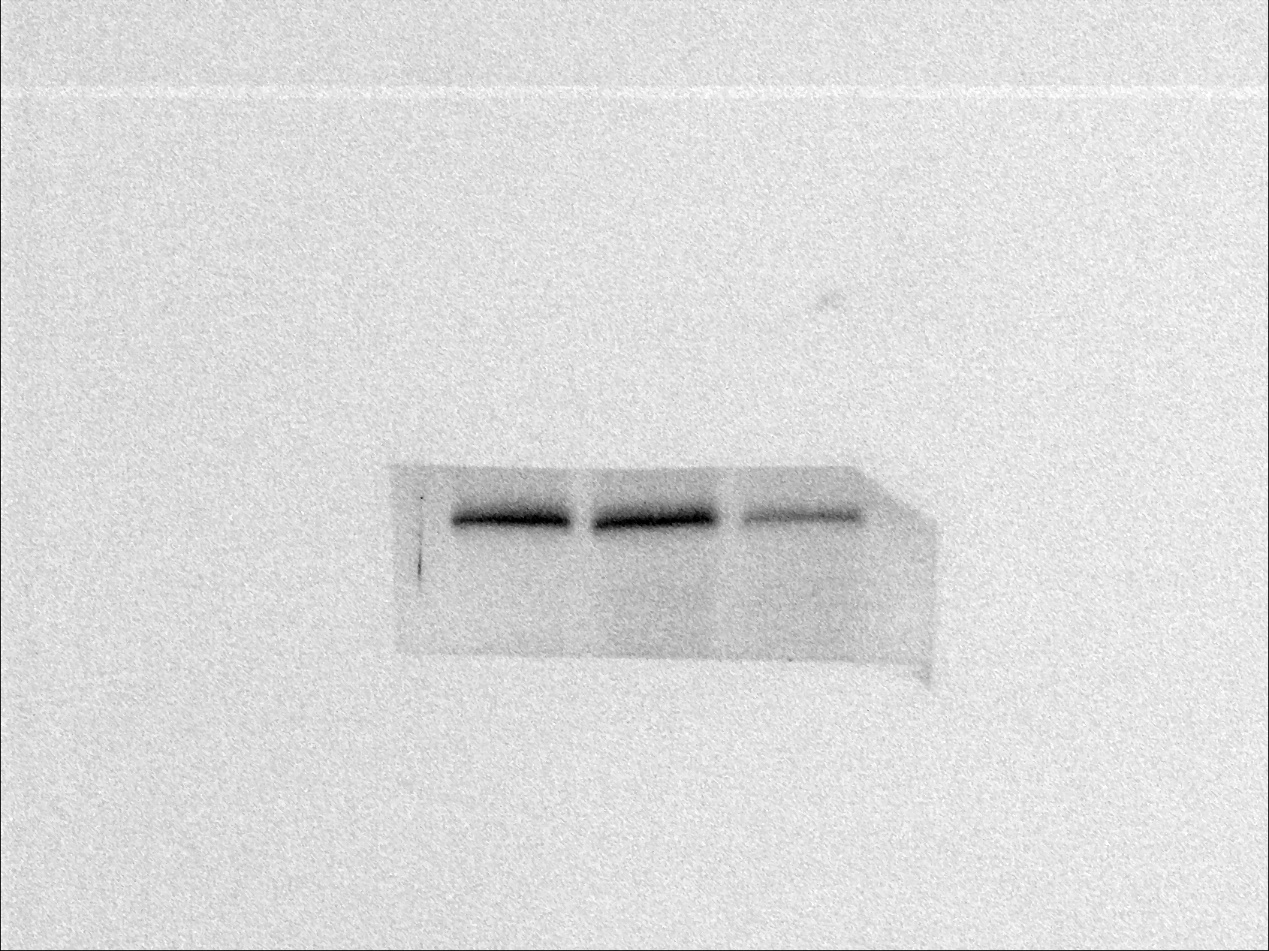


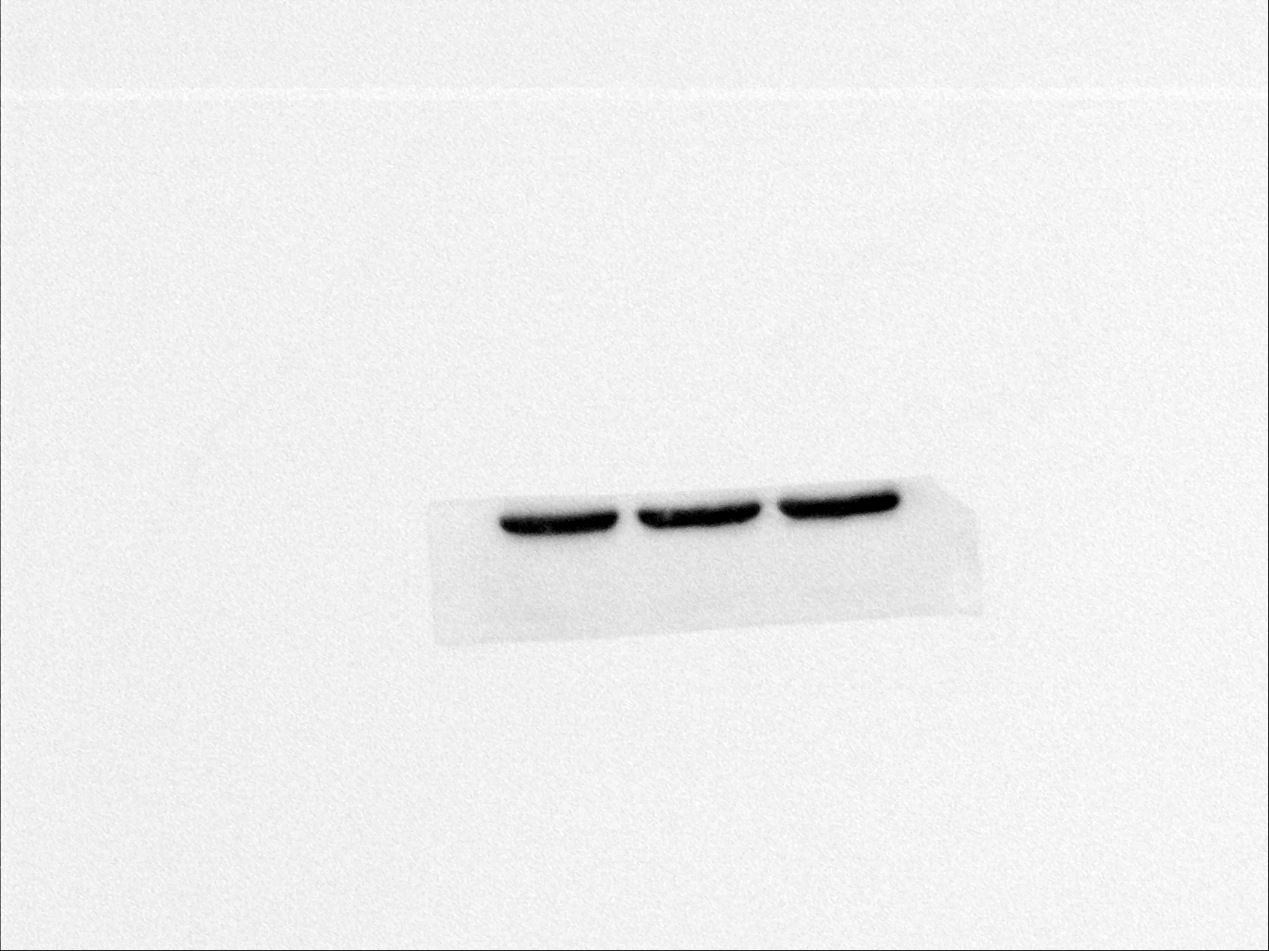


Fig 5I


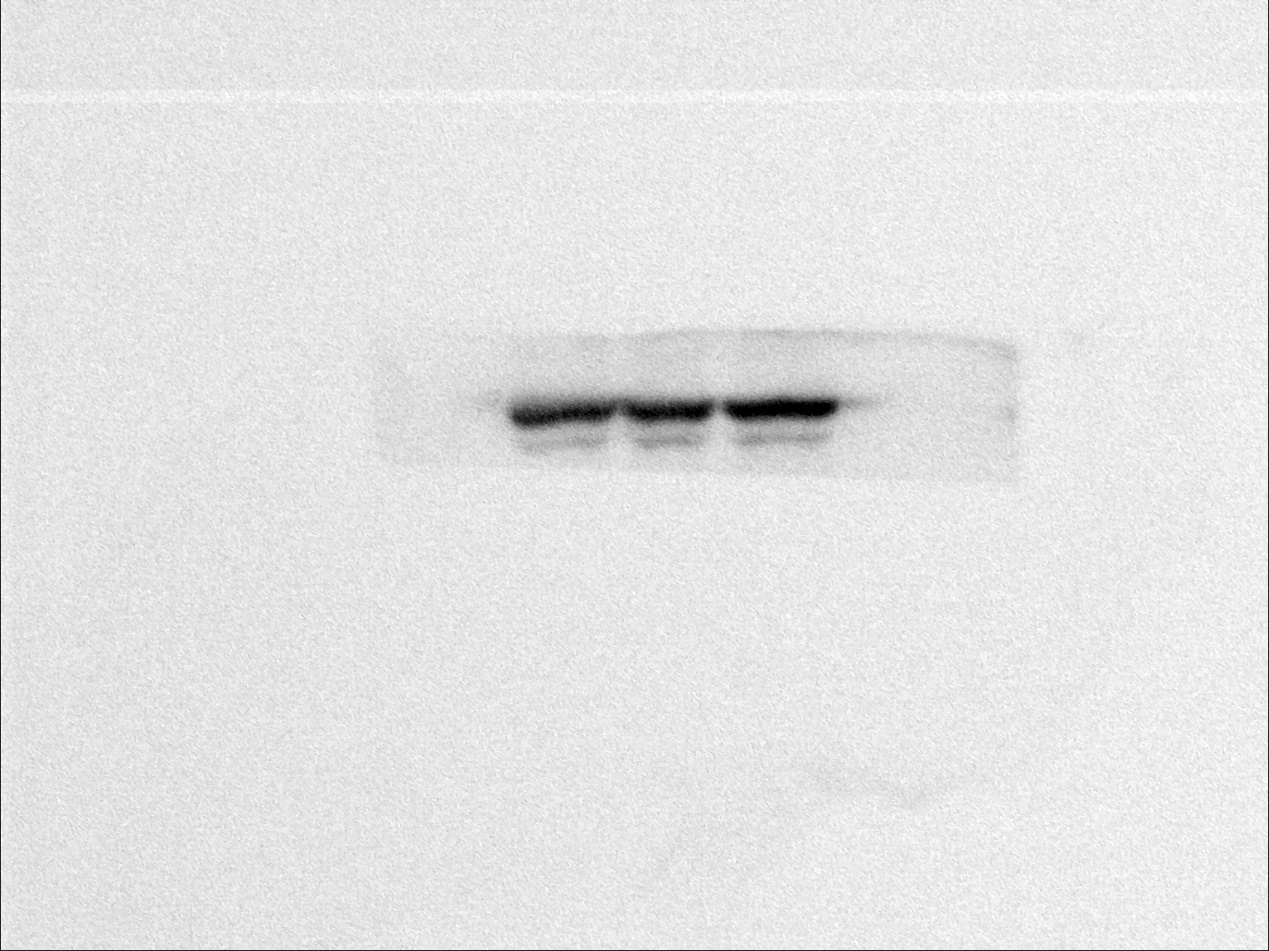


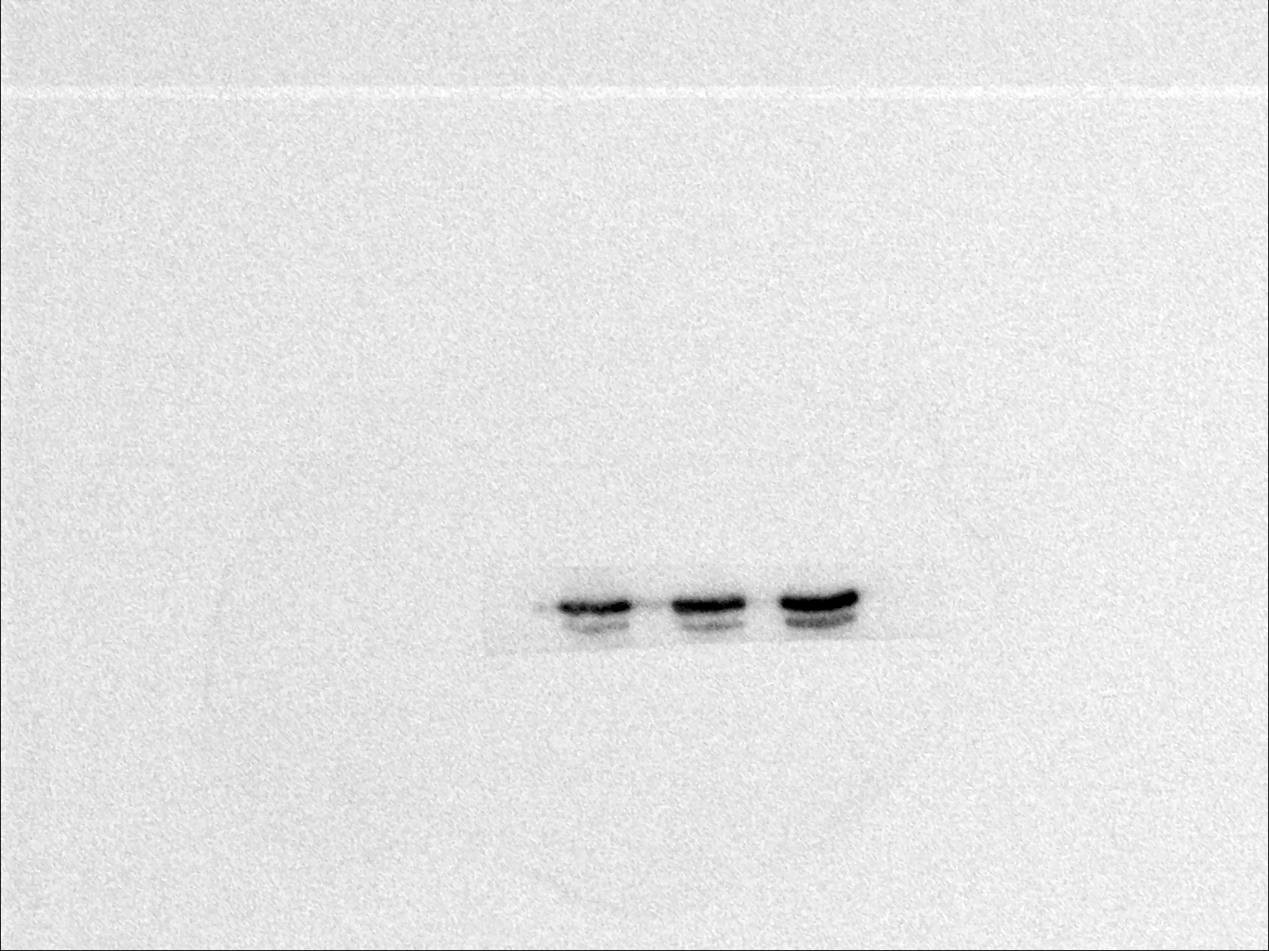


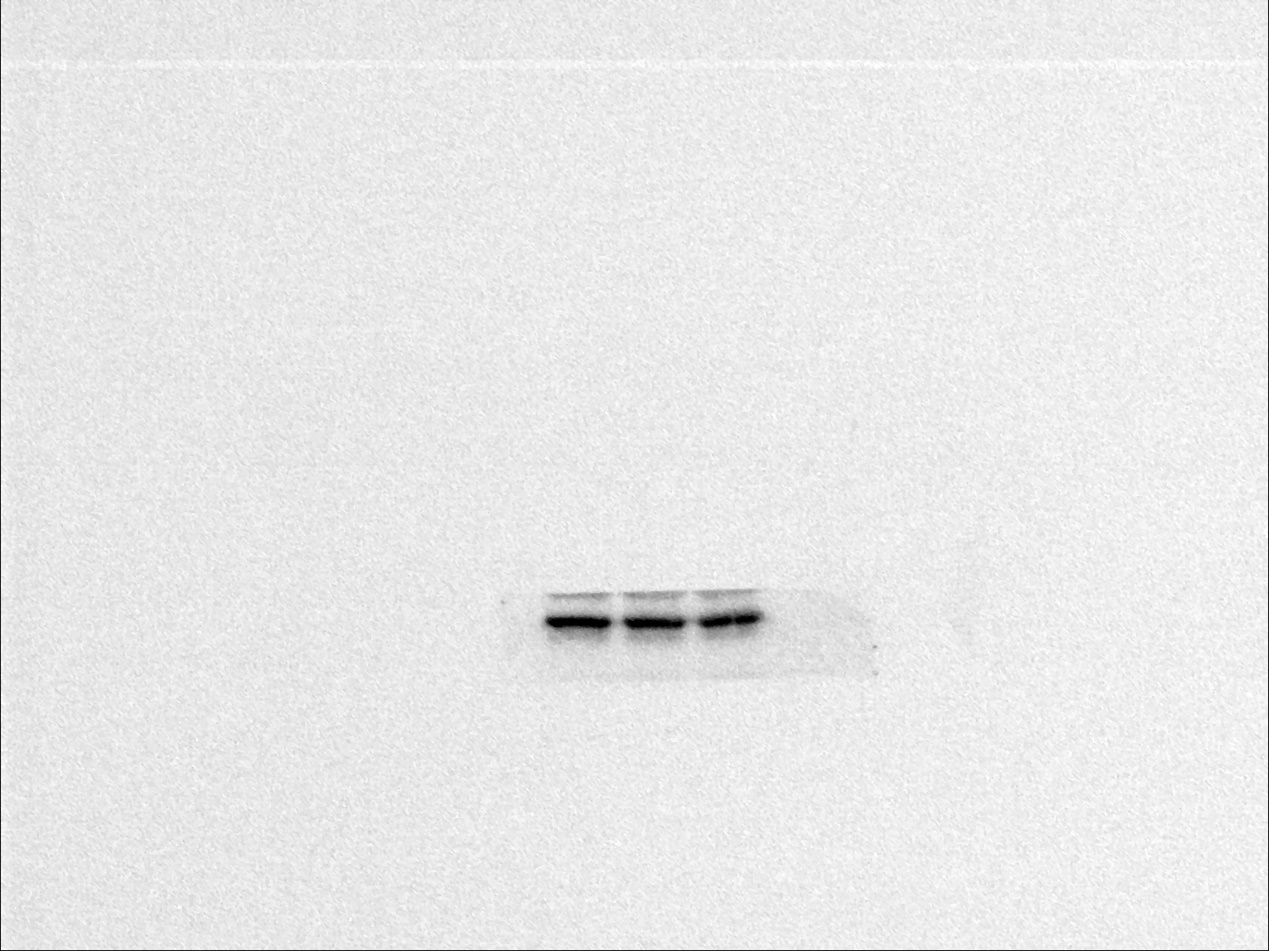


Fig 5K


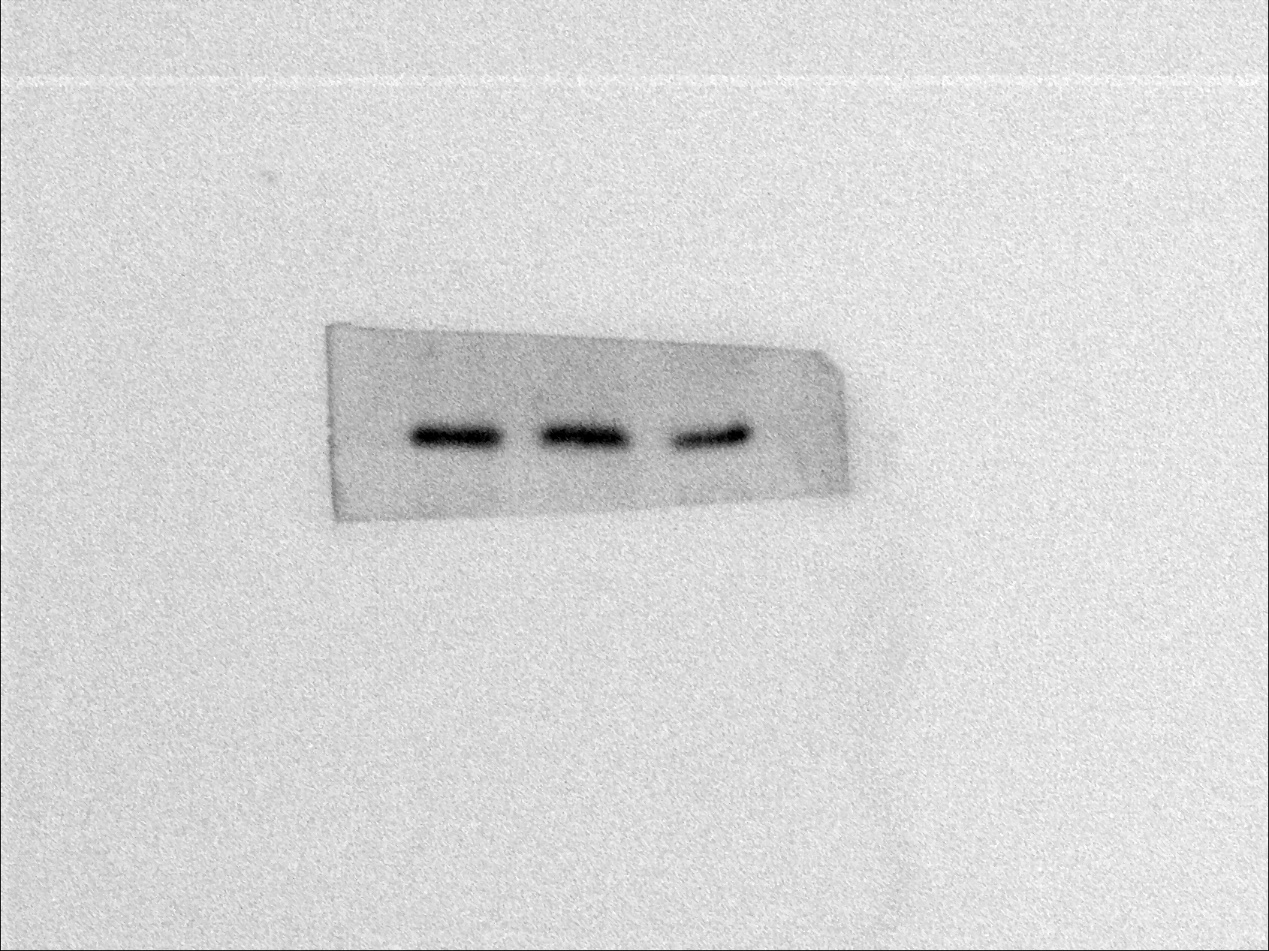


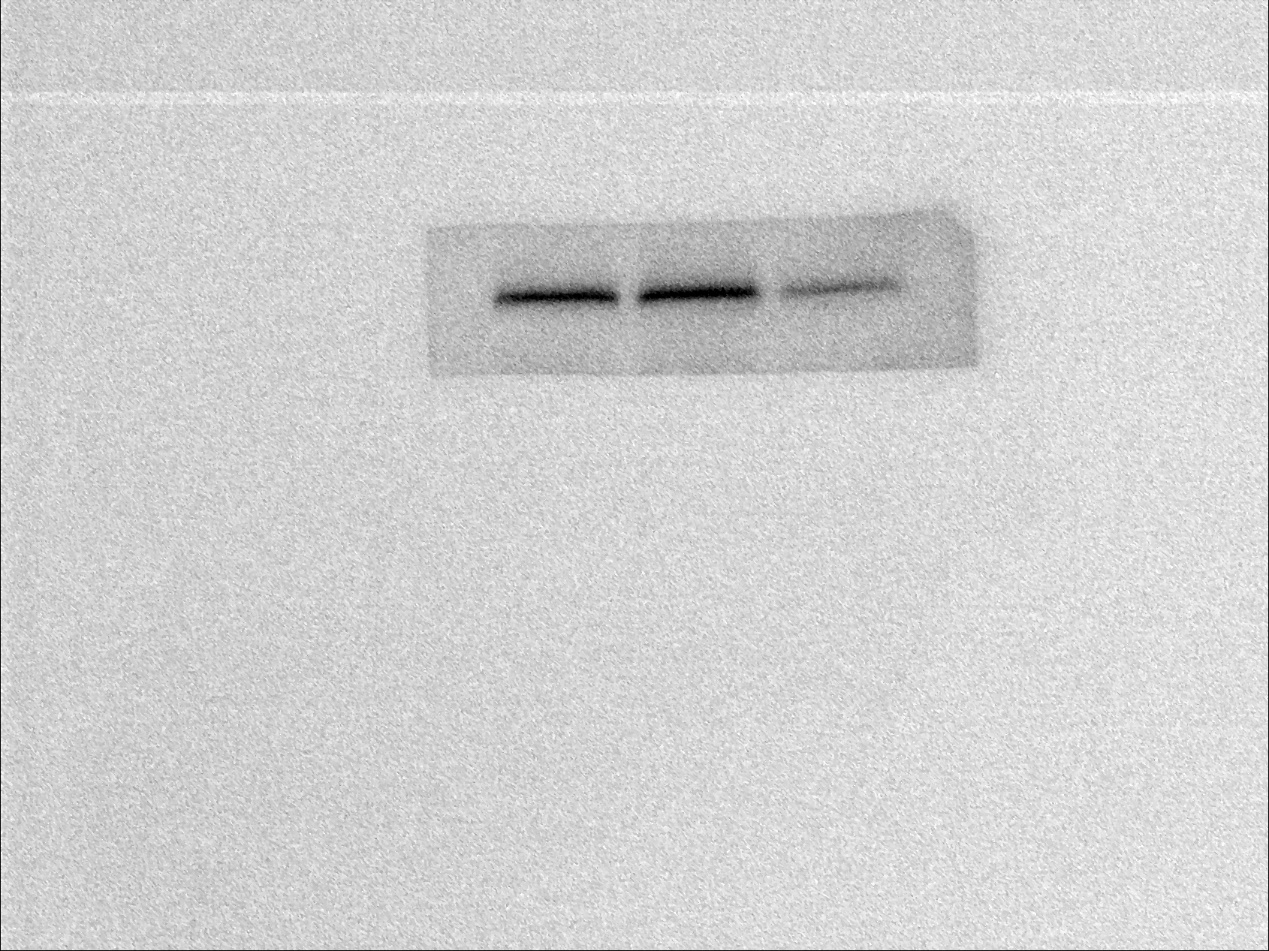


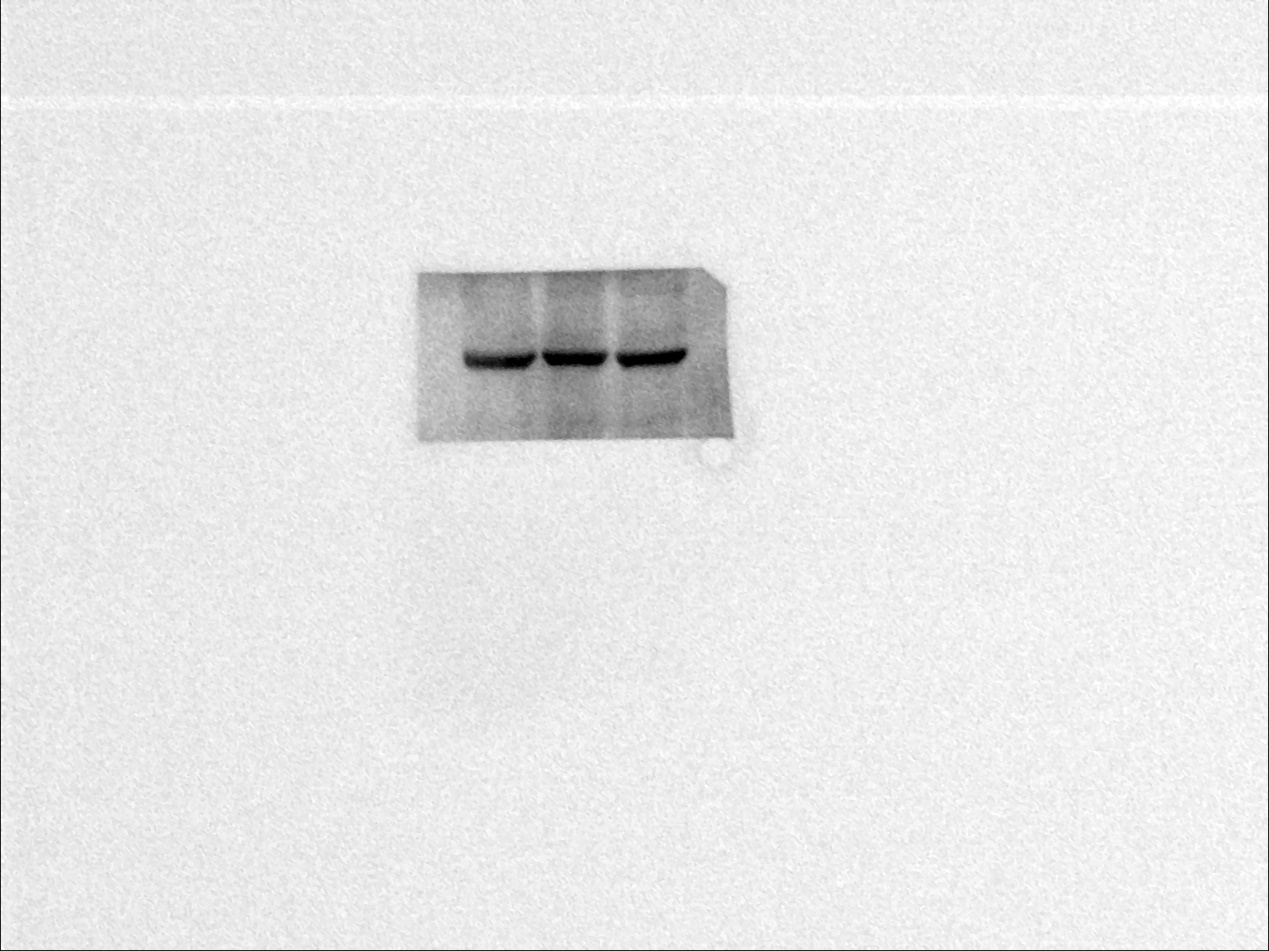

Supplement: Supplementary file 4 — Figure 5 WB Supplementary materials [file 41419_2022_4736_MOESM4_ESM.docx]

Fig 6I


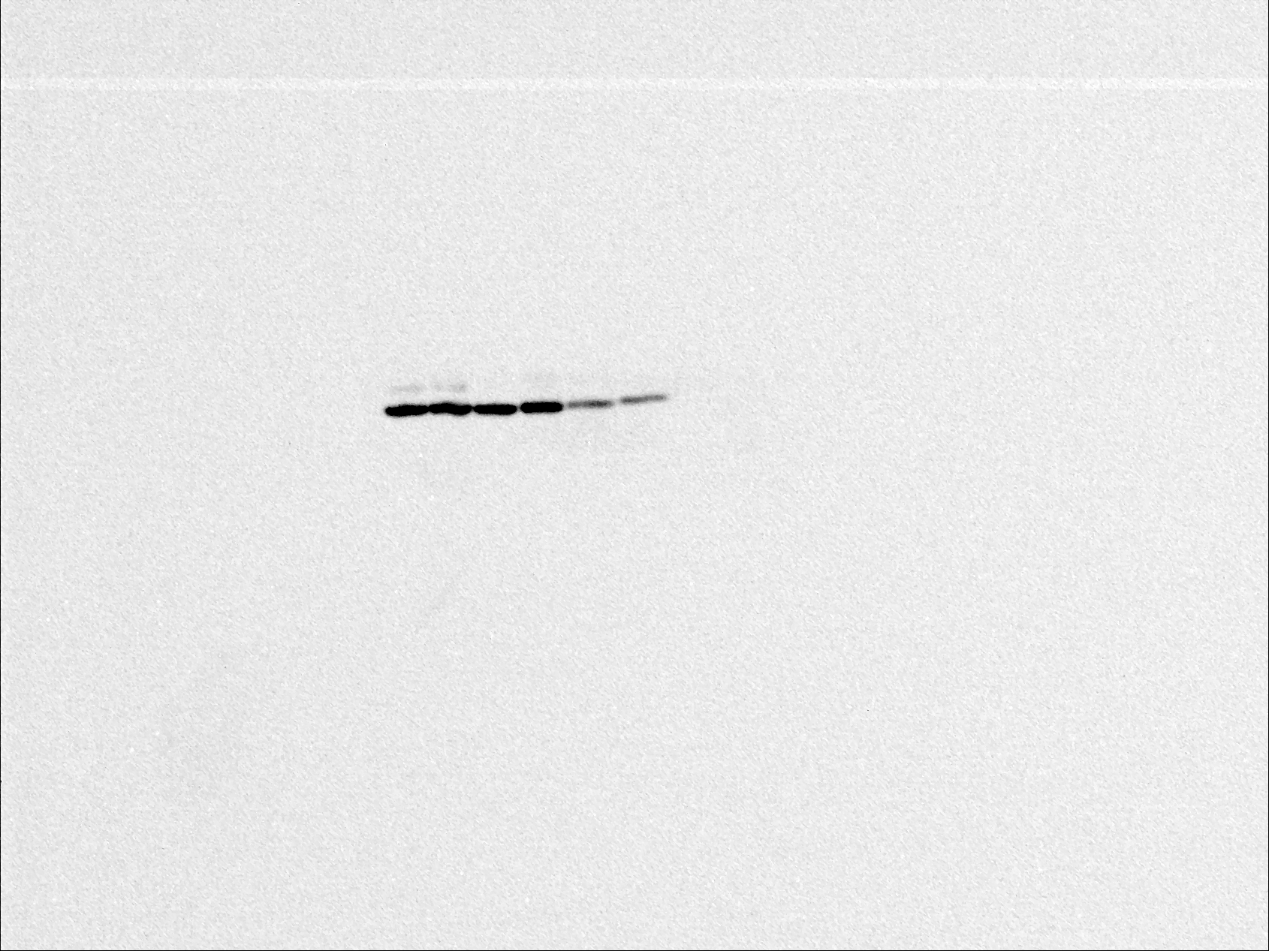


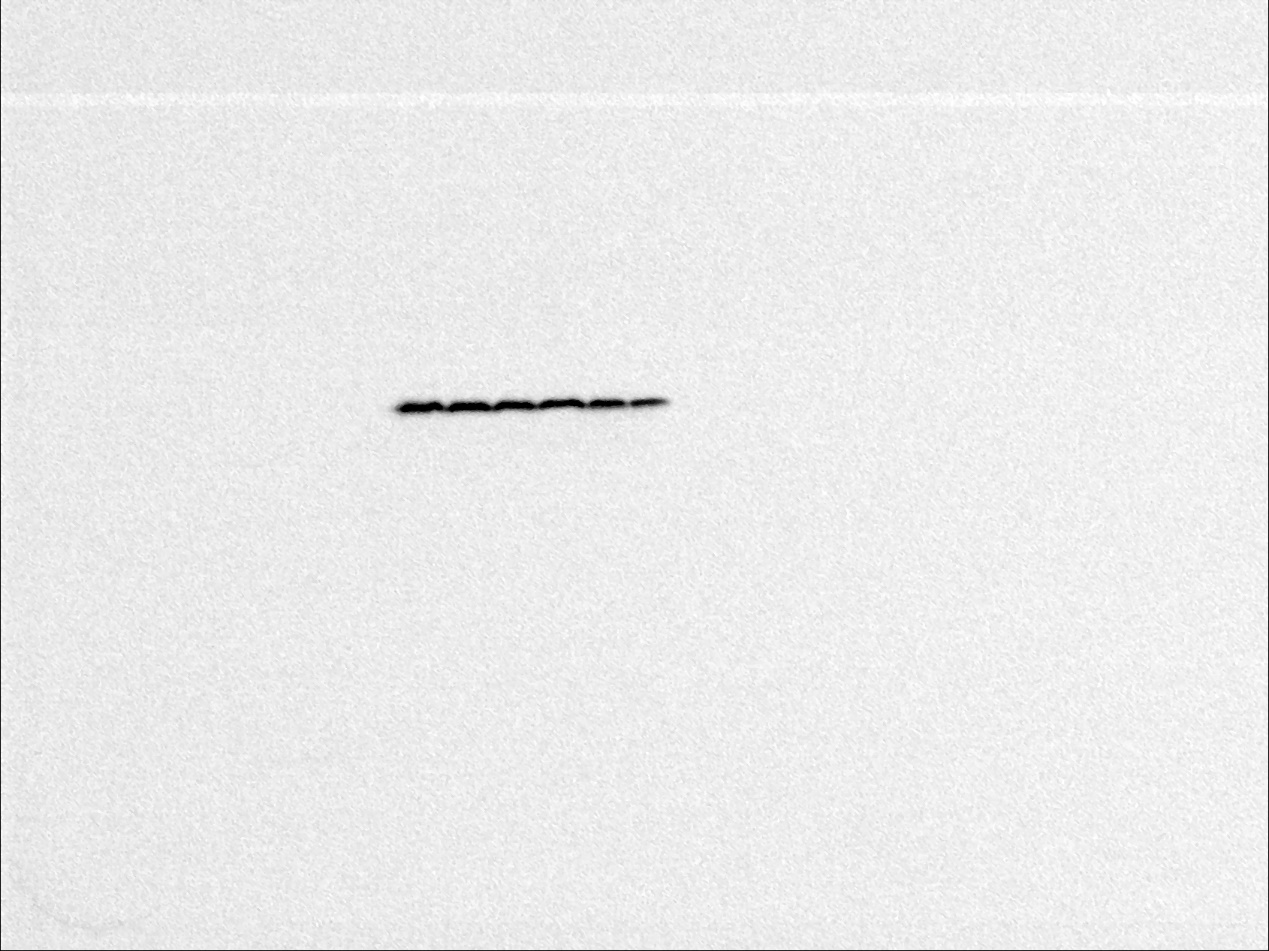


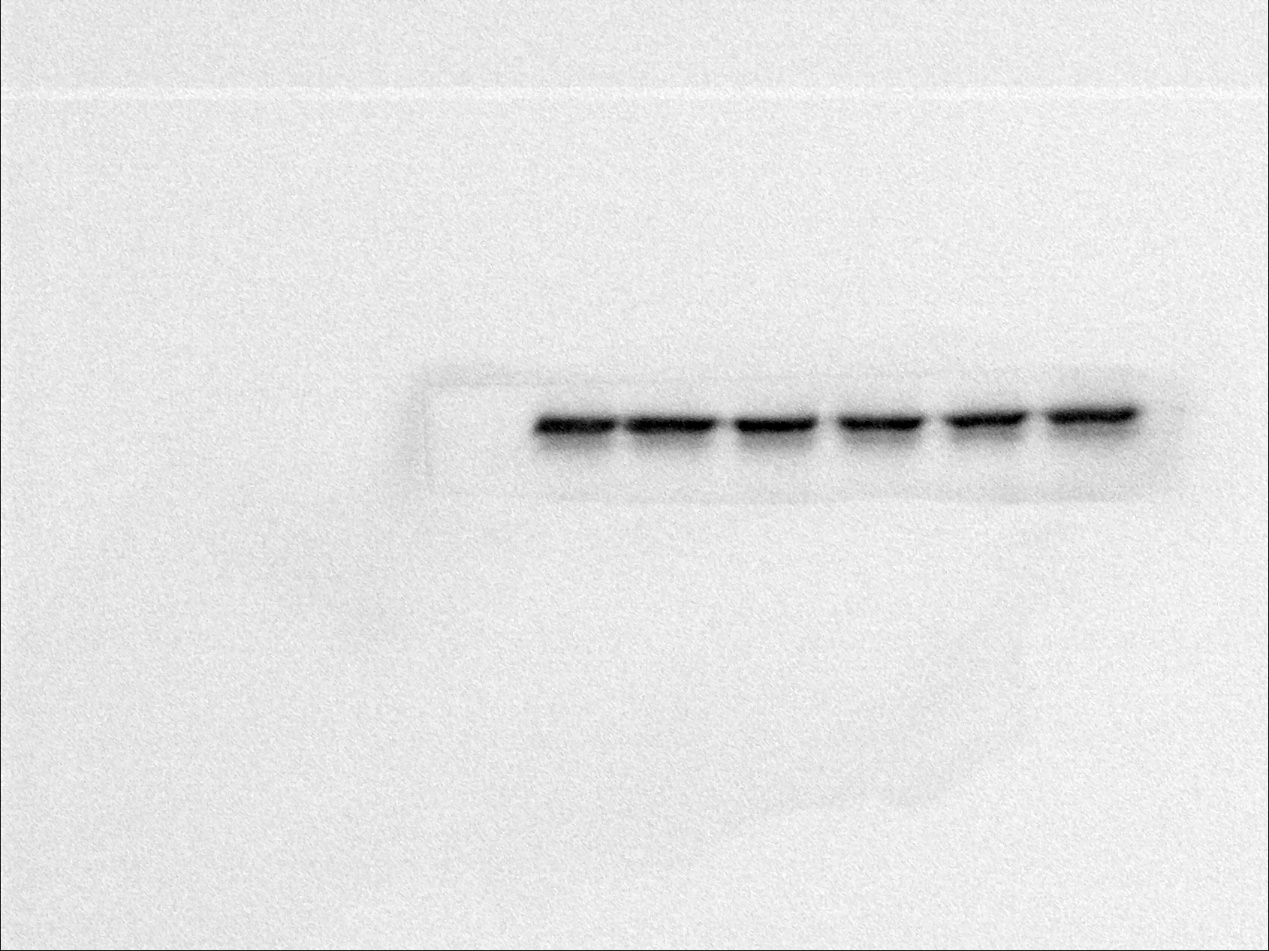


Fig 6K


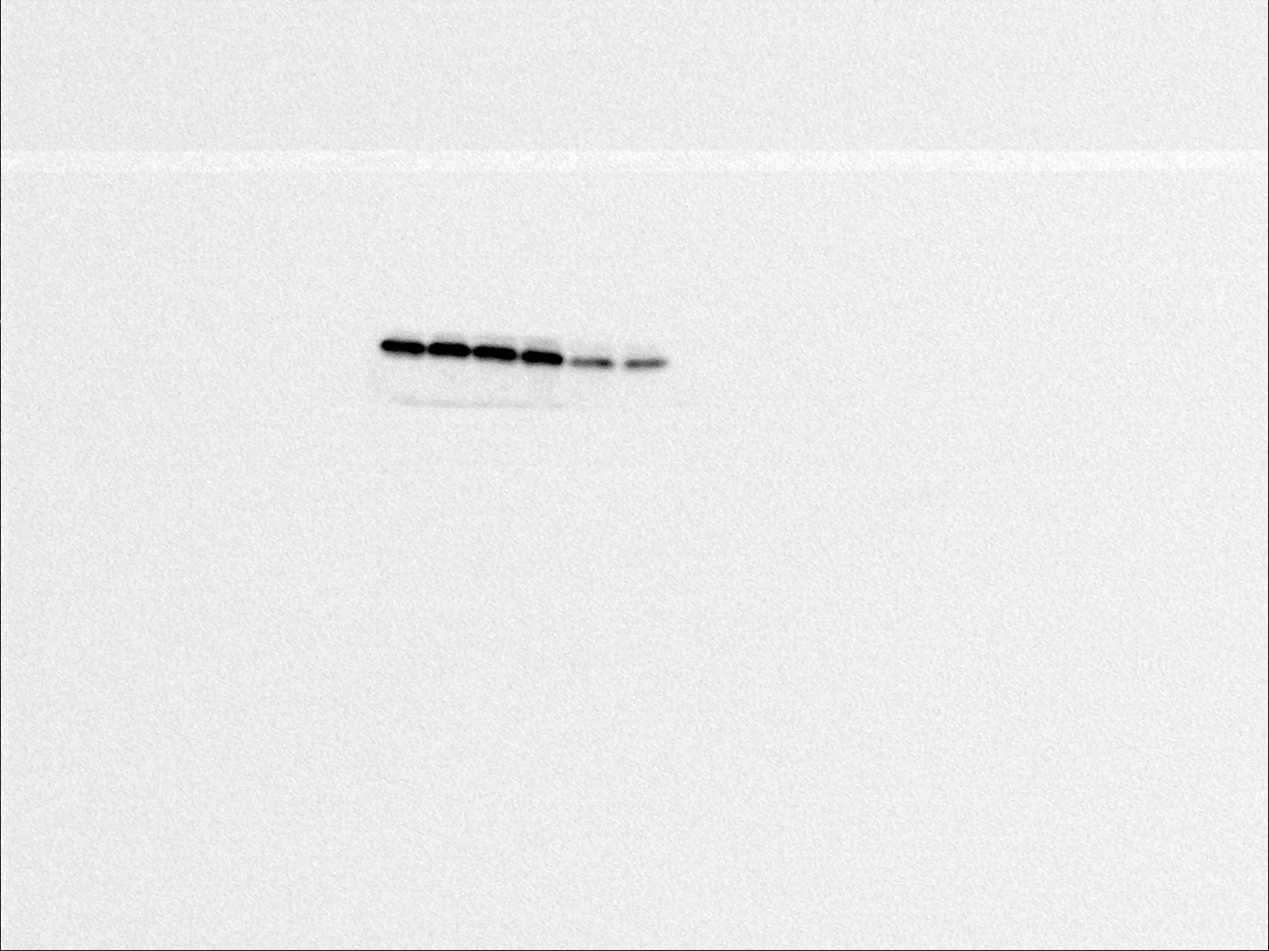


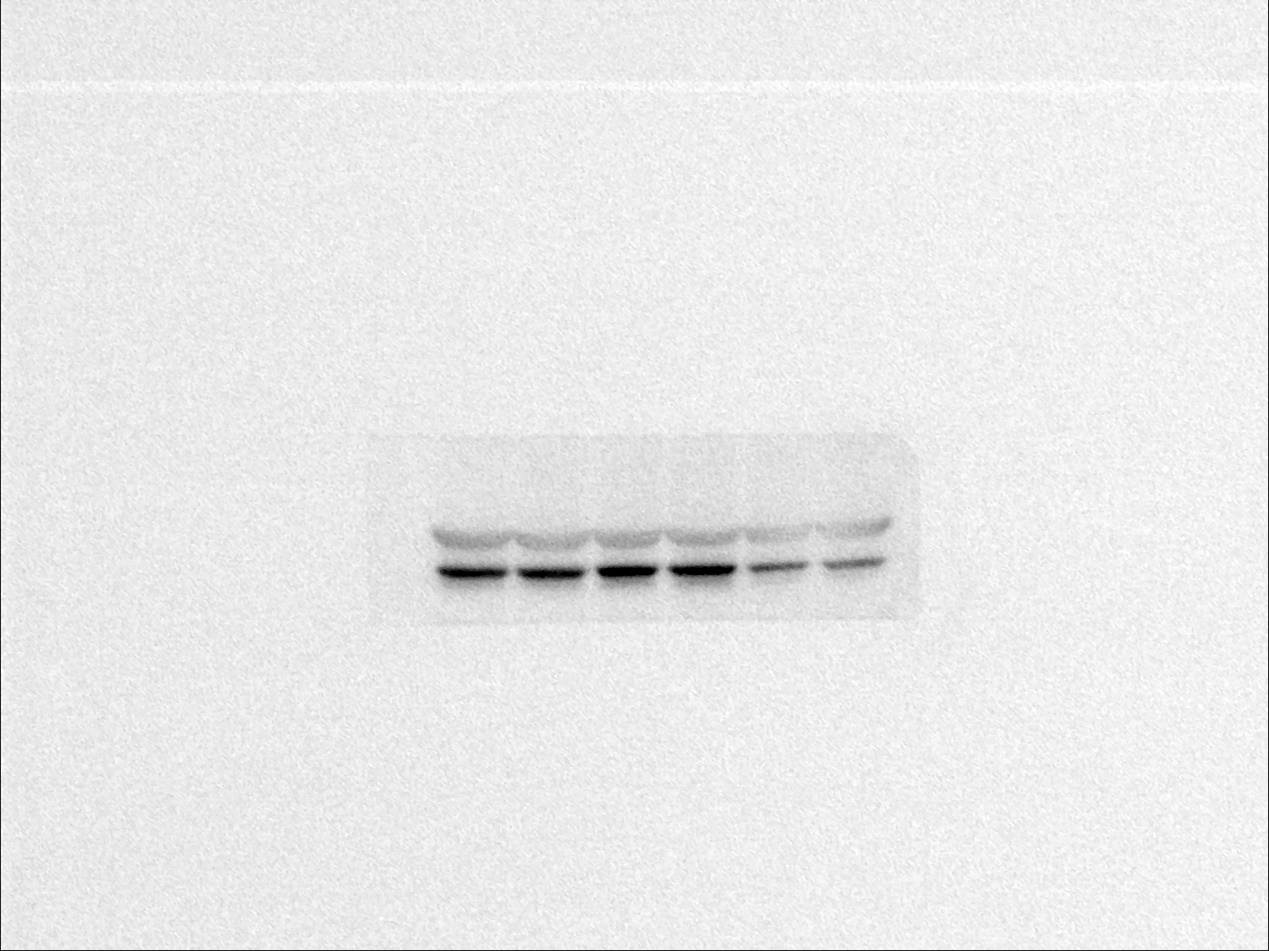


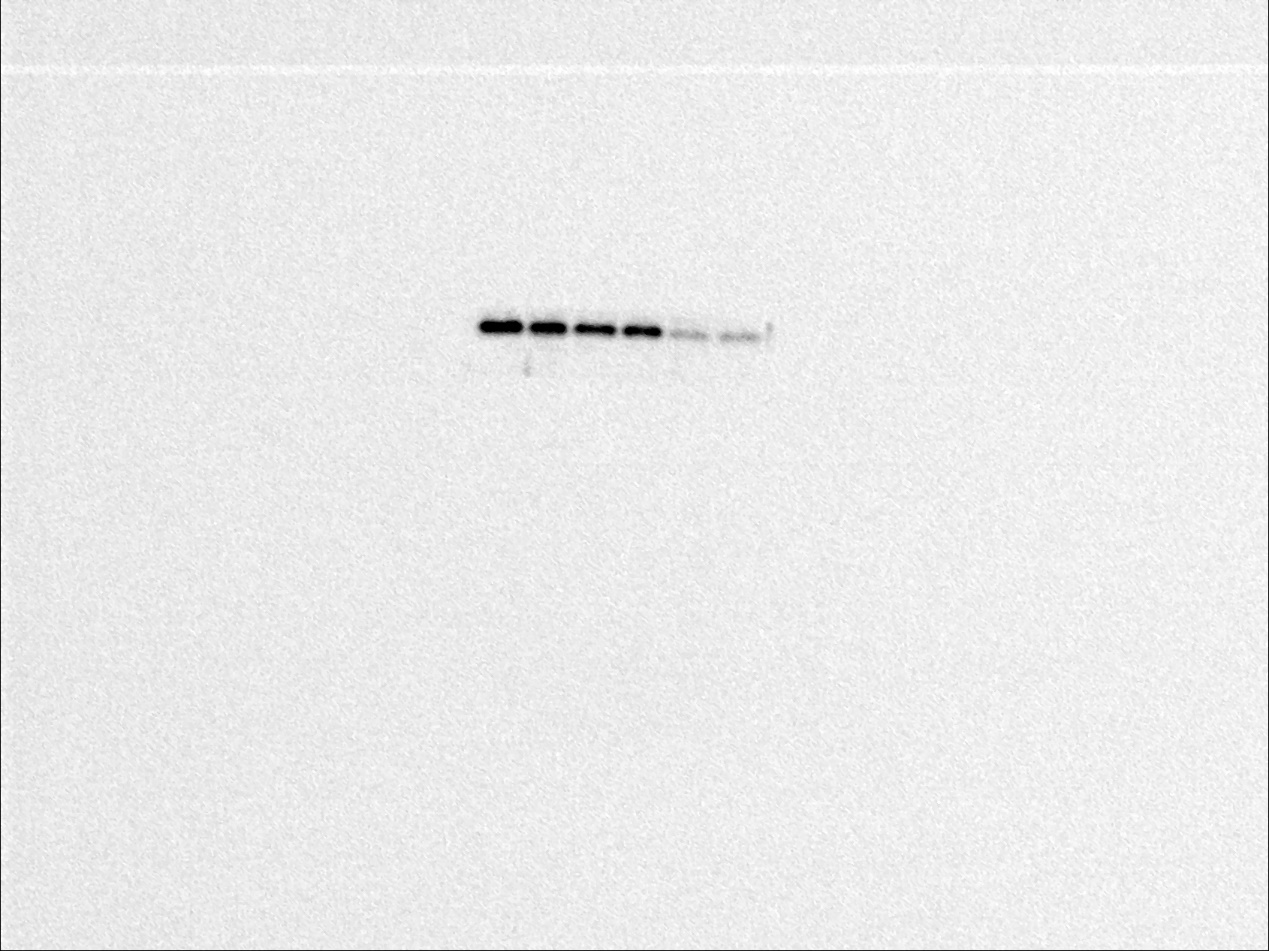


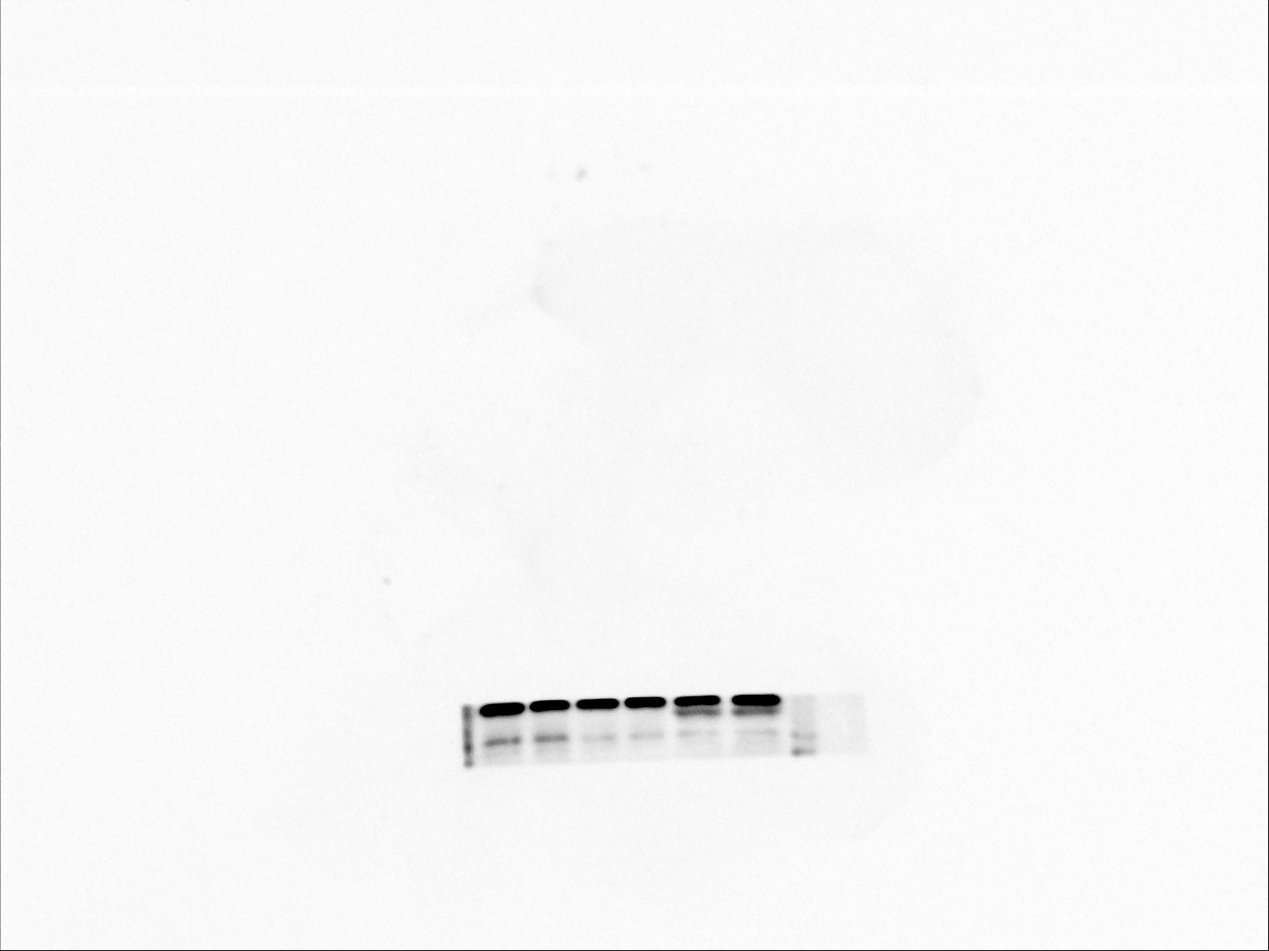


Fig 6M


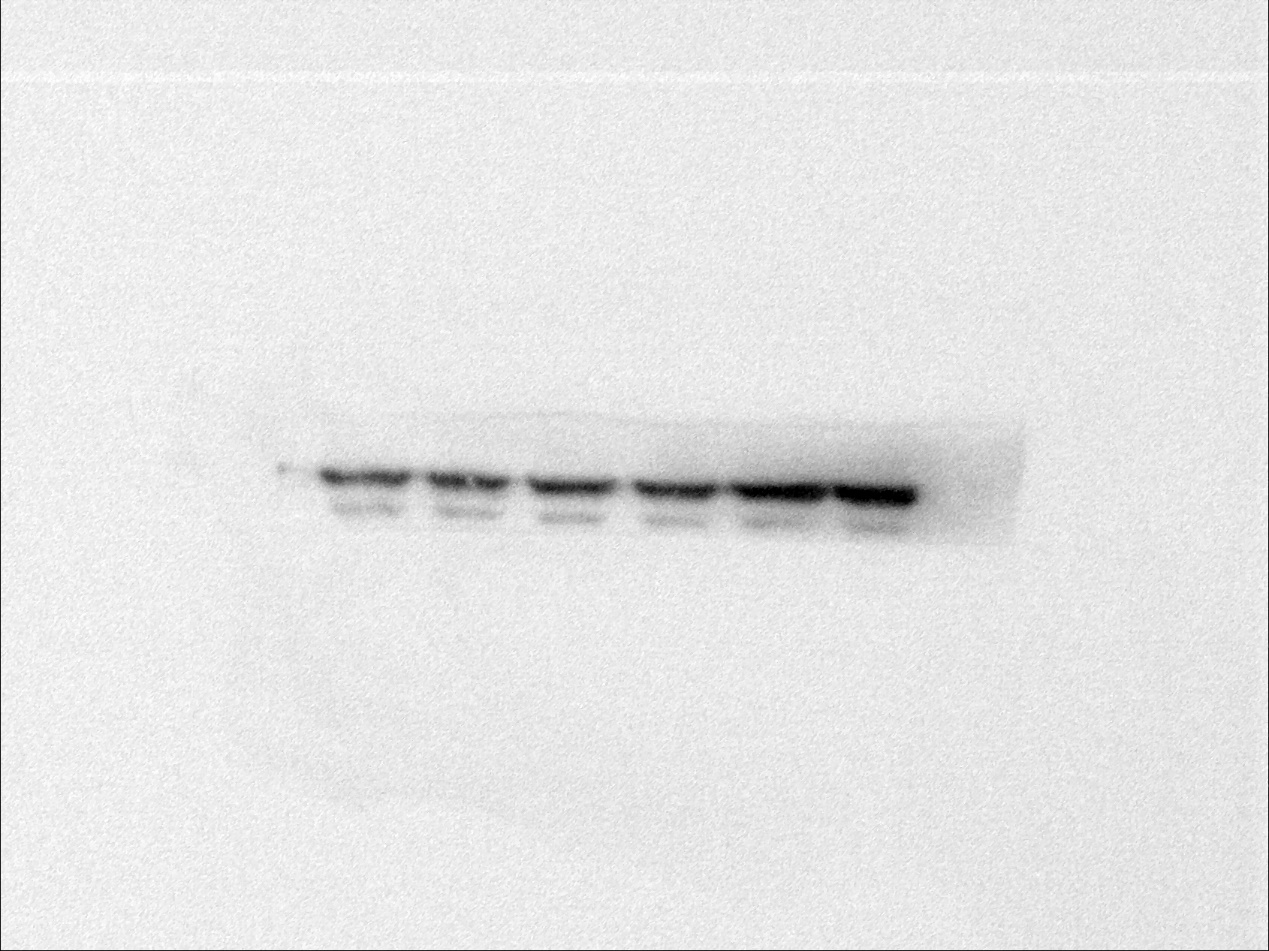


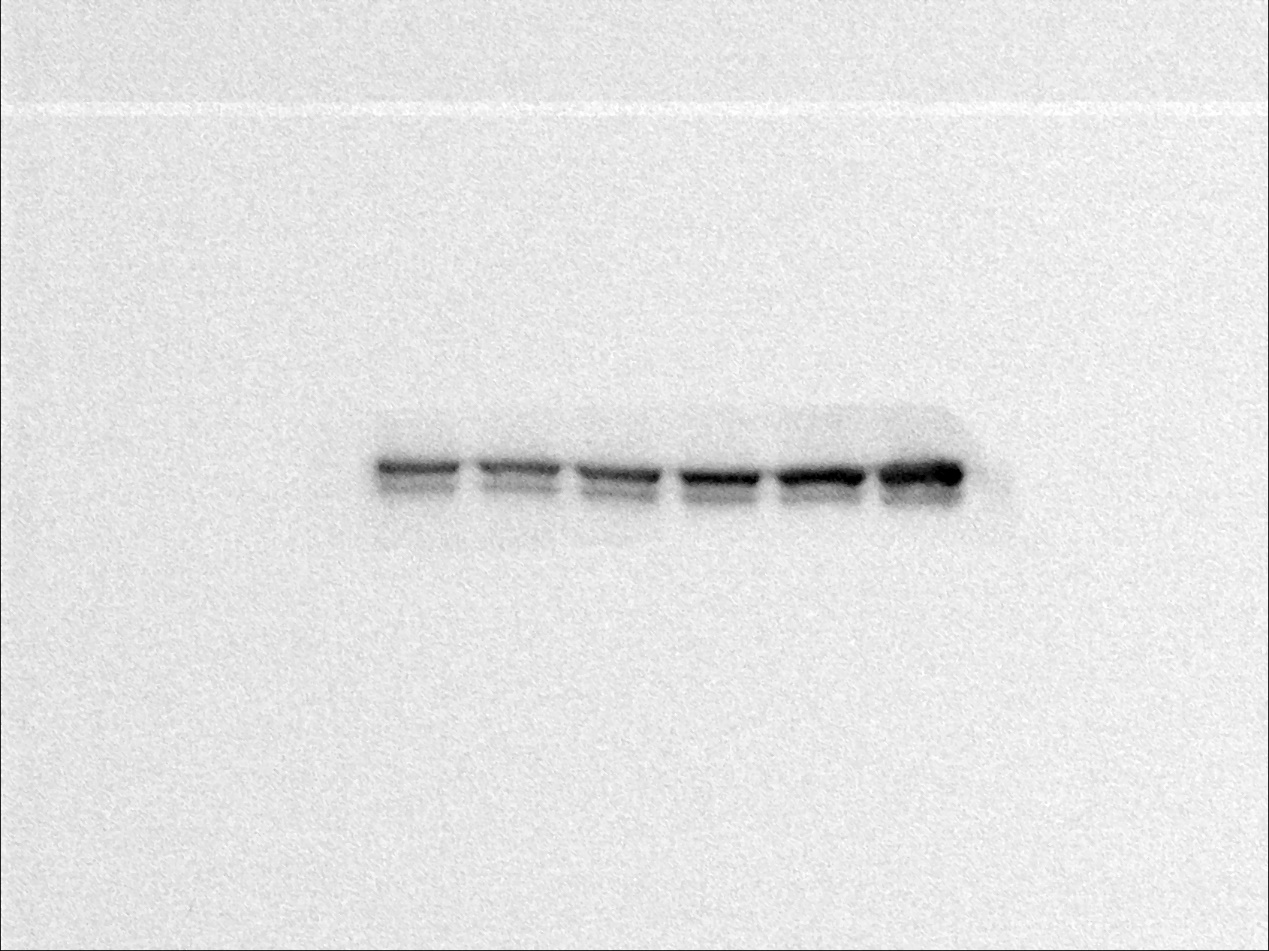


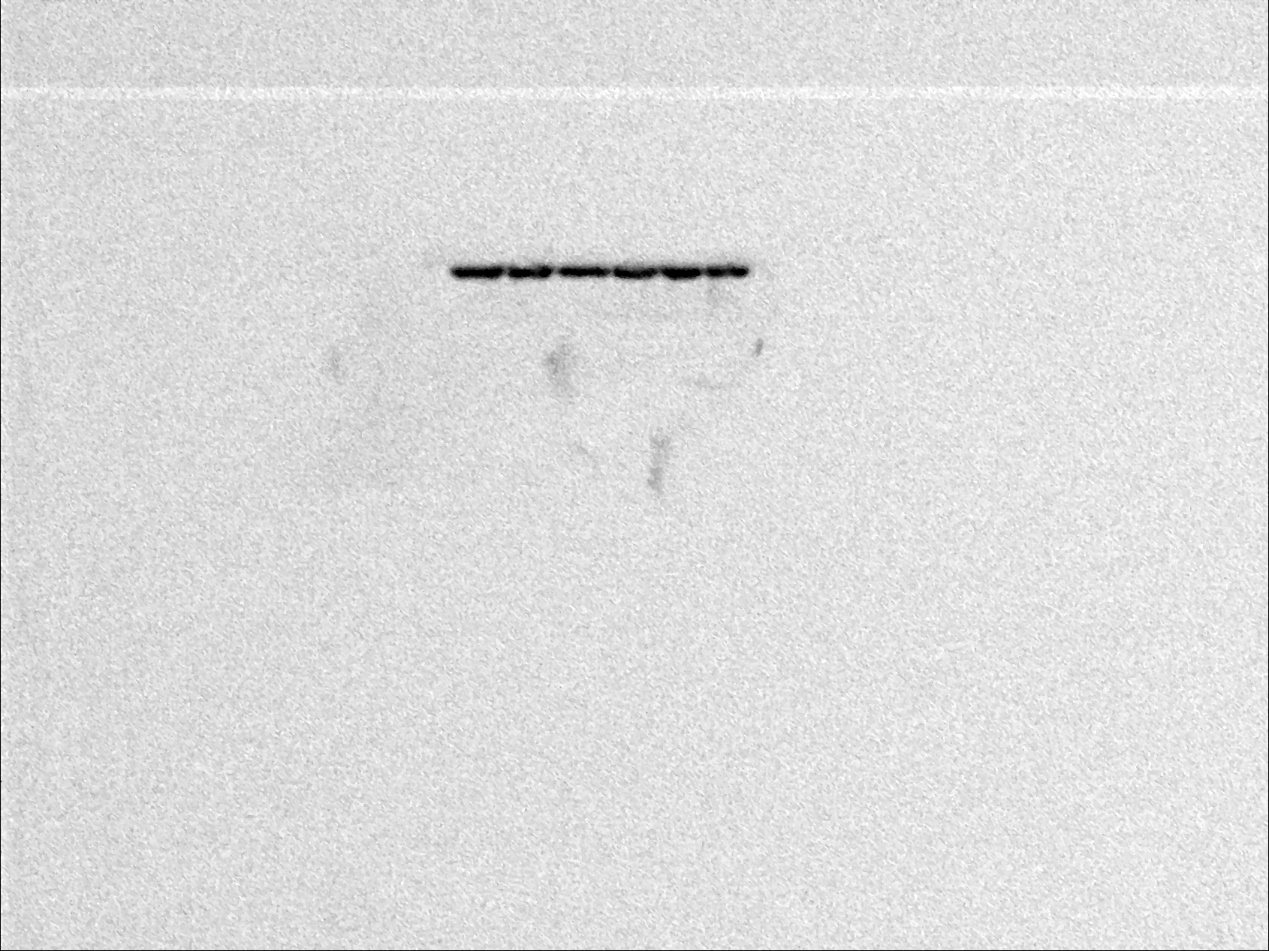


Fig 6O


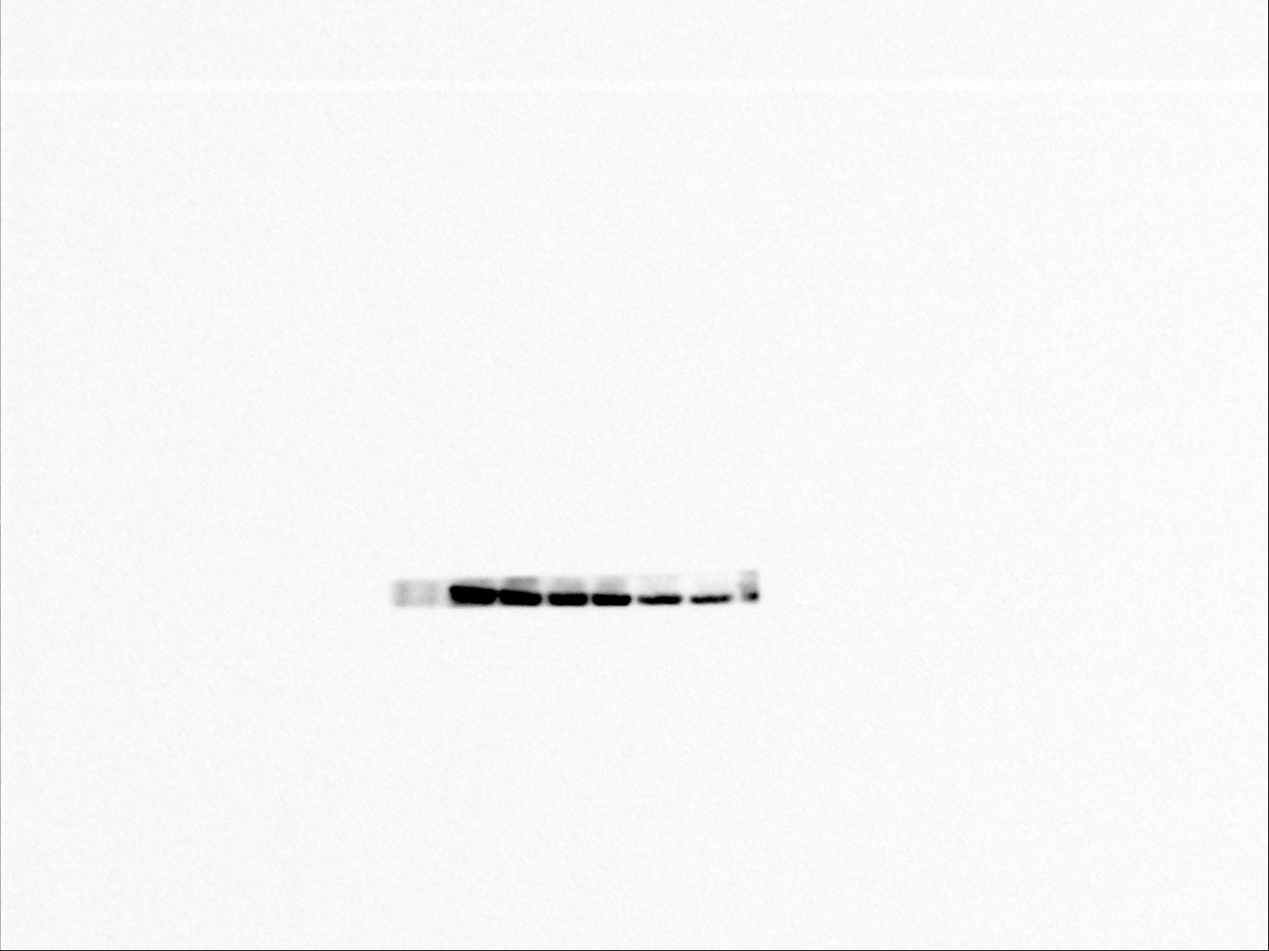


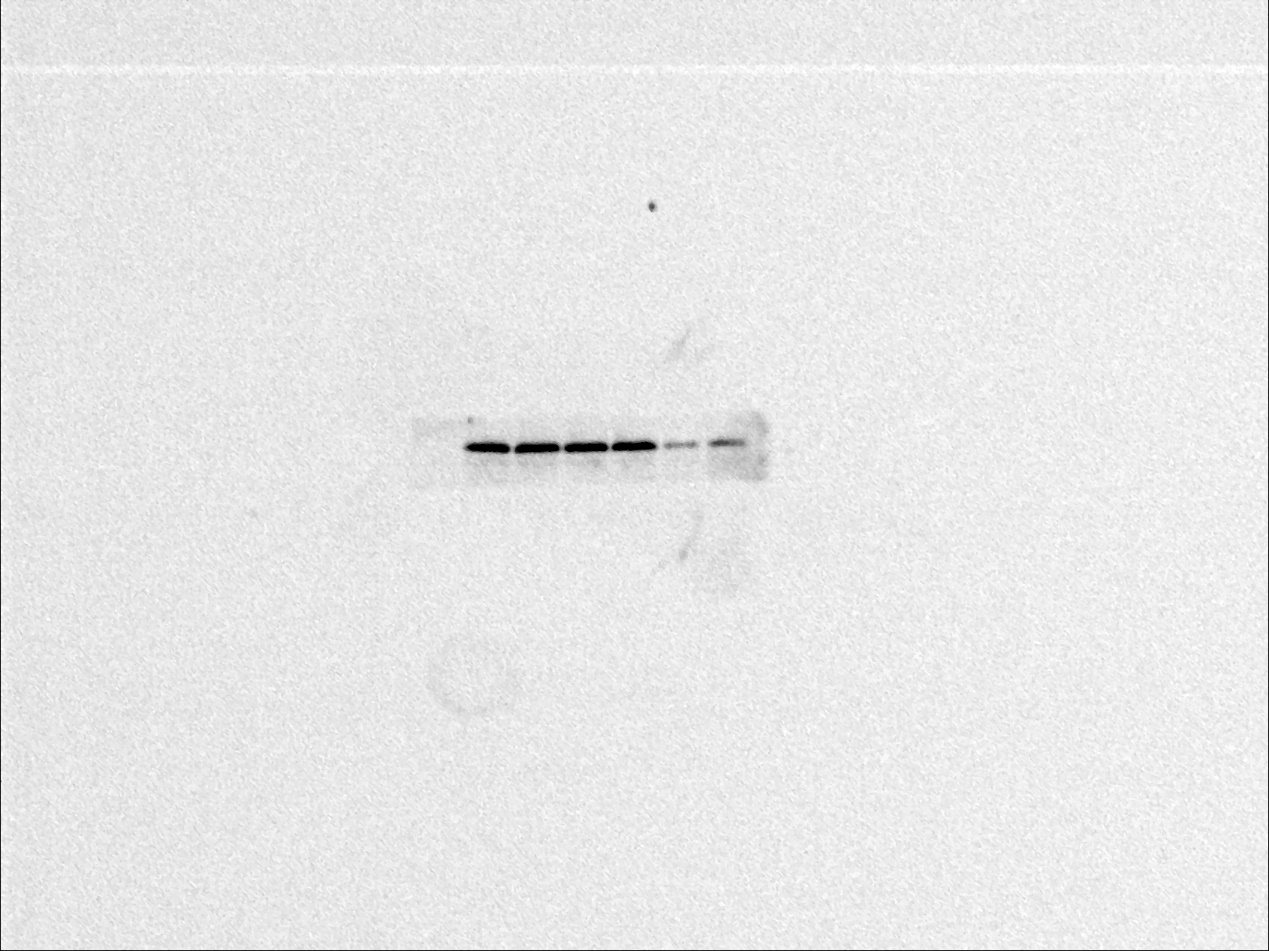


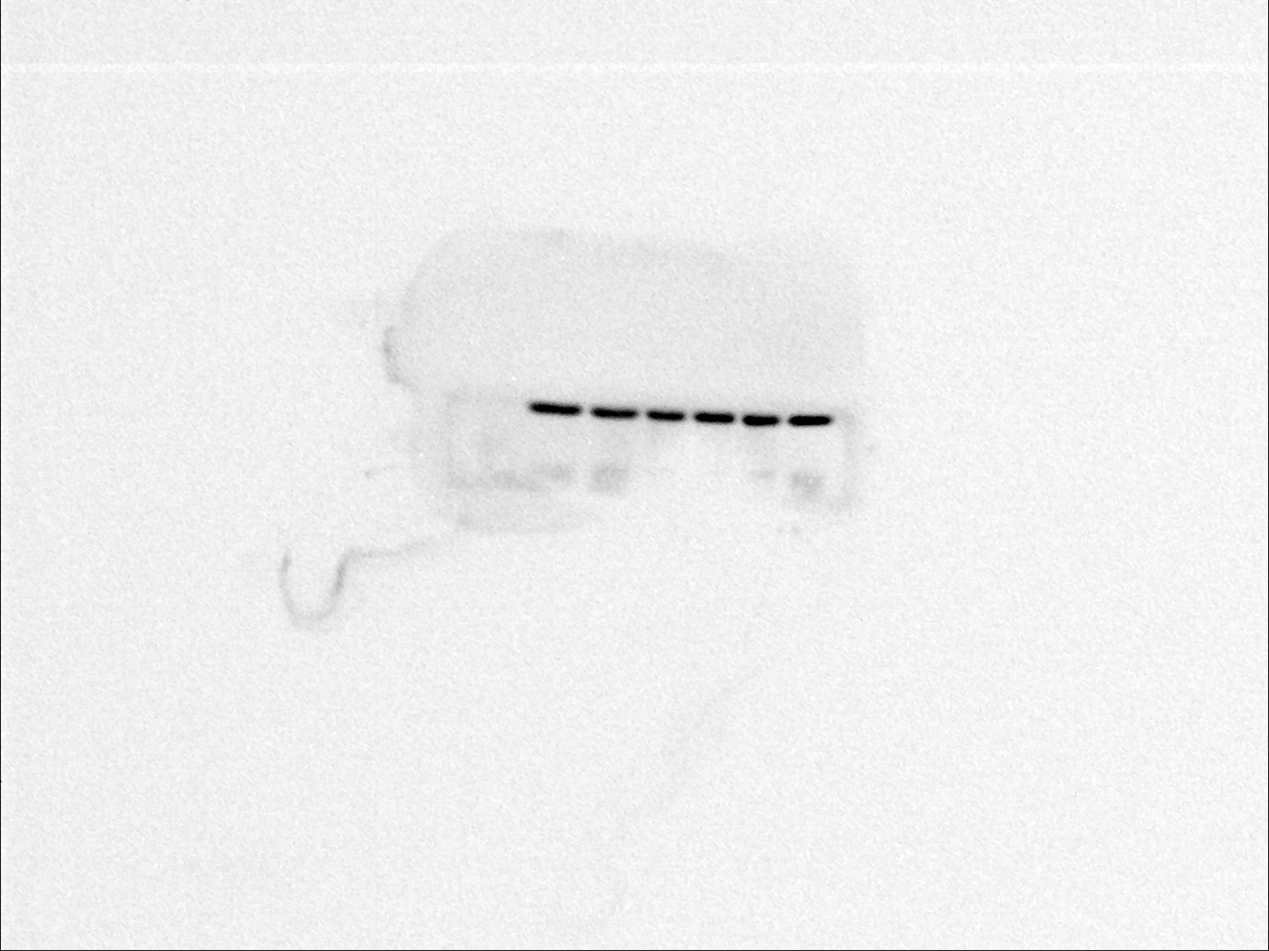

Supplement: Supplementary file 5 — Figure 6 WB Supplementary materials [file 41419_2022_4736_MOESM5_ESM.docx]

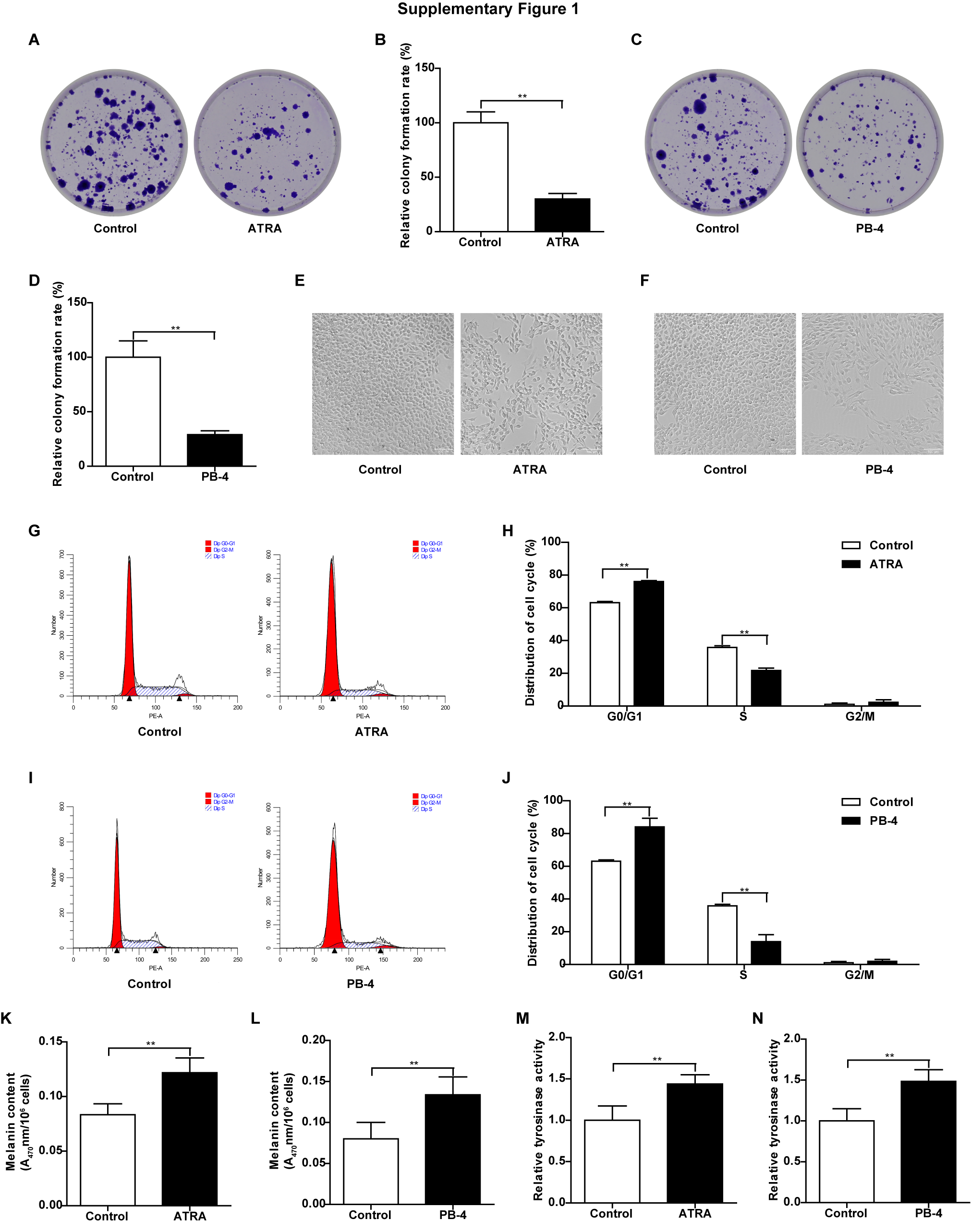

Supplement: Supplementary file 8 — Supplementary Figure 1 [file 41419_2022_4736_MOESM8_ESM.tif]

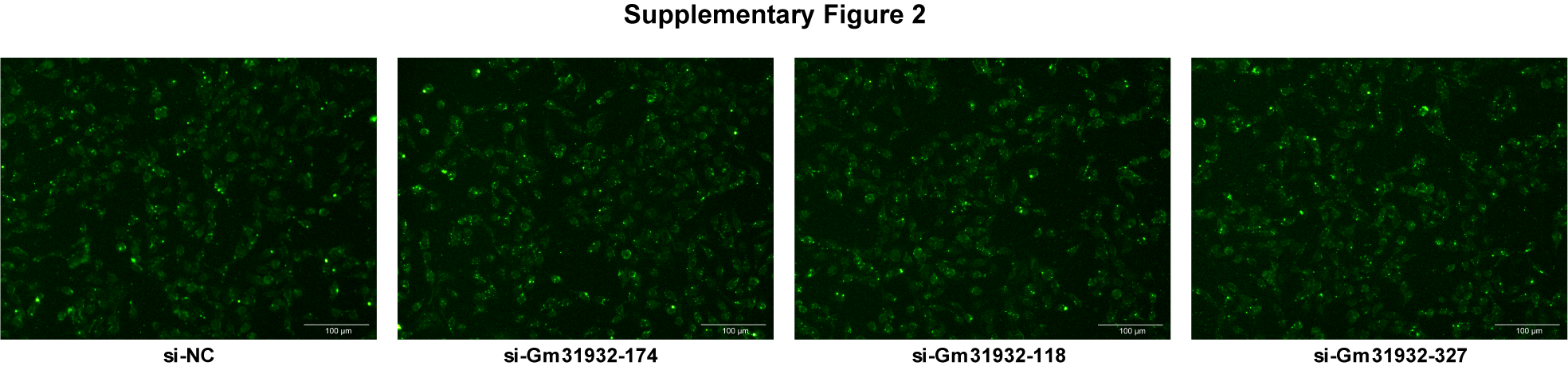

Supplement: Supplementary file 9 — Supplementary Figure 2 [file 41419_2022_4736_MOESM9_ESM.tif]

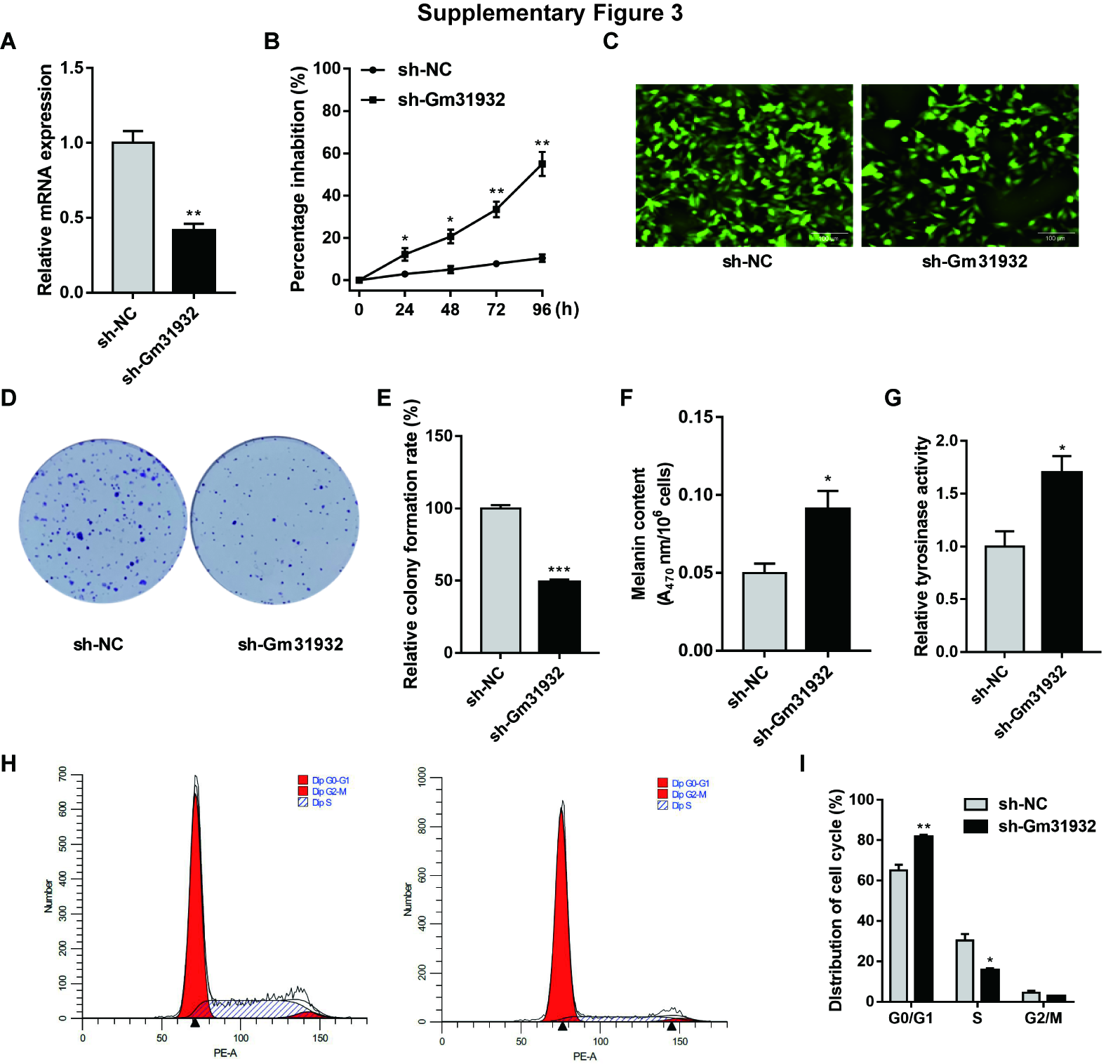

Supplement: Supplementary file 10 — Supplementary Figure 3 [file 41419_2022_4736_MOESM10_ESM.tif]

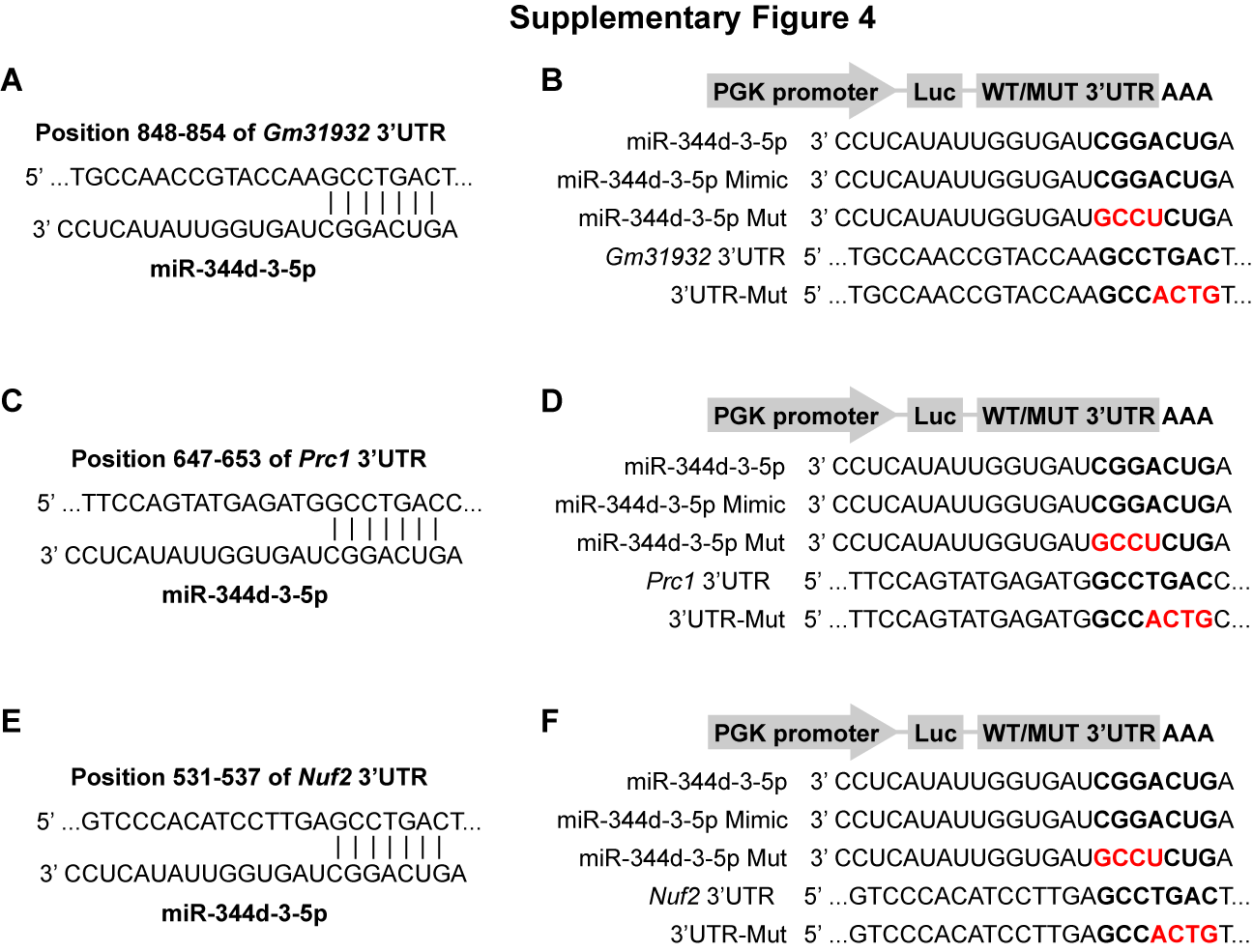

Supplement: Supplementary file 11 — Supplementary Figure 4 [file 41419_2022_4736_MOESM11_ESM.tif]

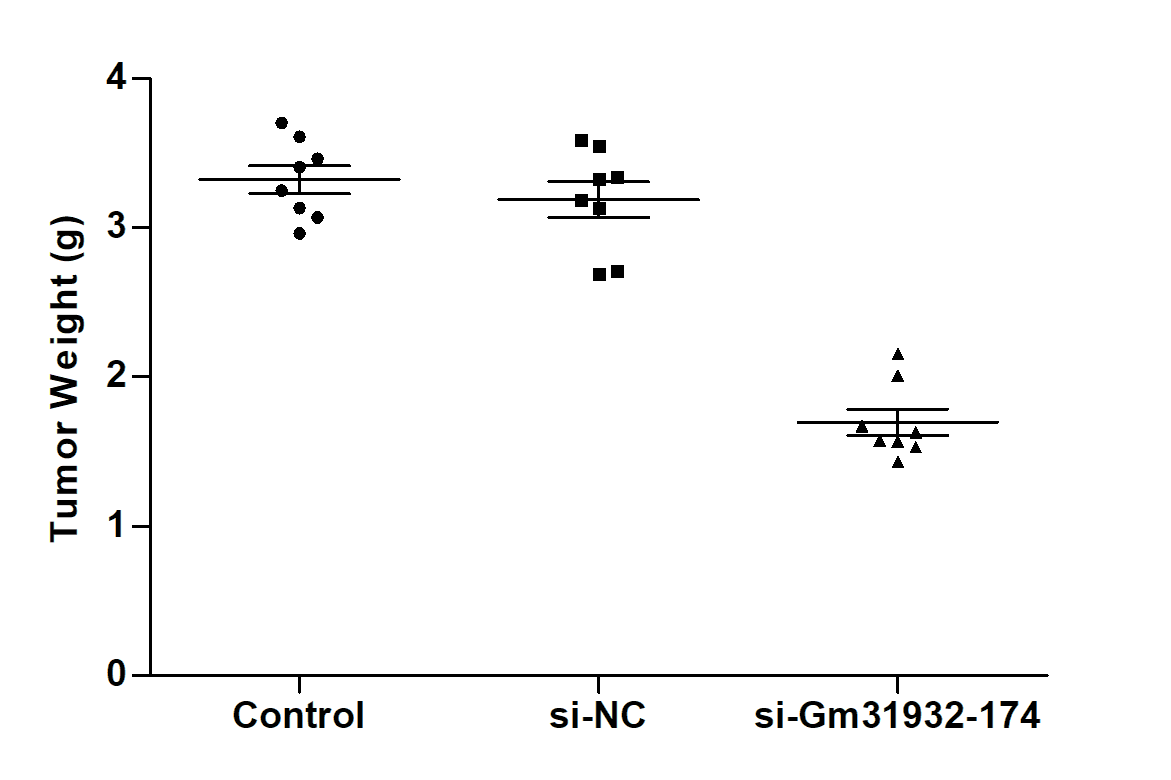

Supplement: Supplementary file 12 — Supplementary Figure 5 [file 41419_2022_4736_MOESM12_ESM.tif]

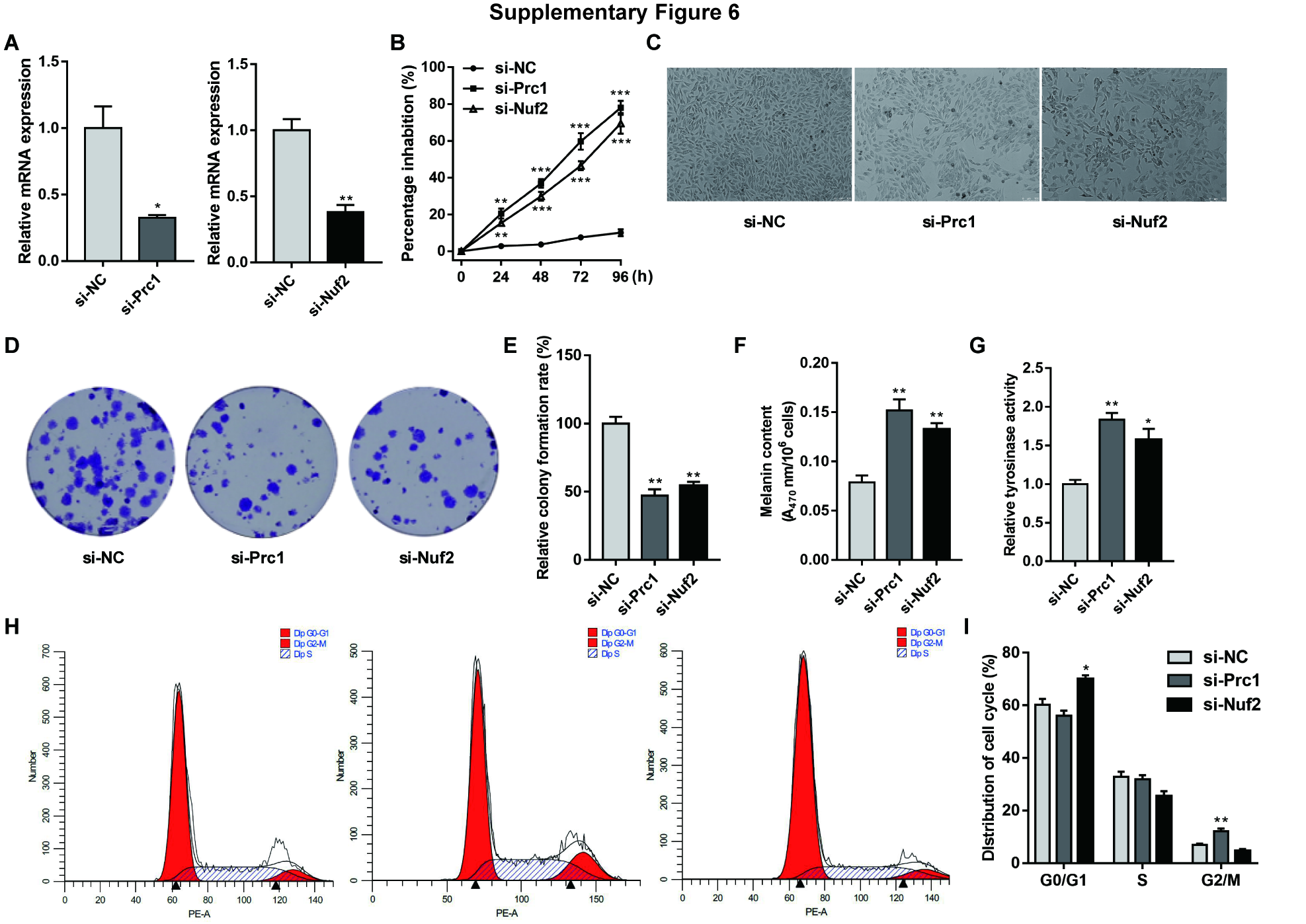

Supplement: Supplementary file 13 — Supplementary Figure 6 [file 41419_2022_4736_MOESM13_ESM.tif]

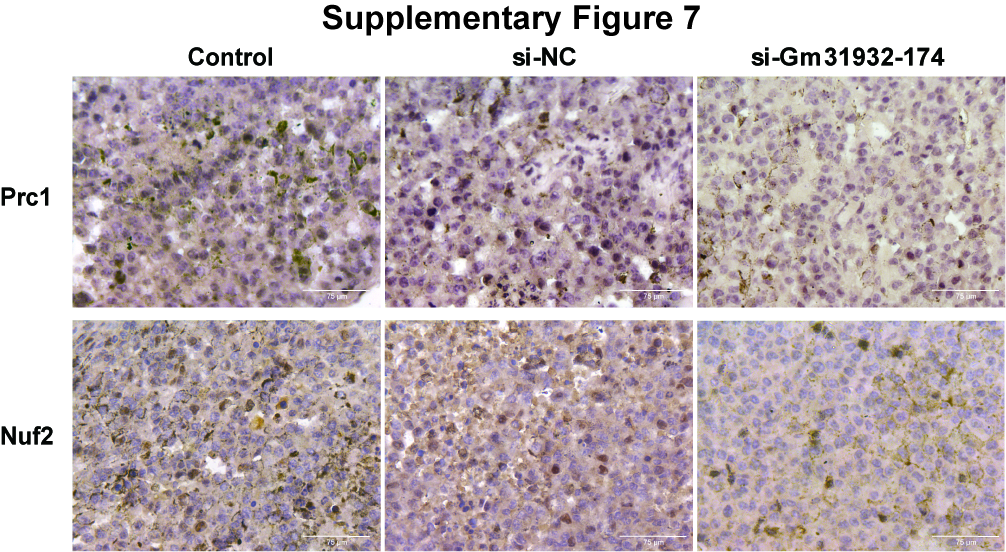

Supplement: Supplementary file 14 — Supplementary Figure 7 [file 41419_2022_4736_MOESM14_ESM.tif]
