# Supplementary material for: A noncoding regulatory RNA Gm31932 induces cell cycle arrest and differentiation in melanoma via the miR-344d-3-5p/Prc1 (and Nuf2) axis
Source: Cell Death Dis. 2022 Apr 7;13(4):314. doi: 10.1038/s41419-022-04736-6 (PMC8990078; doi:10.1038/s41419-022-04736-6)
Supplement: Supplementary file 6 — Supplementary Table 1 [file 41419_2022_4736_MOESM6_ESM.docx]

| Supplementary Table 1   \| **LncRNAs, miRNAs, mRNAs analysis data list** \| \| \| \| \| --- \| --- \| --- \| --- \| \|  \| lncRNAs \| miRNAs \| mRNAs \| \| ATRA *vs* Control \| 102 \| 24 \| 761 \| \| PB *vs* Control \| 306 \| 105 \| 2567 \| \| Commom molecule \| 33 \| 8 \| 393 \|   Supplementary Table 2  **Sequences used in RNA transfection** | | |
| --- | --- | --- | --- | --- | --- | --- | --- | --- | --- | --- | --- | --- | --- | --- | --- | --- | --- | --- | --- | --- | --- | --- |
| siRNA |  |  |
| lncRNA-Gm31932-118 | Sense | 5' -GGAUUAUCACAGAAAUAUACA- 3' |
| lncRNA-Gm31932-118 | Anti-sense | 5' -UAUAUUUCUGUGAUAAUCCAG- 3' |
| lncRNA-Gm31932-327 | Sense | 5' -AGAUGAUGAGAAUCAAUAACC- 3' |
| lncRNA-Gm31932-327 | Anti-sense | 5' -UUAUUGAUUCUCAUCAUCUUU- 3' |
| lncRNA-Gm31932-174 | Sense | 5' -GGCAAUGUUUGCUUGUUCAGA- 3' |
| lncRNA-Gm31932-174  Prc1  Prc1  Nuf2  Nuf2 | Anti-sense  Sense  Anti-sense  Sense  Anti-sense | 5' -UGAACAAGCAAACAUUGCCUG- 3'  5' -GGAUAUGAUGAUUGCUGAAGA- 3'  5' -UUCAGCAAUCAUCAUAUCCAG- 3'  5' -GAGAAGUUAAAGAAUUAUAAA- 3'  5' -UAUAAUUCUUUAACUUCUCUG- 3' |
| NC | Sense | 5' -UUCUCCGAACGUGUCACGUTT- 3' |
| NC | Anti-sense | 5' -ACGUGACACGUUCGGAGAATT- 3' |
| miRNA |  |  |
| mmu-miR-344d-3-5p mimic | Sense | 5' -AGUCAGGCUAGUGGUUAUACUCC- 3' |
| mmu-miR-344d-3-5p mimic | Anti-sense | 5' -GGAGUAUAACCACUAGCCUGACU- 3' |
| miRNA mimic NC | Sense | 5' -UCACAACCUCCUAGAAAGAGUAGA- 3' |
| miRNA mimic NC | Anti-sense | 5' -UACUCUUUCUAGGAGGUUGUGAUU- 3' |
| mmu-miR-344d-3-5p inhibitor |  | 5' -GGAGUAUAACCACUAGCCUGACU- 3' |
| miRNA inhibitot NC |  | 5' -UCUACUCUUUCUAGGAGGUUGUGA- 3' |

Supplementary Table 3

Primers used in this study

| mouse Gm31932 | Forward | GGTAGCTGGAGACAGGCAA |
| --- | --- | --- |
| mouse Gm31932 | Reverse | TCTGAAGTGGAACAGGGGT |
| mouse Gm9939 | Forward | TTCACTTTCTGCGGGATTG |
| mouse Gm9939 | Reverse | TCGGACGAGGACGTTTGGT |
| mouse Gm31365 | Forward | CCGCCCTAATATCCCCATC |
| mouse Gm31365 | Reverse | GTCTCAACCTTCACCCGCA |
| mouse Gm32403 | Forward | GCAGGTGCACTGAACACA |
| mouse Gm32403 | Reverse | CAGCAACACTTCGCATAAA |
| mouse Gm33085 | Forward | TTTCCCTGTGAGTTAGTCTTTG |
| mouse Gm33085 | Reverse | TTGTGTATTTTCCCCCCTTC |
| mouse Gm36355 | Forward | AGAAAGGCATACAATGAAGGG |
| mouse Gm36355 | Reverse | GAGAAGTGGGAGACCAGAAAA |
| mouse Gm36569 | Forward | AAAGTGCTTCCATTGCCCT |
| mouse Gm36569 | Reverse | CCCTTCTCTGAGATTCCCC |
| mouse Gm36673 | Forward | GAATAGAGGGTAGGCGATTGC |
| mouse Gm36673 | Reverse | TTTTCTGTGCTTGACTGGTGA |
| mouse Gm38621 | Forward | CAGGTCCGTGAGTCGGTTA |
| mouse Gm38621 | Reverse | TGTGTGTGTGCTGGTTTGG |
| mouse Gm39215 | Forward | CCCCCTGGATGTGTTCTTA |
| mouse Gm39215 | Reverse | GCTCCTGTTTGCCTGATTG |
| mouse Gm41056 | Forward | TTTGAGTTTGAGCACAGTGGT |
| mouse Gm41056 | Reverse | AAGAGGGTGGTAGAAGATAGGA |
| mouse PRC1 | Forward | CAGATGAGTCTATCACATGCCTG |
| mouse PRC1 | Reverse | CCTCGGTTCTTTGTAGCCTCT |
| mouse NUF2 | Forward | TCCCCAGATACAATGTAGCTGA |
| mouse NUF2 | Reverse | CCGGACTCCATACACTAACTGT |
| mouse GAPDH | Forward | AGGTCGGTGTGAACGGATTTG |
| mouse GAPDH  human PRC1  human PRC1  human NUF2  human NUF2  human GAPDH  human GAPDH | Reverse  Forward  Reverse  Forward  Reverse  Forward  Reverse | TGTAGACCATGTAGTTGAGGTCA  ATCACCTTCGGGAAATATGGGA  TCTTTCTGACAGACGGATATGCT  GGAAGGCTTCTTACCATTCAGC  GACTTGTCCGTTTTGCTTTTGG  ACAACTTTGGTATCGTGGAAGG  GCCATCACGCCACAGTTTC |

Supplementary Table 4

**FISH probes**

| mouse Gm31932 | 5'-TG*TG*TG*AGTCCCAGATATCGGGACTCA*C*A*G-3' |
| --- | --- |
| mouse 18s rRNA | 5'-T*AT*G*CTACCTGGCAGGATCAACCG*A*G*T-3' |
