# Supplementary material for: A noncoding regulatory RNA Gm31932 induces cell cycle arrest and differentiation in melanoma via the miR-344d-3-5p/Prc1 (and Nuf2) axis
Source: Cell Death Dis. 2022 Apr 7;13(4):314. doi: 10.1038/s41419-022-04736-6 (PMC8990078; doi:10.1038/s41419-022-04736-6)
Supplement: Supplementary file 7 — Supplementary Table 2 [file 41419_2022_4736_MOESM7_ESM.docx]

**Raw data for Figure 1G**

| **Groups** | **CT** |
| --- | --- |
| GAPDH | 14.67±0.41 |
| Gm31932 | 17.51±1.74 |
| Gm9939 | 22.26±1.13 |
| Gm31365 | 24.14±1.22 |
| Gm32403 | 21.53±1.97 |
| Gm33085 | 28.27±5.08 |
| Gm36355 | 22.81±2.10 |
| Gm36569 | 21.06±1.55 |
| Gm36673 | 22.13±1.50 |
| Gm38621 | 22.31±1.27 |
| Gm39215 | 25.09±1.45 |
| Gm41056 | 18.75±1.05 |
